# stMMR: accurate and robust spatial domain identification from spatially resolved transcriptomics with multimodal feature representation

**DOI:** 10.1093/gigascience/giae089

**Published:** 2024-11-28

**Authors:** Daoliang Zhang, Na Yu, Zhiyuan Yuan, Wenrui Li, Xue Sun, Qi Zou, Xiangyu Li, Zhiping Liu, Wei Zhang, Rui Gao

**Affiliations:** Center of Intelligent Medicine, School of Control Science and Engineering, Shandong University, Jinan 250061, China; Center of Intelligent Medicine, School of Control Science and Engineering, Shandong University, Jinan 250061, China; Institute of Science and Technology for Brain-Inspired Intelligence, Center for Medical Research and Innovation, Shanghai Pudong Hospital, Fudan University Pudong Medical Center, Fudan University, Shanghai 200433, China; MOE Key Lab of Bioinformatics and Bioinformatics Division of BNRIST, Department of Automation, Tsinghua University, Beijing 100084, China; Center of Intelligent Medicine, School of Control Science and Engineering, Shandong University, Jinan 250061, China; Center of Intelligent Medicine, School of Control Science and Engineering, Shandong University, Jinan 250061, China; School of Software Engineering, Beijing Jiaotong University, Beijing 100044, China; Center of Intelligent Medicine, School of Control Science and Engineering, Shandong University, Jinan 250061, China; Center of Intelligent Medicine, School of Control Science and Engineering, Shandong University, Jinan 250061, China; Center of Intelligent Medicine, School of Control Science and Engineering, Shandong University, Jinan 250061, China

**Keywords:** spatially resolved transcriptomics, domain identification, multimodal integration, geometric deep learning, similarity contrastive learning

## Abstract

**Background:**

Deciphering spatial domains using spatially resolved transcriptomics (SRT) is of great value for characterizing and understanding tissue architecture. However, the inherent heterogeneity and varying spatial resolutions present challenges in the joint analysis of multimodal SRT data.

**Results:**

We introduce a multimodal geometric deep learning method, named stMMR, to effectively integrate gene expression, spatial location, and histological information for accurate identifying spatial domains from SRT data. stMMR uses graph convolutional networks and a self-attention module for deep embedding of features within unimodality and incorporates similarity contrastive learning for integrating features across modalities.

**Conclusions:**

Comprehensive benchmark analysis on various types of spatial data shows superior performance of stMMR in multiple analyses, including spatial domain identification, pseudo-spatiotemporal analysis, and domain-specific gene discovery. In chicken heart development, stMMR reconstructed the spatiotemporal lineage structures, indicating an accurate developmental sequence. In breast cancer and lung cancer, stMMR clearly delineated the tumor microenvironment and identified marker genes associated with diagnosis and prognosis. Overall, stMMR is capable of effectively utilizing the multimodal information of various SRT data to explore and characterize tissue architectures of homeostasis, development, and tumor.

## Introduction

The advancement in spatially resolved transcriptomics (SRT) technologies has opened new avenues for a deeper understanding of the spatial architecture and functionality of tissues. Currently, many SRT technologies have been developed, such as imaging-based and sequencing-based methods [1–7]. Among these, techniques such as 10x Genomics Visium not only provide the spatial location and gene expression data for each spot but also acquire high-resolution hematoxylin and eosin (H&E)–stained histology images of the tissue section, revealing richer information about the tissue organization. These technological advancements offer new insights into the characterization of tissue architecture, enabling a more comprehensive understanding of tissue development and disease pathogenesis [7–9].

For SRT technologies capable of providing both gene expression data and histology images, the information from these different modalities reflects the structural information of tissues at various levels. Gene expression profiles reflect the difference of cell state between spots [10]. Spatial location information provides the precise location of each spot. Histological images display morphological features of cells, such as size and shape [11]. Although each of these modalities has its own strength, they complement each other, together forming a more comprehensive picture of tissue architecture. For instance, changes in gene expression are reflected not only at the molecular level but may also manifest in histological images as morphological alterations [12]. Furthermore, the issues of sparsity and dropout in SRT data can be effectively addressed through integrating histological image data [13]. By leveraging the interdependence between gene expression and morphological features, as well as the similarity in gene expression patterns among adjacent spots, we can enhance spatial signals and characterize tissue structure.

However, the joint representation of multimodal features in SRT is challenging. First, these different modalities inherently possess significant heterogeneity. For instance, transcriptomic data are typically high-dimensional, quantified gene expression information, reflecting the gene activity in different spots or cells. In contrast, histology images are 2-dimensional visual data depicting the morphological and structural information of cells at different spots. This fundamental difference makes the direct fusion of these 2 types of modalities difficult. Second, the disparity in data scale and resolution is also a crucial issue. Transcriptomic data reveal unique patterns of gene expression within spots or cells from a microscopic perspective. Conversely, histology images provide more macroscopic information on organization and morphology. This difference in scale complicates the establishment of spatial correspondence. Therefore, there is an urgent need for methods that can effectively integrate multimodal features.

Recently, a variety of cutting-edge computational methods have been developed to effectively address the challenge of joint representation of multimodal SRT data. Specifically, BASS, BayesSpace, and Giotto leverage spatial neighborhood information for enhancing the resolution of SRT data [14–16]. CellCharter and PRECAST incorporate spatial contexts to correct batch effect for a better domain identification [17, 18]. MENDER is a recently proposed multirange cell context decipherer for ultra-fast tissue structure identification [19]. CCST, STAGATE, SpaceFlow, and GraphST utilize graph neural networks (GNNs) to integrate gene expression data with spatial information, achieving effective clustering of spots [20–23]. However, these methods do not employ histology images, failing to fully enhance the interpretability of gene expression data through these images. In contrast, recent pioneering studies like stLearn and DeepST have shown more significant progress [24, 25]. These methods effectively integrate gene expression data with spatial neighborhood information and morphological features extracted from histology images, demonstrating a stronger potential for application. Despite these methods demonstrating capability in processing multimodal information in SRT data, they give less consideration to the complex global spot similarity across distinctive spatial multimodal features. This limitation impedes their ability to accurately characterize spatial patterns and discover functional biological contexts in tissue.

To achieve precise identification of spatial domains, we introduce stMMR, a geometric deep learning method for effectively representing multimodal information in SRT data. stMMR utilizes spatial location information as a bridge to establish adjacency relationships between spots. It encodes gene expression data and morphological features extracted from histological images using graph convolutional networks (GCNs). stMMR proposes a novel strategy to achieve joint learning of intramodal and intermodal features. Within a certain modality, stMMR employs self-attention mechanisms to dynamically learn the complex relationships of different spots. For integrating cross-modal information, stMMR innovatively utilizes similarity contrastive learning along with the reconstruction of gene expression features and adjacency information. This enhances the ability of stMMR to recover information and provides denoising capabilities. We conducted comprehensive tests on different SRT datasets, including samples profiled by 10x Visium, NanoString technology, and spatial transcriptomics (ST) technology. stMMR outperforms state-of-the-art (SOTA) techniques in terms of domain identification, pseudo-spatiotemporal analysis, and domain-specific gene discovery. The experimental results on breast cancer and lung cancer [26] demonstrated that stMMR accurately identifies tumor edges and tumor-infiltrating regions, proving its potential value in clinical research. Overall, the stMMR exhibits exceptional capability in the multimodal feature representation of SRT, providing a powerful new tool for accurate and robust domain identification.

## Methods

### Overview of stMMR

**Figure 1:**
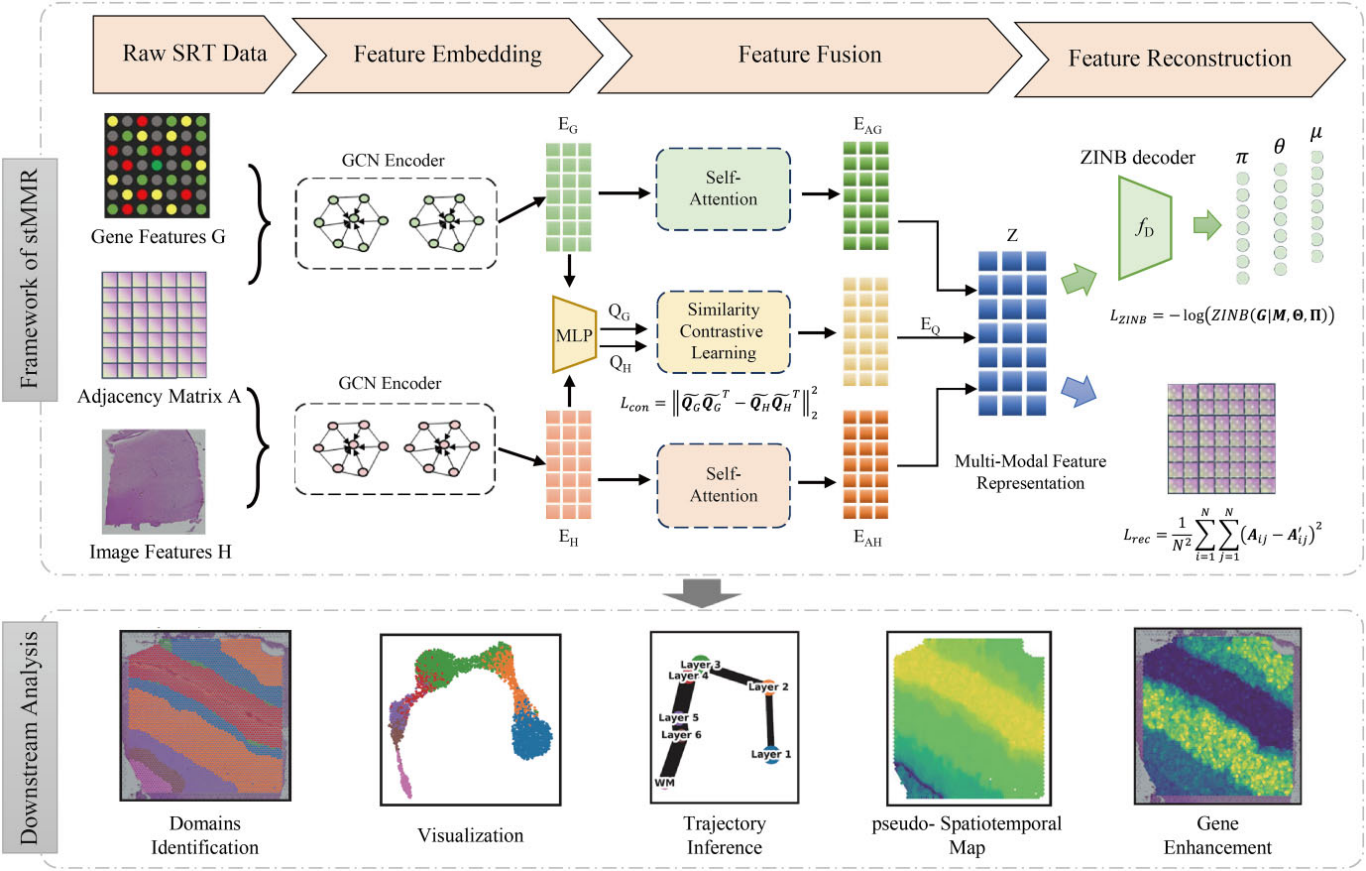
Schematic overview of stMMR for the joint representation of features from different modalities. Gene expression and histology image information are embedded using the GCN module based on the adjacent matrix. Then, the relationships between different modalities are captured through similarity contrast learning, followed by feature fusion. Finally, the original features are reconstructed from the multimodal feature representation. This representation can be used for downstream analysis directly.

The multimodal joint representation process of stMMR primarily consists of the following 3 steps: multimodal feature embedding, feature fusion, and feature reconstruction. The overall workflow of stMMR is illustrated in Fig. 1, and the detailed implementation is introduced in Supplementary Section 1.

### Multimodal feature embedding

The stMMR initially performs embedding on gene expression, spatial location, and histology image information. For gene expression data, $G\in \mathbb {R}^{N\times P}$ represents the normalized gene expression matrix, where $N$ is the number of spots and $P$ is the number of identified high-variance genes. For histological images, we use a pretrained Vision Transformer (ViT) model [27] to extract the image features matrix $H\in \mathbb {R}^{N\times M}$, where $M$ is the output dimension. To encode the spatial location information, we construct an undirected weighted graph to present SRT data, and the adjacency matrix $A$ is defined as


(1) \begin{eqnarray*}
A_{ij}=exp\left(-\frac{d(i,j)^2}{2l^2}\right)\
\end{eqnarray*}

where $d(i,j)$ represents the Euclidean distance between spots $i$ and $j$, and $l$ is used to control the relationship between weight and distance. A larger value of $l$ implies a faster decay of weight with increasing distance.

Next, we employ a 2-layer GCN encoder for message passing and aggregation of image features and gene expression features [28]:


(2) \begin{eqnarray*}
E^{(k)}=\widetilde{D}^{-\frac{1}{2}}\widetilde{A}\widetilde{D}^{-\frac{1}{2}}E^{(k-1)}W^{(k-1)}\
\end{eqnarray*}

where $E^{(k)}$ and $E^{(k-1)}$ represent the input and output of the GCN encoder, respectively. $E^{(0)}$ can be the image features $H$ or gene expression features $G$. $\tilde{A}=A+I$ denotes the symmetrically normalized adjacency matrix, where $I$ is the identity matrix. $\tilde{D}$ and $W^{(k-1)}$ are the weighted degree matrix and trainable parameter, respectively. The visual features and gene expression features obtained after the encoder are denoted as $E_{H}$ and $E_{G}$.

### Feature fusion

We propose a novel strategy for multimodal information aggregation. Biological structures often exhibit relationships between distant regions that cannot be captured by local spatial dependencies alone. For instance, in complex tissues such as the brain or cancerous tissues, cells in nonadjacent spatial domains can share similar gene expression patterns or morphological characteristics. Therefore, stMMR uses a normalized attention module to learn the global relationships between spots in a single modality, as shown in Eq. 3:


(3) \begin{eqnarray*}
E_A=softmax\left(\frac{E\cdot E^T}{\sqrt{d}}\right) \cdot E
\end{eqnarray*}

where $E$ represents the imaging features $E_{H}$ or transcriptomics features $E_{G}$ from the previous step. New features obtained through the attention module are $E_{AH}$ and $E_{AG}$. Notably, a nonlinear activation function and Euclidean distance matrix $d$ normalize the weights, preventing the local optima issue from oversized weights for certain spots [29].

stMMR uses contrastive learning for cross-modal feature fusion, highlighting the consistency between modalities like morphology and gene expression, which have both similarities and complementary relationships [30–32]. It maps the latent features $E_{H}$ and $E_{G}$ through 2 fully connected neural networks to obtain hierarchical representations $Q_{H}$ and $Q_{G}$, as shown in Eq. 4:


(4) \begin{eqnarray*}
Q=Relu(W_QE+b_Q)
\end{eqnarray*}

where $E$ represents the morphological features $E_{H}$ or gene expression features $E_{G}$, and $Q$ corresponds to $Q_{H}$ or $Q_{G}$. $W_{Q}$ and $b_{Q}$ are the parameters of the fully connected network.

After obtaining low-dimensional features $Q_{H}$ and $Q_{G}$ for the 2 modalities, a fully connected neural network is employed to fuse them, as shown in Eq. 5:


(5) \begin{eqnarray*}
E_Q=W_E\cdot concat(Q_G,Q_H)+b_E\
\end{eqnarray*}

where $W_{E}$ and $b_{E}$ are the parameters of the fully connected network.

To enhance the consistency between $Q_{H}$ and $Q_{G}$, we use a constraint as shown in Eq. 6, replacing the loss of traditional contrastive learning:


(6) \begin{eqnarray*}
L_{con}=\left\Vert \widetilde{Q_G}\widetilde{Q_G}^T-\widetilde{Q_H}\widetilde{Q_H}^T\right\Vert _2^2\
\end{eqnarray*}

where $\widetilde{Q_G}$ and $\widetilde{Q_G}$ are the normalization matrices of $Q_{G}$ and $Q_{H}$, respectively.

Finally, we further integrate modality-specific features $E_{AH}$ and $E_{AG}$ obtained from Eq. 3 with the cross-modality features $E_{Q}$ obtained from Eq. 5 to get the multimodal feature representation $Z$, as shown in the following equation:


(7) \begin{eqnarray*}
Z=\alpha E_Q+\beta E_{AH}+\gamma E_{AG}\
\end{eqnarray*}

where $\alpha$, $\beta$, and $\gamma$ are hyperparameters for adjusting the importance of features.

### Feature reconstruction

stMMR adopts the zero-inflated negative binomial (ZINB) decoder [33, 34] to reconstruct gene expression information, and the adjacency matrix is estimated directly using the concept of a graph auto-encoder [35, 36]:


(8) \begin{eqnarray*}
L_{ZINB}=-\log \left(ZINB(G|M,\Theta ,\Pi )\right)\
\end{eqnarray*}

(9) \begin{eqnarray*}
A^{^{\prime }}=Sigmoid\left(\frac{Z \cdot Z^T}{\Vert Z\Vert _2\cdot \Vert Z^T\Vert _2}\right)\
\end{eqnarray*}

where $M$, $\Theta$, and $\Pi$ are the mean, dispersion, and dropout probability of the output from network, respectively.

Subsequently, the regularization loss between the reconstructed matrix and the adjacency matrix can be computed:


(10) \begin{eqnarray*}
L_{rec}=\frac{1}{N^{2}}\sum _{i=1}^{N}\sum _{j=1}^{N}\left(A_{ij}-A_{ij}^{^{\prime }}\right)^{2}\
\end{eqnarray*}

### Objective function

Finally, we integrated Eq. 6, Eq. 8, and Eq. 10 to formulate the final objective function:


(11) \begin{eqnarray*}
L=a*L_{con}+b*L_{ZINB}+c*L_{rec}\
\end{eqnarray*}

In this equation, a, b, and c are weight for the different loss terms.

For detailed information on the training process and parameter settings, please refer to the Supplementary Section 1.

### Benchmark methods

To demonstrate the effectiveness of the multimodal feature representation in SRT data, we selected 7 SOTA methods for benchmarking comparison. These methods include SCANPY [37], which utilizes only gene expression data; CCST, STAGATE, GraphST, and SpaceFlow, which employ both gene expression and spatial location information [20–23]; and stLearn and DeepST, which incorporate all 3 modalities [24, 25]. Methods that have already been compared in previous works are not included in our analysis [38–40].

## Results

### stMMR enhances detection of stratified architectural patterns in human dorsolateral prefrontal cortex tissue

**Figure 2:**
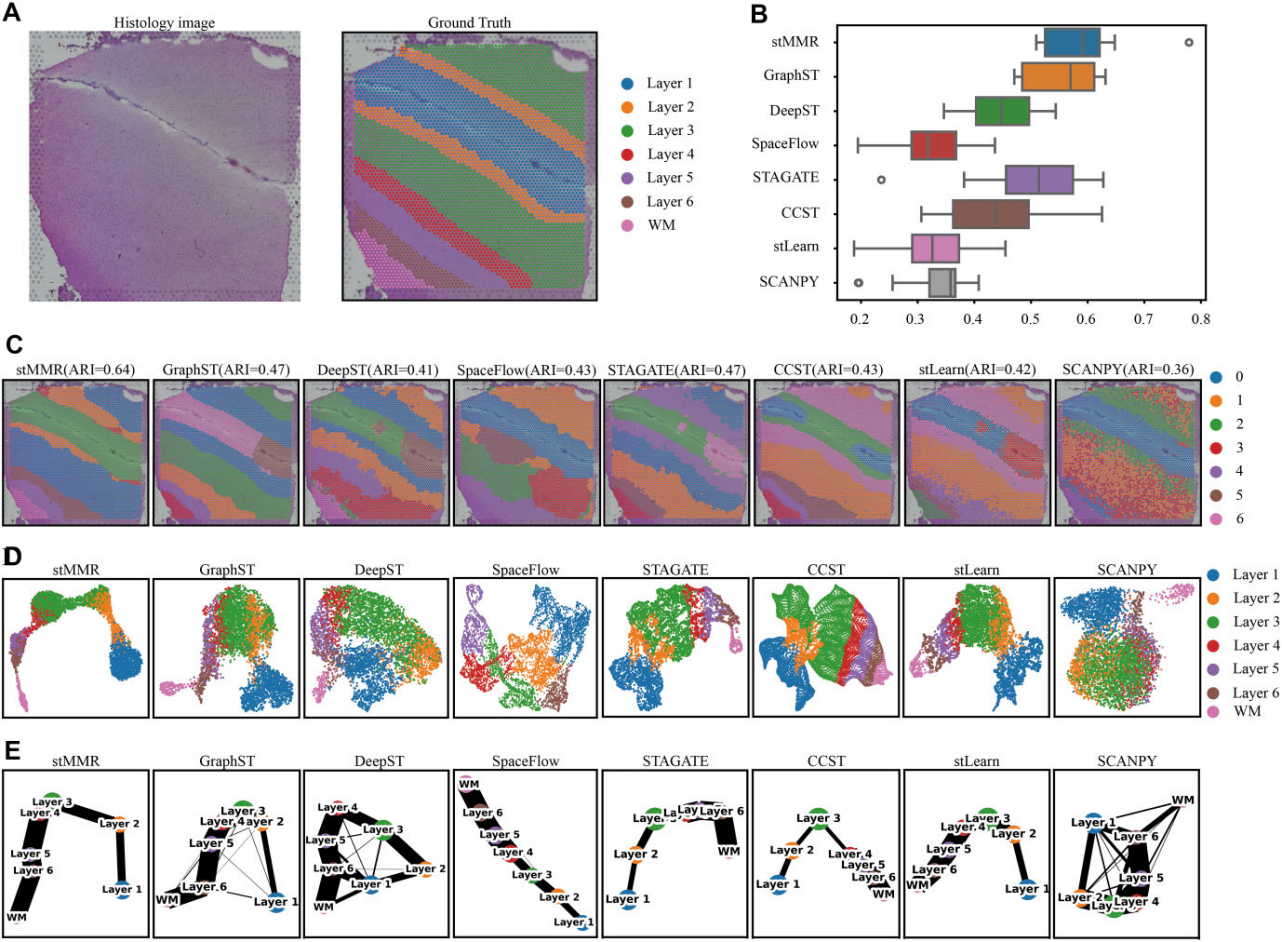
Performance comparisons of different methods on DLPFC datasets. (A) The histology image and manually annotated brain regions of slice 151509. (B) The overall performance of 8 different methods across 12 slices. (C) The domain recognition results on slice 151509. (D) The UMAP visualization results of the embeddings from 8 different methods on slice 151509. (E) The inferenced trajectories on slice 151509.

The spatial structure of the brain is closely related to its function, particularly evident in the layered organization of the human brain cortex [41]. To explore the spatial structure arrangement of brain, we collected a 10x Visium dataset containing 12 dorsolateral prefrontal cortex (DLPFC) sections [42]. The histology image and manually annotated layers are illustrated in Fig. 2A.

We initially compared the Adjusted Rand Index (ARI) levels of various methods across 12 slices of the DLPFC dataset (Fig. 2B). The result reveals that stMMR outperformed other methods, achieving the highest ARI and the smallest variance compared to manual annotations. Notably, the results from STAGATE, CCST, and SpaceFlow show differences in the ARI across different slices, indicating that these methods are more sensitive to the domain patterns. Scanpy uses only gene expression information and shows the poorest performance. Methods like stlearn and DeepST, which integrate histological information, are outperformed by GraphST and STAGATE. This underperformance might stem from insufficient integration of transcriptomic and imaging data.

Next, we conducted a detailed analysis for each slice (Fig. 2C and Supplementary Fig. S1). To demonstrate the results, we used slice 151509 as an example (Fig. 2C–E). The results shows that DeepST struggles with rough segmentation between layers. CCST, SpaceFlow, and stLearn have issues with erroneous region identification. Although GraphST and STAGATE accurately discern the arrangement of different regions, these methods exhibit biases in identifying the boundaries between distinct domains. In this specific case, stMMR demonstrates exceptional domain identification results. We further utilize UMAP for low-dimensional visualization analysis of the results obtained from different methods (Fig. 2D) to verify whether the embeddings can accurately encompass information on regional arrangement and boundaries. The analysis reveals that techniques such as stMMR, CCST, STAGATE, and stLearn effectively separate different domains. In contrast, GraphST, SpaceFlow, and DeepST exhibit noticeable issues in layer boundaries. For instance, the boundaries between layers 2, 3, and 4 are confused.

Further, we conducted a detailed trajectory inference using the PAGA algorithm [43] for these methods (Fig. 2E). The PAGA graphs indicate that stMMR, STAGATE, CCST, and stLearn perform well in predicting trajectory between adjacent layers. The other methods display confused results in this analysis.

Combining the insights from these various analyses, it is evident that stMMR is remarkably effective in domain identification and trajectory inference. These results adequately demonstrate effective capability of stMMR in integrating transcriptomic and histological data.

### stMMR enhances spatial gene expression profiling and structural characterization

In SRT, the analysis of domain-specific genes holds significant importance. However, identifying domain-specific genes that have relationships with histological structures is challenging. This is primarily due to the presence of substantial noise in the gene expression profiles generated by SRT techniques, such as the dropout event [44–46]. To validate whether stMMR can enhance gene expression data through histological information, we analyzed domain-specific genes identified using the original gene expression profile and the profile reconstructed through the ZINB decoder [47].

Using both original and reconstructed gene expression data, genes such as *AQP4* and *HPCAL1* are recognized as layer-specific genes. These genes are enriched in multiple layers and have been confirmed through multiplex single-molecule fluorescence in situ hybridization [42]. However, employing reconstructed gene expression data facilitates the identification of new domain-specific genes. For instance, with the enhancement of stMMR, *CACNA2D2* and *ADCYAP1* can be identified as domain-specific genes in layer 3. Previous research has found that in layer 3 of primates, the *CACNA2D2* gene exhibits differential expression and is closely associated with several biological pathways, including calcium signaling and synaptic long-term depression [48]. *ADCYAP1* has also been proved to be a domain-specific gene in a former study [49]. This suggests that the expression patterns after stMMR enhancement are more consistent with known neurobiological functions and pathological states.

**Figure 3:**
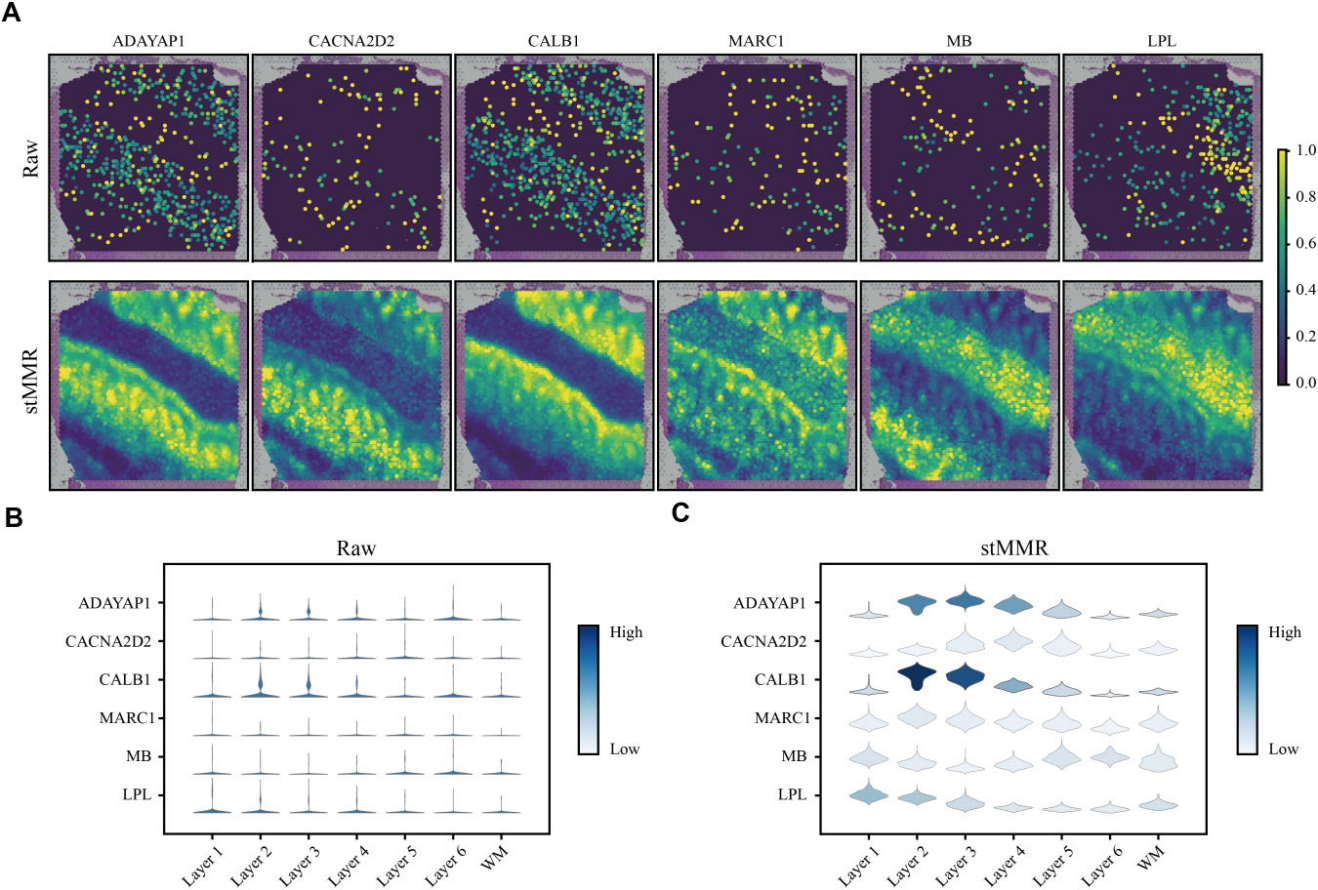
stMMR enhances spatial gene expression profiles and spatial structural characterization. (A) Spatial representation of layer-specific marker genes before and after data enhancement. (B) Gene expression level before and after data enhancement.

We also conducted a more detailed analysis by combining gene expression levels with their spatial locations (Fig. 3). We found that after enhancement with stMMR, more distinct expression patterns of domain-specific genes can be observed. Specifically, Fig. 3A demonstrates a clear spatial representation of domain-specific marker genes (*ADAYAP1, CACNA2D2, CALB1, MARC1, MB*, and *LPL*) after data enhancement. In the original data, the expression pattern of genes such as *ADCYAP1, CACNA2D2*, and *MB* is sparse, and the boundaries in spatial regions are blurred, making it difficult to discern a clear expression pattern (Fig. 3A, B). However, after enhancement with stMMR, we can observe that *CACNA2D2* and *CALB1* exhibit much clearer expression patterns in layers 2 and 3. Additionally, the enrichment of *LPL* in layer 1 becomes more pronounced (Fig. 3A, and C). These results reflect that stMMR not only improves the spatial resolution of gene expression patterns using histological information but also enhances our understanding of the subtle differences in gene expression across different regions of the brain.

### stMMR deciphers evolving cell lineage structures in the chicken heart ST dataset

Analyzing temporal SRT data can reveal the dynamic domain changes during the development of tissue organs. We collected the chicken heart SRT dataset to further investigate the effectiveness of stMMR in the integrated representation of multimodal features [50]. This dataset includes 12 tissue slices, collected on day 4 (5 slices), day 7 (4 slices), day 10 (2 slices), and day 14 (1 slices), documenting 4 key stages of the Hamburger–Hamilton ventricular developmental stages [50]. We selected SpaceFlow as the baseline because it was specifically designed to capture spatiotemporal dynamics.

**Figure 4:**
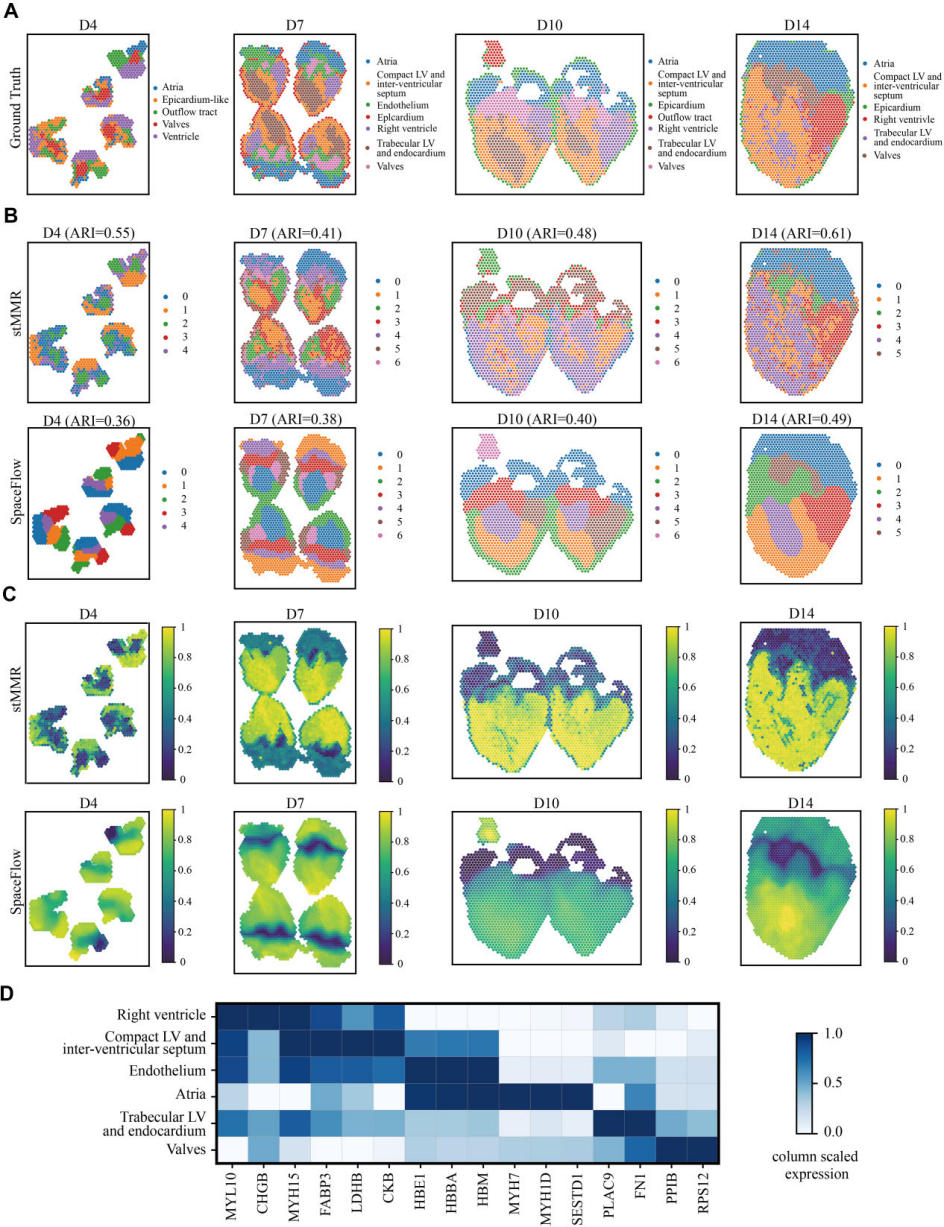
stMMR reveals cell lineage structures during chicken heart development. (A) The ground-truth label provided by the original data. (B) The domains recognized by stMMR and SpaceFlow. (C) The plots of pSM value from stMMR and SpaceFlow for illustrating the pseudo-temporal developmental trajectory. (D) The differentially expressed marker genes discovered by stMMR.

We annotated the slices of different developmental stages using labels provided by the original research (Fig. 4A) [50]. Subsequently, we employed the embeddings from stMMR and SpaceFlow to identify domains of chicken heart across these 4 distinct stages. Fig. 4B indicates that the regions detected by stMMR largely coincide with manual annotation. For instance, major regions of the chicken heart, such as atrial cells and the interventricular septum, are accurately identified. Notably, stMMR also detects domains that are hard to identify (Fig. 4B). For example, in the data from days 7, 10, and 14, the epicardium, a thin layer surrounding the outer side of the chicken heart, is clearly identified by stMMR. Although there are some instances of misclassification in the characterization of spot features using stMMR in a few regions, the identification of the epicardium is quite clear (Fig. 4B).

Next, we adopted a method similar to a previous study to analyze the pseudo-spatiotemporal map (pSM) [22]. In brief, we mapped the spot features obtained through stMMR and SpaceFlow on the pseudo-temporal axis [22, 37, 51]. These points reflect the relative positions of cells in their developmental trajectory or functional state. As clearly visible in Fig. 4C, within D7 to D14, the valve structures can be distinctly identified through the pSM values. Moreover, the representation of the myocardium in ventricles, as indicated by the pSM values, appears more uniform (yellow area) compared to the regional segmentation results in Fig. 4B. According to related research [52], the endocardium, the inner layer of the heart, is one of the early events in cardiac formation. The endocardial tubes are fundamental to cardiac development, eventually merging to form the primitive heart tube. As the heart tube forms, myocardial development commences, followed closely by the development of the atria. In our analysis, we observe that the myocardium in ventricles (yellow area in Fig. 4C) consistently shows higher pSM values compared to other areas in the same stage, indicating a later pseudo-temporal ordering of the ventricular myocardium [22]. Additionally, the pseudo-temporal ordering of the atria (marked in teal) follows that of the valves, suggesting that the development of the atria occurs after the valves. Therefore, the pSM derived from stMMR accurately displays the developmental sequence of the chicken heart. We further identified domain-specific genes through differential expression analysis across regions. For instance, we observed that *MYH7* is highly specifically expressed in the atria (Fig. 4D). This finding aligns with previous reports on the analysis of atria- and ventricle-specific proteins [ 53].

### stMMR accurate identifies tumor region in human breast cancer

**Figure 5:**
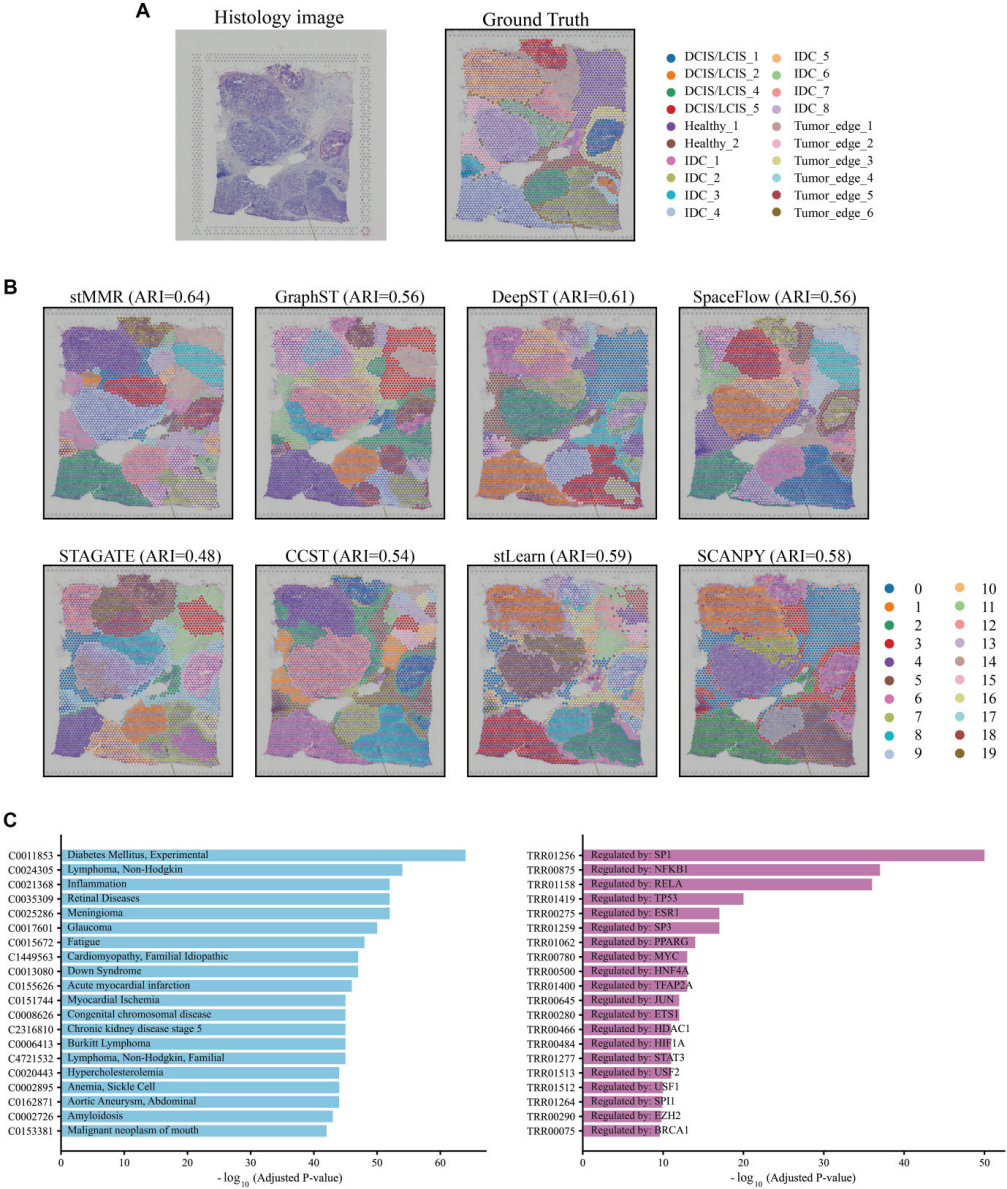
stMMR identifies tumor region in the human breast cancer dataset. (A) The H&E images and the manually annotated regions. (B) The annotation results from different methods. (C) Top 20 differentially expressed gene enriched terms identified by DisGeNET (left panel) and TRRUST (right panel).

Breast cancer is a major type of cancer worldwide [54]. We collected a human breast cancer dataset from the 10x Visium platform to conduct an in-depth analysis of the microenvironment in breast cancer (Fig. 5A).

First, we applied different methods for domain identification. From the results presented in Fig. 5B, it is observed that stMMR shows the most outstanding performance in category labeling. In terms of regional continuity, stMMR also demonstrates superior performance among different methods. Taking the IDC_5 area in the upper left corner as an example, this area occupies a significant portion in invasive ductal carcinoma, with a notable increase in cancer cells compared to normal tissue or nontumorous areas [23]. However, only stMMR accurately identified the entire IDC_5 area, demonstrating higher precision compared to other methods. Additionally, stMMR also exhibits higher continuity in predicting the Tumor_edge area, whereas the results of other methods appear more dispersed in this aspect.

Next, we conducted a comprehensive analysis of domain-specific genes between merged tumor and normal regions (Supplementary Section 2.3). We utilized the DisGeNET to delve into the domain-specific genes of tumor regions [55]. Our analysis revealed that these domain-specific genes are enriched in several breast cancer–related terms such as non-Hodgkin lymphoma and inflammation (Fig. 5C). Studies have shown that the development of breast cancer significantly increases the risk of non-Hodgkin lymphoma, particularly follicular lymphoma and mature T/NK-cell lymphomas [56]. Numerous studies also have indicated that inflammation plays a regulatory role in the development of cancer and its response to treatment [57–59]. To further validate our research findings, we conducted an analysis of the transcriptional regulatory network using TRRUST [60]. The results indicated that multiple top-ranked terms are closely associated with breast cancer (Fig. 5C). For instance, the key regulatory factors (*SP1, NFKB1, RELA*, and *TP53*) from the top 4 terms have been confirmed to play pivotal roles in the development and progression of breast cancer [61–66].

### stMMR dissects cell-type differences in a lung cancer SRT dataset based on NanoString technology

**Figure 6:**
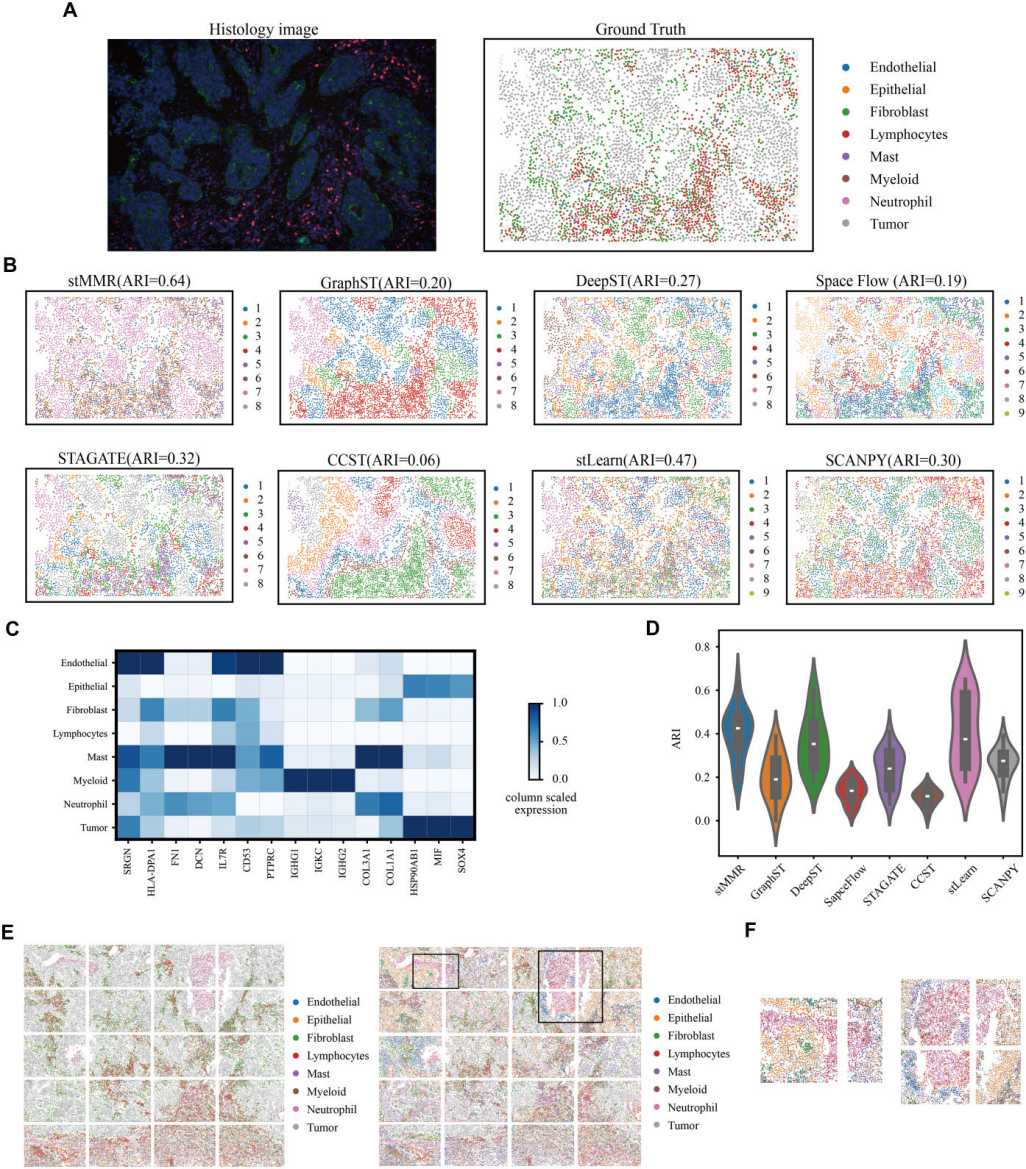
stMMR recognizes cell-type differences in a lung cancer dataset. (A) One FOV of the lung cancer SRT data. (B) Cell types identified by different methods. (C) Expression pattern of marker genes for different cell types. (D) The overall performance of different methods across 20 FOVs. (E) Cell types annotated manually in 20 FOVs. And cell types annotated by stMMR in 20 FOVs. (F) The zoomed-in results of boundaries between adjacent FOVs identified by stMMR.

To further validate the generalization ability and applicability of stMMR, we applied stMMR to the single-cell SRT dataset generated by NanoString CosMx SMI. This dataset comprises lung cancer tissue samples from 20 fields of view (FOVs) [26] and covering 8 major cell types (Fig. 6A, E).

We employed the benchmarking methods to identify spatial domains within 20 FOVs, as shown in Fig. 6B, D–F. Fig. 6B revealed that stMMR closely aligns with the original study in detecting the spatial distribution of cell types, particularly in identifying tumor cells. In the overall analysis of the 20 sections, the performance of stMMR is superior to other methods (Fig. 6D). Furthermore, we conducted a cell type–specific gene analysis based on the cell annotations in 1 slice. We observe that different cells exhibit unique expression patterns (Fig. 6C). For instance, *IGKC* transcripts, previously reported to be upregulated in myeloid progenitor populations, are also confirmed in our study [67]. The genes *COL3A1* and *COL1A1* show significant positive correlations with neutrophils [68, 69]. Additionally, the oncogene *SOX4* is prominently featured in our differential analysis of tumor cells [70]. These genes are also identified as diagnostic or prognostic biomarkers in previous studies [68, 71–74]. Notably, some cell types also share similar gene expression patterns (Fig. 6C). For example, epithelial cells and tumor cells exhibit expression similarities. Multiple studies using single-cell transcriptomics analysis have revealed that lung cancer cells share characteristics similar to those of type 1 and type 2 alveolar epithelial cells [75, 76]. This similarity may be related to lung cancer cells maintaining epithelial cell functions, such as cell adhesion and migration [77, 78].

We also conducted a visualization analysis comparing the results of stMMR applied to 20 tissue sections with the actual division of tissue regions. The analysis demonstrates that stMMR effectively identifies tissue regions across multiple sections (Fig. 6E). Notably, even in regions bisected by section boundaries, stMMR maintains smooth and continuous performance (Fig. 6E, F). These findings indicate that the joint representation of stMMR not only effectively eliminates noise from different data types but also maintains excellent performance in the recognition of tissue regions across multiple slices.

## Discussion

SRT technology enables us to deeply understand the spatial structure of tissues within biological systems from multiple dimensions, including gene expression profiles, spatial location, and histological information. However, the inherent data heterogeneity along with the varying spatial resolutions presents challenges in the integration of these modalities. To harmonize and unify multimodal data as well as achieve effective joint representation for multimodal SRT data, we propose a novel computational framework, stMMR.

stMMR effectively unifies gene expression profiles and histological information by utilizing spatial location as a connecting link. This method automates the construction of adjacency relationships between neighboring spots. Then, GCN is employed to extract features from both gene expression profiles and histological information. Furthermore, stMMR adopts an innovative strategy for representing intramodal and intermodal features. Initially, it employs an attention mechanism for an in-depth learning within a single modality. It then integrates cross-modality features through a combination of similarity contrastive learning, along with the reconstruction of gene expression and adjacency relationship. By applying stMMR to SRT data of various tissues and resolutions, we have validated its exceptional performance in multiple analyses, including domain identification, pseudo-spatiotemporal analysis, gene expression data enhancement, and the identification of domain-specific genes.

The remarkable performance of stMMR can be attributed to several innovative designs, as confirmed by ablation studies (Supplementary Fig. S2). The crucial aspect is the integration of histological information with gene expression data through spatial location. In SRT, gene expression data suffer from issues of sparsity and zero inflation, which are key factors that interfere with downstream analysis [33, 79]. Previous research has shown that histological information can predict gene expression data [30–32]. Therefore, compared to methods that rely only on gene expression information, stMMR integrates imaging information and exhibits superior performance in spot characterization. Second, unlike other methods that construct spatial transcriptomic data as unweighted graphs, stMMR builds undirected weighted graphs inversely proportional to Euclidean distances between spots, better reflecting the influence of spatial distance on message passing and aggregation. Furthermore, the consideration of relationships within and between modalities is also crucial. Sole reliance on gene expression data for correlation analysis may result in information loss. In contrast, methods that incorporate imaging information, such as DeepST, focus primarily on the integration of multimodal data, overlooking the relationships within individual modalities. To fully leverage the relationships within and between modalities, stMMR not only uses similarity contrastive learning for integrating features across modalities but also incorporates a self-attention module for deep embedding of features within a modality. Additionally, the reconstruction modules for gene expression and adjacency matrix further encourage the model to retain as much original information as possible. This encoder–decoder structure improves the ability of stMMR to recover information and also endows stMMR with denoising capabilities and robustness.

Notably, stMMR possesses strong scalability. It can be easily applied to other data derived from diverse experimental techniques. Beyond the previously mentioned datasets, we applied stMMR to the analysis of a mouse brain dataset derived using 10x Visium technology, a human pancreatic ductal adenocarcinoma dataset obtained through ST technology, and a human colorectal cancer dataset generated using the newly introduced 10x Visium HD technology (Supplementary Figs. S3, S4, and S5). In these tests, the stMMR consistently achieved optimal results. Furthermore, we extended stMMR to integrate and analyze multislice data (Supplementary Fig. S6). Additionally, by duplicating the gene expression module, the framework of stMMR can be directly used for integration of features from proteomes or epigenomics [80–83].

There is still room for the improvement of stMMR. Currently, stMMR employs Euclidean distance in the construction of spot adjacency matrices. However, in practical scenarios, it may be more rational to utilize different distance metrics for graph construction based on modal features. For instance, considering gene expression data, the use of Pearson correlation coefficients or Kullback-Leibler (KL) divergence might be more appropriate to measure expression similarity between spots. In contrast, for spatial imaging data, either Euclidean distance or staining similarity can serve as the distance metric. Under these circumstances, the constructed graph transitions from being a homogeneous graph to a heterogeneous one. For such heterogeneous graphs with multiple types of edges, we can apply methods like metapath2vec or multiview learning to achieve embedding and integration of different modalities [84–87].

In this article, we introduce a robust and accurate tool, stMMR, for the integration of gene expression data, spatial information, and histological information from SRT data. Compared to existing methods, stMMR demonstrates a significant advantage in integrating multimodal data, particularly excelling in domain identification, pseudo-spatiotemporal analysis, and domain-specific gene analysis. Overall, as an effective and user-friendly tool, stMMR enhances the multimodal joint analysis of SRT data, providing substantial support for research in relevant fields.

## Availability of Source Code and Requirements

Project name: stMMR

Project homepage: https://github.com/nayu0419/stMMR

Operating system(s): Linux

Programming language: Python

Other requirements: Python 3.9.1 or higher

License: MIT license

RRID: SCR_025601

BiotoolsID: stmmr

## Additional Files

**Supplementary Fig. S1**. The results of the regions identified by stMMR in 12 DLPFC slices.

**Supplementary Fig. S2**. Ablation study results for stMMR on a breast cancer dataset. (A) The spatial clustering result of stMMR. (B) stMMR without histological information, resulting in a lower ARI of 0.57, indicating the importance of histology in improving spatial domain identification. (C) stMMR without GCN, which yields a significantly reduced ARI of 0.49, showing the critical role of GCN in capturing local spatial relationships. (D) stMMR without the attention mechanism, leading to a decrease in ARI to 0.60, demonstrating the importance of global attention in enhancing overall model performance.

**Supplementary Fig. S3**. The domain recognition results of the 10× mouse brain dataset. (A) The histology image and (B) manually annotated tissue structures. (C) The domain recognition results by different methods.

**Supplementary Fig. S4**. The domain recognition results of human pancreatic ductal adenocarcinoma. (A) The histology image and (B) annotated tissue structures. (C) The domain recognition results by different methods.

**Supplementary Fig. S5**. Spatial clustering results on the human colorectal cancer. (A) The histology image. (B) The domain recognition results by different methods.

**Supplementary Fig. S6**. Spatial domain identification results from stMMR integrating 4 slices and the ground truth for the 4 slices.

**Supplementary Table S1**. Summary of the datasets used in this study.

## Abbreviations

ARI: Adjusted Rand Index; CRC: colorectal cancer; DLPFC: dorsolateral prefrontal cortex; GCN: graph convolutional network; H&E: hematoxylin and eosin; pSM: pseudo-spatiotemporal map; SRT: spatially resolved transcriptomics; ViT: Vision Transformer; ZINB: zero-inflated negative binomial.

## Supplementary Material

giae089_Supplementary_Files

giae089_GIGA-D-24-00153_Original_Submission

giae089_GIGA-D-24-00153_Revision_1

giae089_Response_to_Reviewer_Comments_Original_Submission

giae089_Reviewer_1_Report_Original_Submission Shihua Zhang -- 7/3/2024

giae089_Reviewer_1_Report_Revision_1 Shihua Zhang -- 10/8/2024

giae089_Reviewer_2_Report_Original_Submission Hongzhi Wen -- 7/18/2024

## Data Availability

All datasets used in this article are publicly available and listed in Supplementary Section 2. Processed datasets are also available at SODB (https://gene.ai.tencent.com/SpatialOmics/) and can be downloaded by PySODB (https://protocols-pysodb.readthedocs.io/en/latest/). The domain-specific genes found by stMMR are available on the stMMR GitHub page(https://github.com/nayu0419/stMMR).
