# Supplementary material for: stMMR: accurate and robust spatial domain identification from spatially resolved transcriptomics with multimodal feature representation
Source: Gigascience. 2024 Nov 28;13:giae089. doi: 10.1093/gigascience/giae089 (PMC11604062; doi:10.1093/gigascience/giae089)
Supplement: giae089_Supplementary_Files [file giae089_supplementary_files.zip › Supplementary Materials.docx]

**Supplementary Materials for**

**stMMR:** **accurate and robust spatial domain identification from spatially resolved transcriptomics with multi-modal feature representation**

Daoliang Zhang^1,†^, Na Yu^1,†^, Zhiyuan Yuan^2,†^, Wenrui Li^3^, Xue Sun^1^, Qi Zou^1^, Xiangyu Li^4^, Zhiping Liu^1^, Wei Zhang^1,*^, Rui Gao^1,*^

^1^ Center of Intelligent Medicine, School of Control Science and Engineering, Shandong University, Jinan, Shandong 250061, China

^2^ Institute of Science and Technology for Brain-Inspired Intelligence, Center for Medical Research and Innovation, Shanghai Pudong Hospital, Fudan University Pudong Medical Center, Fudan University, Shanghai 200433, China

^3^ MOE Key Lab of Bioinformatics and Bioinformatics Division of BNRIST, Department of Automation, Tsinghua University, Beijing 100084, China

^4^ School of Software Engineering, Beijing Jiaotong University, Beijing 100044, China

^†^These authors contributed equally

*To whom correspondence should be addressed. Email: gaorui@sdu.edu.cn, zw@sdu.edu.cn

**Supplementary Notes**

# stMMR

## 1.1 Introduction

Accurately deciphering the spatial landscapes and transcriptional profiles of complex tissues from spatial transcriptomics data is an essential approach to comprehensively understand the distribution of cells in space and their complex interactions with the surrounding environment. Here, we proposed a PyTorch-based toolkit named stMMR for effective joint characterization of cross-modal data in spatial transcriptomics.

For more information, please see the [stMMR github page](https://github.com/nayu0419/stMMR).

## 1.2 The Detailed Implementation of stMMR

#### 1.2.1 Multi-modal feature embedding

The stMMR initially performs embedding on gene expression, spatial location, and histology image information. We begin by assuming the presence of SRT data comprising N spots. For tissue histological images, we extract pixel features corresponding to each spot using a pre-trained Vision Transformer (ViT) model [1], resulting in a feature matrix $\boldsymbol{H}\in\mathbb{R}^{N\times M}$, where $M$ is the output dimension of the pre-trained ViT model (Supplementary Section 1.3). For gene expression data, we employ SeuratV3 to filter high variance genes and perform a log transformation on the expression levels of these genes, denoted as $\boldsymbol{G}\in\mathbb{R}^{N\times P}$, where $P$ represents the number of high variance genes identified. Additionally, we encode the spatial location information of each spot, resulting in a position encoding matrix corresponding to each spot. Specifically, we used an undirected weighted graph to present SRT data. For any two spots, we posit that the closer their spatial distance, the greater their similarity. Consequently, we define the adjacency matrix $\boldsymbol{A}$ between any two spots $i$ and $j$ as follows:

$$\begin{aligned} \boldsymbol{A}_{\boldsymbol{ij}}=exp\left( -\frac{{d\left( i,j \right)}^{2}}{{2l}^{2}} \right)\#\left( 1 \right) \end{aligned}$$

where $d\left( i,j \right)$ represents the Euclidean distance between spots $i$ and $j$, and $l$ is a hyperparameter controlling the relationship between weight and distance. A larger value of $l$ implies a faster decay of weight with increasing distance.

GCN is a geometric deep learning module frequently used in graph representation learning in recent years [2]. They effectively integrate information from neighbouring nodes to achieve efficient representation of target nodes. Subsequently, we employ encoders with two layers of GCNs to perform message passing and aggregation on pixel features and gene expression features, as shown in Eq.2:

$$\begin{aligned} \boldsymbol{E}^{\left( k \right)}={\tilde{\boldsymbol{D}}}^{-\frac{1}{2}}\tilde{\boldsymbol{A}}{\tilde{\boldsymbol{D}}}^{-\frac{1}{2}}\boldsymbol{E}^{\left( k-1 \right)}\boldsymbol{W}^{\left( k-1 \right)}\#\left( 2 \right) \end{aligned}$$

where $\boldsymbol{E}^{\left( k \right)}$ and $\boldsymbol{E}^{\left( k-1 \right)}$ represent the input and output of the GCN module, respectively. $\boldsymbol{E}^{(0)}$ corresponds to the pixel features $\boldsymbol{H}$ or gene expression features $\boldsymbol{G}$ for each spot. $\tilde{\boldsymbol{A}}=\boldsymbol{A}+\boldsymbol{I}$ denotes the adjacency matrix of the undirected graph, where $\boldsymbol{I}$ is the identity matrix. $\tilde{\boldsymbol{D}}$ and $\boldsymbol{W}^{\left( k-1 \right)}$ are the weighted degree matrix and trainable parameter respectively. The pixel features and gene expression features obtained after passing through the encoder are denoted as $\boldsymbol{E}_{H}$ and $\boldsymbol{E}_{G}$​.

#### 1.2.2 Feature fusion

To effectively aggregate multi-modal information, we propose a novel feature fusion strategy. First, stMMR uses a normalized attention module to learn the relationships between spots in a single modality, as shown in Eq.3:

$$\begin{aligned} \boldsymbol{E}_{A}=softmax\left( \frac{\boldsymbol{E}\cdot\boldsymbol{E}^{T}}{\sqrt{\boldsymbol{d}}} \right)\cdot\boldsymbol{E}\#\left( 3 \right) \end{aligned}$$

where $\boldsymbol{E}$ represents the pixel features $\boldsymbol{E}_{H}$ or gene expression features $\boldsymbol{E}_{G}$​ obtained in the previous step. The new features obtained through the attention module are $\boldsymbol{E}_{AH}$ and $\boldsymbol{E}_{AG}$​. It is noteworthy that we use a nonlinear activation function and the Euclidean distance matrix $\boldsymbol{d}$ to normalize the weights. This approach effectively avoids the issue of local optima caused by excessively large weights for certain spots [3].

For cross-modal information, stMMR adopts a contrastive learning approach for feature fusion. Previous research indicates that histology information and gene expression information share both similarities and complementary relationships [4–6]. stMMR emphasizes the consistency between multiple modalities by constructing a cross-modal contrastive learning strategy. Specifically, stMMR maps the latent features of multiple modalities, $\boldsymbol{E}_{H}$ and $\boldsymbol{E}_{G}$​, to a space using two fully connected neural networks, thereby obtaining hierarchical representations for both modalities, $\boldsymbol{Q}_{H}$​ and $\boldsymbol{Q}_{G}$​, as shown in Eq.4:

$$\begin{aligned} \boldsymbol{Q}=Relu\left( \boldsymbol{W}_{Q}\boldsymbol{E}+\boldsymbol{b}_{Q} \right)\boldsymbol{.\#}\left( 4 \right) \end{aligned}$$

In Eq.4, $\boldsymbol{E}$ represents the pixel features $\boldsymbol{E}_{H}$ or gene expression features $\boldsymbol{E}_{G}$​, and $\boldsymbol{Q}$ corresponds to $\boldsymbol{Q}_{H}$​ or $\boldsymbol{Q}_{G}$​. $\boldsymbol{W}_{Q}$ and $\boldsymbol{b}_{Q}$ are the parameters of the fully connected network.

After obtaining the low-dimensional features $\boldsymbol{Q}_{H}$​ and $\boldsymbol{Q}_{G}$ for the two modalities, we further employ a fully connected neural network to fuse these two modalities, as shown in Eq.5:

$$\begin{aligned} \boldsymbol{E}_{\boldsymbol{Q}}=\boldsymbol{W}_{E}\cdot concat\left( \boldsymbol{Q}_{G},\boldsymbol{Q}_{H} \right)+\boldsymbol{b}_{E}\#\left( 5 \right) \end{aligned}$$

where $\boldsymbol{W}_{E}$​ and $\boldsymbol{b}_{E}$​ are the parameters of the fully connected network.

To enhance the consistency between $\boldsymbol{Q}_{H}$​ and $\boldsymbol{Q}_{G}$​, we use a constraint as shown in Equation 6, replacing the loss of traditional contrastive learning:

$$\begin{aligned} L_{con}=\left\| \tilde{\boldsymbol{Q}_{G}}{\tilde{\boldsymbol{Q}_{G}}}^{T}-\tilde{\boldsymbol{Q}_{H}}{\tilde{\boldsymbol{Q}_{H}}}^{T} \right\|_{2}^{2}.\#\left( 6 \right) \end{aligned}$$

In this equation, $\tilde{\boldsymbol{Q}_{G}}$​ and $\tilde{\boldsymbol{Q}_{H}}$​ are the normalization matrices of $\boldsymbol{Q}_{G}$​ and $\boldsymbol{Q}_{H}$​, respectively.

Finally, we further integrate modality specific features $\boldsymbol{E}_{AH}$​ and $\boldsymbol{E}_{AG}$ obtained from Eq.3 with the cross-modality features $\boldsymbol{E}_{\boldsymbol{Q}}$ obtained from Eq.5 to get the multi-modal feature representation $\boldsymbol{Z}$, as shown in the following equation:

$$\begin{aligned} \boldsymbol{Z}={\alpha\boldsymbol{E}}_{\boldsymbol{Q}}+\beta\boldsymbol{E}_{AH}+\gamma\boldsymbol{E}_{AG}\boldsymbol{\#}\left( 7 \right) \end{aligned}$$

In this equation, $\alpha$, $\beta$, and $\gamma$ are hyperparameters for adjusting the importance of features.

#### 1.2.3 Feature reconstruction

Finally, to ensure that the embeddings $\boldsymbol{Z}$ learned by our model contain biologically information, we designed a reconstruction phase using $\boldsymbol{Z}$ to reconstruct the original features. Here, we primarily focus on reconstructing the adjacency matrix $\boldsymbol{A}$ and the gene expression profiles.

SRT data are characterized by high sparsity, discreteness, and variance greater than the mean, specifically manifested as a high number of genes expressed at zero (zero inflation) [7]. Previous research has found that the zero-inflated negative binomial (ZINB) distribution can effectively characterize gene expression in SRT [8]. Therefore, stMMR also adopts the ZINB to describe gene expression information. In simple terms, we estimate the parameters of the ZINB distribution for each gene (i.e., $\mu$, $\theta$, and $\pi$, Supplementary Section 1.4) through three different fully connected networks, as follows:

$$\boldsymbol{\Pi}=Sigmoid\left( {\boldsymbol{W}_{\pi}f}_{D}\left( \boldsymbol{Z} \right) \right),$$

$$\boldsymbol{\Theta}=exp(\boldsymbol{W}_{\theta}f_{D}\left( \boldsymbol{Z} \right)),$$

$$\boldsymbol{M}=exp(\boldsymbol{W}_{\mu}f_{D}\left( \boldsymbol{Z} \right)),$$

where $\mathbf{M}$, $\boldsymbol{\Theta}$, and $\boldsymbol{\Pi}$ are the matrix forms of $\mu$, $\theta$, and $\pi$, representing the mean, dispersion, and dropout probability of the output from network respectively. The dropout probability ranges between 0 and 1. Due to the non-negative nature of the mean $\mathbf{M}$ and dispersion $\boldsymbol{\Theta}$, we use the exponential function, and for the dropout probability $\boldsymbol{\Pi}$, the Sigmoid function is used. $f_{D}$ is a decoder with a fully connected layer.

Based on the aforementioned reconstruction of gene expression information, we further designed the loss function for the ZINB decoder, as shown below:

$$\begin{aligned} L_{ZINB}=-\log\left( ZINB\left( \boldsymbol{G}|\boldsymbol{M},\boldsymbol{\Theta},\boldsymbol{\Pi} \right) \right)\#\left( 8 \right) \end{aligned}$$

For spatial neighborhood relationships, we adopted the concept of a graph auto-encoder to directly estimate the adjacency matrix [9,10], as shown in Eq.9:

$$\begin{aligned} \boldsymbol{A}^{'}=Sigmoid\left( \frac{\boldsymbol{Z}\cdot\boldsymbol{Z}^{\boldsymbol{T}}}{\left\| \boldsymbol{Z} \right\|_{2}\cdot\left\| \boldsymbol{Z}^{\boldsymbol{T}} \right\|_{2}} \right)\#\left( 9 \right) \end{aligned}$$

Subsequently, we defined a function to calculate the regularization loss between the reconstructed matrix and the adjacency matrix, as in Eq.10:

$$\begin{aligned} L_{rec}=\frac{1}{N^{2}}\sum_{i=1}^{N} \sum_{j=1}^{N} \left( \boldsymbol{A}_{ij}-\boldsymbol{A}_{ij}^{'} \right)^{2}\#\left( 10 \right) \end{aligned}$$

This function quantifies the difference between the original adjacency matrix $\boldsymbol{A}$ and its reconstructed version $\boldsymbol{A}^{\boldsymbol{'}}$, thereby facilitating the accurate reconstruction of spatial relationships.

#### 1.2.4 Final Objective function

Finally, we integrated Eq.6, Eq.8, and Eq.10 to formulate the final objective function, as shown in Eq.11:

$$\begin{aligned} {L=a*L}_{con}+b*L_{ZINB}+c*L_{rec}\#\left( 11 \right) \end{aligned}$$

In this equation, $a$, $b$, and $c$ are hyperparameters that control the contribution of the different loss terms.

For detailed information on the training process and parameter settings, please refer to the Supplementary Section 1.5 and 1.6.

## 1.3 Pre-trained ViT Model

The morphological features of each spot are extracted using a pre-trained Vision Transformer (ViT) model [1], which has demonstrated SOTA performance and excellent transferability across various datasets. Specifically, for each tissue section in ST data, we begin by accurately extracting the image of each spot based on its spatial coordinates and size. Each spot image is then subdivided into 4x4 patches. The ViT model deeply learns from these patches, effectively capturing the visual characteristics of each spot. This process ultimately transforms each spot image into a representation composed of 16,000 latent variables. To more accurately represent the morphological features of the spots and balance the weights between expression and histological information, we employ PCA to extract the top 3,000 principal components (PCs) as the final latent features.

## 1.4 ZINB model for gene expression

We integrate the ZINB model to facilitate the reconstruction of gene expression information from the latent representation $Z$. Following this, we will conduct a brief illustration on the rationale behind the utilization of the ZINB distribution in previous studies to approximate the distribution of ST data. For a more comprehensive introduction, please refer to [8].

In the SRT data, the distribution of gene expression matrices typically exhibits three characteristics: 1) discreteness; 2) variance exceeding the mean; and 3) matrix sparsity, including non-expressed genes (true zeros) or those unexpressed due to technical reasons (dropout zeros). ZINB model effectively fits the three distinct properties of SRT data.

The definition of the ZINB model is as follows:

$$f_{ZINB}\left( g|\pi,r,p \right)=\pi I_{O}\left( G \right)+\left( 1-\pi\right)f_{NB}\left( g|r,p \right)$$

$$f_{NB}\left( g|r,p \right)=\left( \begin{matrix} g+r-1 \\ g \end{matrix} \right)p^{r}\left( 1-p \right)^{g}$$

In this model, $\pi$ represents the proportion of zero values, and $I_{O}$ is an indicator function, which is 0 when there is gene expression and 1 otherwise. The parameters $r$ and $p$ are associated with the Negative Binomial distribution, representing the number of successes and the probability, respectively.

Given that the NB distribution is a discrete distribution, the ZINB distribution also adheres to the properties of a discrete distribution. Specifically, when $g=0$, the ZINB model can predict the probability of dropout being zero (dropout rate) through $\pi$. This can be derived as follows:

$$d=\frac{\left( 1-\pi\right)f_{NB}\left( 0 \right)}{\pi+\left( 1-\pi\right)f_{NB}\left( 0 \right)}.$$

Further, assuming the mean is E(g), it is defined as follows:

$$E\left( g \right)=\sum_{g=0}^{\infty} g\left( \begin{matrix} g+r-1 \\ g \end{matrix} \right)p^{r}\left( 1-p \right)^{g}.$$

Let $g^{'}=g+1$ and $r^{'}=r+1$,

$$E\left( g \right)=\frac{r\left( 1-p \right)}{p}\sum_{g^{'}=0}^{\infty} f_{NB}\left( g'|r',p \right).$$

Since the Negative Binomial (NB) distribution is a discrete distribution, the sum of all probabilities within this distribution equals 1.

$$\sum_{g^{'}=0}^{\infty} f_{NB}\left( g'|r',p \right)=1.$$

Therefore,

$$E\left( g \right)=\frac{r\left( 1-p \right)}{p}.$$

Assuming that the variance can be defined as follows:

$$Var\left( g \right)= E\left( g^{2} \right)-E\left( g \right)^{2}=\frac{r\left( 1-p \right)}{p^{2}}.$$

We can get the relationships between $E\left( g \right)$ and $Var\left( g \right)$:

$$Var\left( g \right)=\frac{E\left( g \right)^{2}}{r}$$

where $r>0$ and $Var\left( g \right)>E\left( g \right)$.

Building upon this foundation, ZINB model is used to simulate the distribution of ST data, with the aim of capturing the unique characteristics inherent to ST data.

$$\mathrm{NB}\left( G;\mu,\theta\right)=\frac{\Gamma\left( G+\theta\right)}{G!\Gamma\left( \theta\right)}\left( \frac{\theta}{\theta+\mu} \right)^{\theta}\left( \frac{\mu}{\theta+\mu} \right)^{G},$$

$$\mathrm{ZINB}\left( G;\mu,\theta,\pi\right)=\pi\delta_{0}\left( G \right)+\left( 1-\pi\right)\mathrm{NB}\left( g;\mu,\theta\right)$$

where $G$ represents spatial transcriptomic data, $\delta_{0}$ denotes the Dirac function, and $\pi$ is the probability of the true gene expression values being observed as zero ($p=\frac{\theta}{\theta+\mu}$).

## 1.5 Hyperparameters and training strategy

It is widely acknowledged that the generalization performance of a model is a critical metric for assessing its efficacy [11]. Different SRT datasets may possess distinct characteristics, such as feature distribution, noise levels, and sample sizes. Adjustments in hyperparameters could optimize a model's performance on a specific dataset, but might concurrently compromise its generalization capability. Furthermore, some models may be extremely sensitive to hyperparameter tuning, where minor adjustments can lead to significant performance fluctuations. In such cases, the actual performance of a model is largely dependent on meticulous hyperparameter tuning.

To demonstrate the superior performance of stMMR, we endeavor to maintain consistency in hyperparameters across all datasets. For hyperparameters intrinsic to the model discussed in this paper, the following settings were applied. For all datasets, each spot was ultimately embedded into a 1*3000 vector. In all datasets, the $\alpha$, $\beta$, and $\gamma$ in the main text's equation 7 were set to 10, 1, and 20, respectively. Similarly, in equation 11, the $a$, $b$, and $c$ were set to 1, 10, and 10 across all datasets. For the parameter $l$ in equation 1, adjustments were made due to varying sizes of experimental data spots and their distances, setting $l$ to 2 for the DLPFC, chicken heart, and human lung cancer datasets, and to 0.67 for the human breast cancer dataset.

For the parameters used during the training of the model, we also maintained consistency across all datasets. We employed the Adam optimizer, with the learning rate set to 0.0001 and weight_decay also configured to 0.0001. The model training continued until the loss converged.

## 1.6 Platform

We employed two different hardware setups for testing stMMR. The first setup operated on Ubuntu 20.04, equipped with an Intel(R) Xeon(R) Gold 6258R CPU and an NVIDIA Quadro GV100 GPU. The second setup ran on a Windows 10 operating system, featuring an AMD Ryzen 7 2700X CPU and an NVIDIA GeForce RTX 3090 GPU.

# Datasets and Data processing

## 2.1 Datasets

In our study, we utilized datasets with paired gene expression and histology data, which are publicly available from 10x Genomics and NanoString websites. Processed datasets are also available at SODB and can be loaded by PySODB [12,13].

1. **Dorsolateral Prefrontal Cortex (DLPFC)**: The 10x Visium DLPFC dataset contains 12 tissue sections, manually annotated for six cortical layers and White Matter (WM), with the number of spots ranging from 3460 to 4789. This dataset is accessible at [DLPFC 10x Visium dataset](http://spatial.libd.org/spatialLIBD/) [14].
2. **Chicken Heart**: The 10x Visium dataset for chicken heart spans 12 tissue slices, annotated across four key Hamburger-Hamilton ventricular developmental stages. Sequencing was performed on the 4th day (5 slices), 7th day (4 slices), 10th day (2 slices), and 14th day (1 slice). The dataset can be found at [Chicken heart 10x Visium dataset](https://www.ncbi.nlm.nih.gov/geo/query/acc.cgi?acc=GSE149457) [15].
3. **Human Breast Cancer**: The 10x Visium dataset for human breast cancer comprises 3798 spots and 36,601 genes, with 20 regions manually annotated. It is available at [Human breast cancer 10x Visium dataset](https://support.10xgenomics.com/spatial-gene-expression/datasets/1.1.0/V1_Breast_Cancer_Block_A_Section_1).
4. **Lung Cancer**: Generated by NanoString CosMx SMI, the lung cancer dataset consists of 20 Fields of View (FOVs), encompassing 980 genes and 91,992 cells, covering eight primary cell types. This dataset can be accessed at [Lung cancer (9-1) nanostring dataset](https://nanostring.com/products/cosmx-spatial-molecular-imager/nsclc-ffpe-dataset/) [16].
5. **Human Pancreatic Ductal Adenocarcinoma (PDAC)**: The ST PDAC dataset contains 428 spots and 19,738 genes, with 4 regions manually annotated. This dataset can be accessed at [Human pancreatic ductal adenocarcinoma ST dataset](https://www.ncbi.nlm.nih.gov/geo/query/acc.cgi?acc=GSM3036911).
6. **Mouse Brain**: 10x Visium dataset for mouse brain comprises 2695 spots and 32,285 genes, with 52 regions manually annotated. It is available at [Mouse brain 10x Visium dataset](https://www.ncbi.nlm.nih.gov/geo/query/acc.cgi?acc=GSM3036911).
7. **Human Colorectal Cancer (CRC)**: The 10x Visium HD CRC dataset contains 12,319 spots and 18,085 genes. This dataset is accessible at [Human CRC Visium HD dataset](https://www.10xgenomics.com/products/visium-hd-spatial-gene-expression/dataset-human-crc).

For more detailed information, please see Supplementary Table S1.

## 2.2 Data processing

For all datasets used in our study, we initially removed spots located outside the main tissue regions. Subsequently, the raw gene expression counts were log-transformed and normalized for library size using the SCANPY package [17], scaling the normalized gene expression counts to unit variance and zero mean. We selected the top 3000 highly variable genes (HVGs) to serve as the input for stMMR.

## 2.3 Domain-specific gene analysis

Domain-specific genes exhibit unique expression patterns across different spatial domains. To identify these domain-specific genes, we performed differential expression analysis using the Wilcoxon test within the SCANPY package [17]. Genes meeting a 1% false discovery rate threshold were identified as domain-specific genes.

For the DLPFC dataset, we conducted tests to assess gene expression differences in each domain, both on the original gene expression data and on augmented gene expression data. In the human lung cancer dataset, we randomly selected Fov10 to exam the differential expression of genes across eight cell types. In the human breast cancer dataset, we merged the healthy and tumor margin areas to explore the domain-specific gene expression pattern between these regions.

# Reference

1. Dosovitskiy A, Beyer L, Kolesnikov A, et al. An Image is Worth 16x16 Words: Transformers for Image Recognition at Scale. 2021;

2. Kipf TN, Welling M. Semi-Supervised Classification with Graph Convolutional Networks. 2016;

3. Brauwers G, Frasincar F. A General Survey on Attention Mechanisms in Deep Learning. IEEE Trans. Knowl. Data Eng. 2023; 35:3279–3298

4. Markey M, Kim J, Goldstein Z, et al. Abstract B010: Spatially-resolved prediction of gene expression signatures in H&E whole slide images using additive multiple instance learning models. Molecular Cancer Therapeutics 2023; 22:B010–B010

5. Bergenstråhle L, He B, Bergenstråhle J, et al. Super-resolved spatial transcriptomics by deep data fusion. Nat Biotechnol 2022; 40:476–479

6. Zeng Y, Wei Z, Yu W, et al. Spatial transcriptomics prediction from histology jointly through Transformer and graph neural networks. Briefings in Bioinformatics 2022; 23:bbac297

7. Covert I, Gala R, Wang T, et al. Predictive and robust gene selection for spatial transcriptomics. Nat Commun 2023; 14:2091

8. Yu Z, Lu Y, Wang Y, et al. ZINB-Based Graph Embedding Autoencoder for Single-Cell RNA-Seq Interpretations. Proceedings of the AAAI Conference on Artificial Intelligence 2022; 36:4671–4679

9. Kipf TN, Welling M. Variational Graph Auto-Encoders. 2016;

10. Tang M, Yang C, Li P. Graph Auto-Encoder Via Neighborhood Wasserstein Reconstruction. 2022;

11. Yang L, Shami A. On hyperparameter optimization of machine learning algorithms: Theory and practice. Neurocomputing 2020; 415:295–316

12. Yuan Z, Pan W, Zhao X, et al. SODB facilitates comprehensive exploration of spatial omics data. Nat Methods 2023; 20:387–399

13. Lin S, Zhao F, Wu Z, et al. Streamlining spatial omics data analysis with Pysodb. Nat Protoc 2023; 1–65

14. Maynard KR, Collado-Torres L, Weber LM, et al. Transcriptome-scale spatial gene expression in the human dorsolateral prefrontal cortex. Nat Neurosci 2021; 24:425–436

15. Mantri M, Scuderi GJ, Abedini-Nassab R, et al. Spatiotemporal single-cell RNA sequencing of developing chicken hearts identifies interplay between cellular differentiation and morphogenesis. Nat Commun 2021; 12:1771

16. He S, Bhatt R, Brown C, et al. High-plex imaging of RNA and proteins at subcellular resolution in fixed tissue by spatial molecular imaging. Nat Biotechnol 2022; 40:1794–1806

17. Wolf FA, Angerer P, Theis FJ. SCANPY: large-scale single-cell gene expression data analysis. Genome Biology 2018; 19:15
