# Supplementary material for: stMMR: accurate and robust spatial domain identification from spatially resolved transcriptomics with multimodal feature representation
Source: Gigascience. 2024 Nov 28;13:giae089. doi: 10.1093/gigascience/giae089 (PMC11604062; doi:10.1093/gigascience/giae089)
Supplement: giae089_Supplementary_Files [file giae089_supplementary_files.zip › Supplementary_Table_S1.docx]

**Supplementary Table S1**. Summary of the datasets used in this study.

| Platform | Tissue | Section | #Spots | #Genes | Categories |
| --- | --- | --- | --- | --- | --- |
| 10x Visium | Human dorsolateral prefrontal cortex (DLPFC) | 151507,  151508,  151509,  151510,  151669,  151670,  151671,  151672,  151673,  151674,  151675,  151676 | 4226,  4384,  4789,  4634  3661,  3498,  4110,  4015,  3639,  3673,  3592,  3460 | 33538,  33538,  33538,  33538,  33538,  33538,  33538,  33538,  33538,  33538,  33538,  33538 | 7,  7,  7,  7,  5,  5,  5,  5,  7,  7,  7,  7 |
|  | Human breast cancer | Human breast cancer  section 1 | 3798 | 36601 | 20 |
|  | Chicken heart | D4,  D7,  D10,  D14 | 747,  1966,  1916,  1967 | 24356,  24356,  24356,  24356 | 5,  7,  7,  6 |
|  | Mouse_brain | Sagittal_anterior_  section_1 | 2695 | 32285 | 52 |
| 10x Visium HD | Human colorectal cancer (CRC) | P2 CRC | 12319 | 18085 | NA |
| NanoString CosMx SMI | Human non-small cell lung cancer | Lung9-1 | 91992 | 980 | 8 |
| ST | Human pancreatic ductal adenocarcinoma | PDAC-A ST1 | 428 | 19738 | 4 |
