# Supplementary figures and images for: stMMR: accurate and robust spatial domain identification from spatially resolved transcriptomics with multimodal feature representation
Source: Gigascience. 2024 Nov 28;13:giae089. doi: 10.1093/gigascience/giae089 (PMC11604062; doi:10.1093/gigascience/giae089)

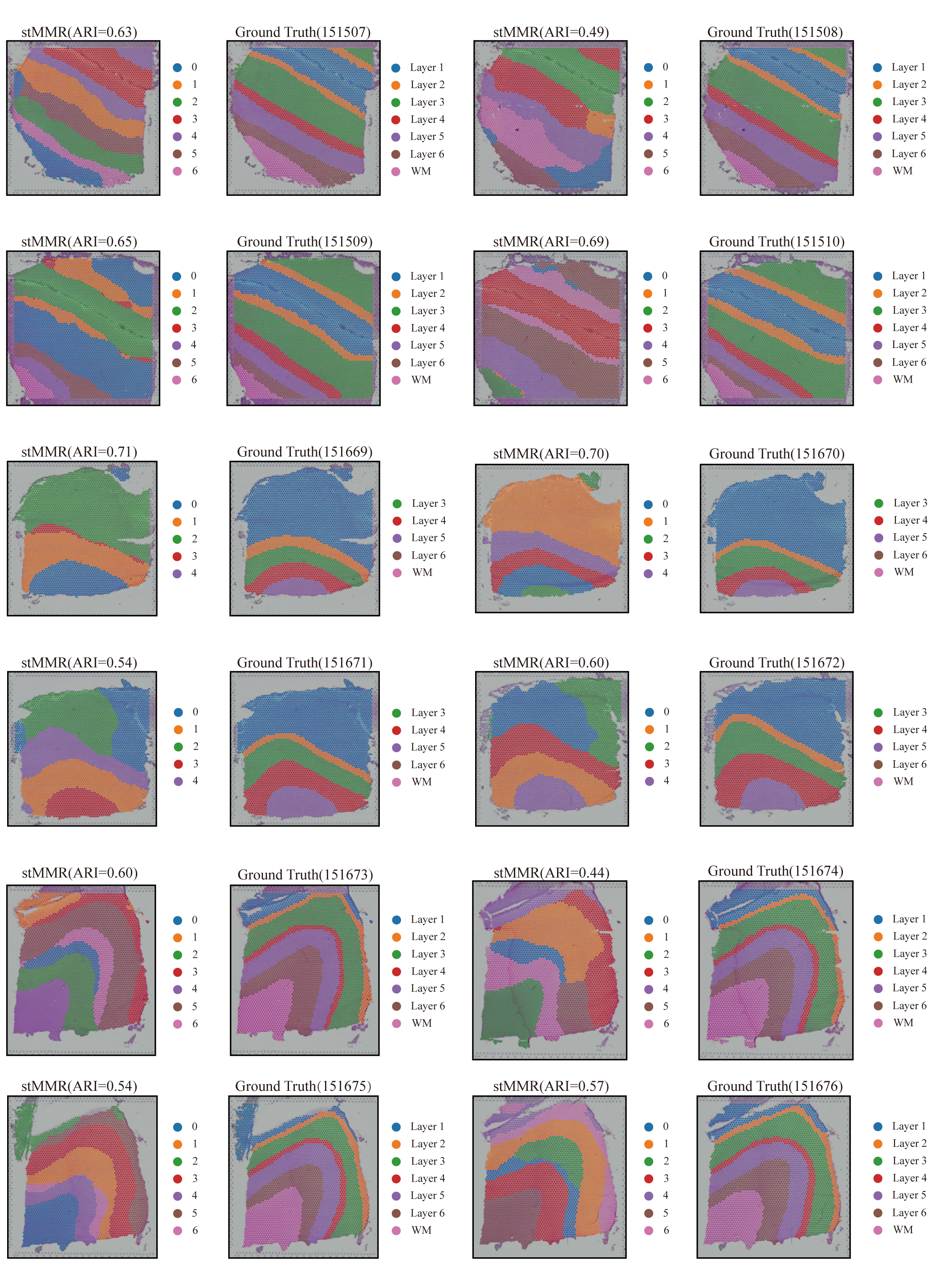

Supplement: giae089_Supplementary_Files [file giae089_supplementary_files.zip › Supplementary_Figure_S1.jpg]

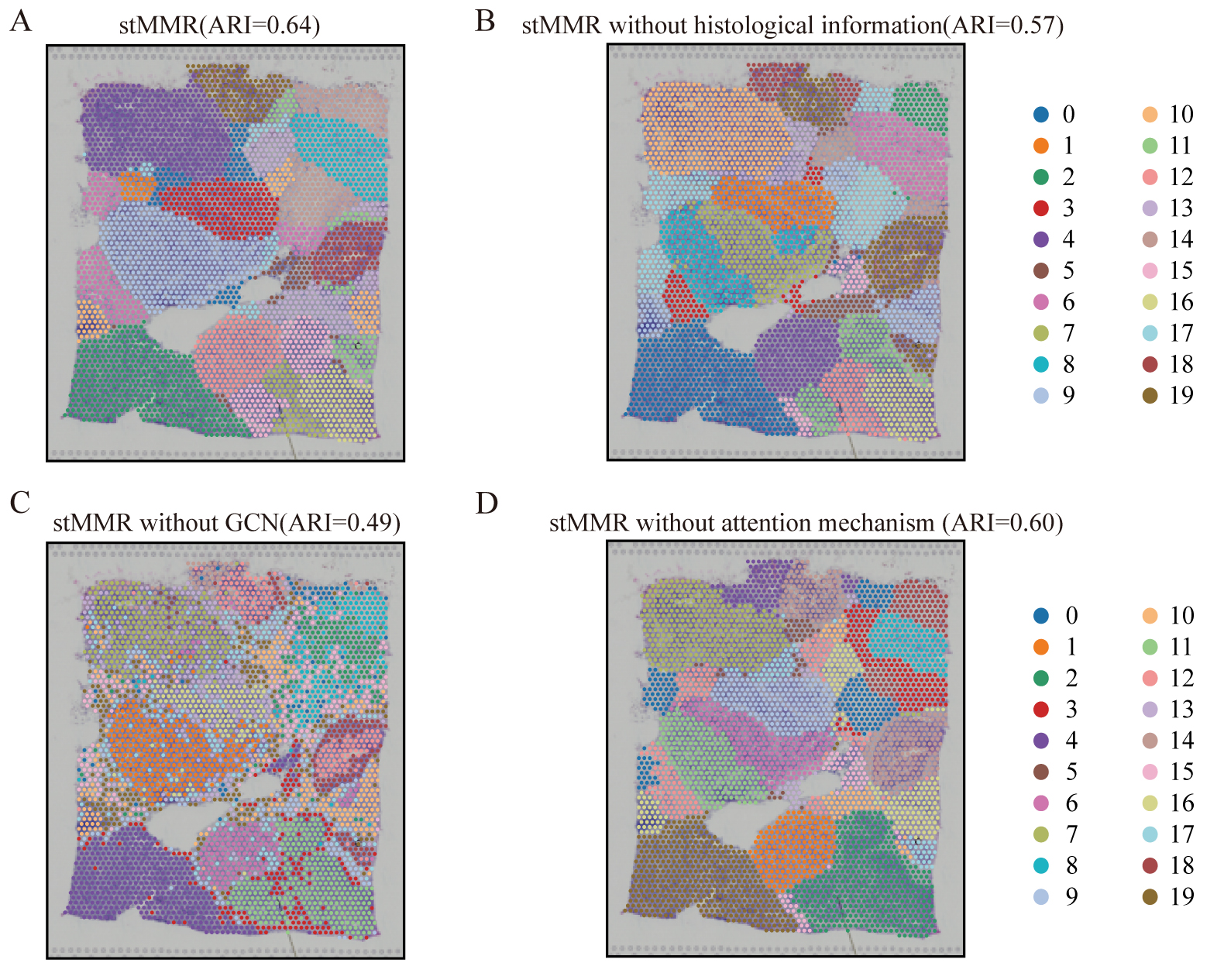

Supplement: giae089_Supplementary_Files [file giae089_supplementary_files.zip › Supplementary_Figure_S2.jpg]

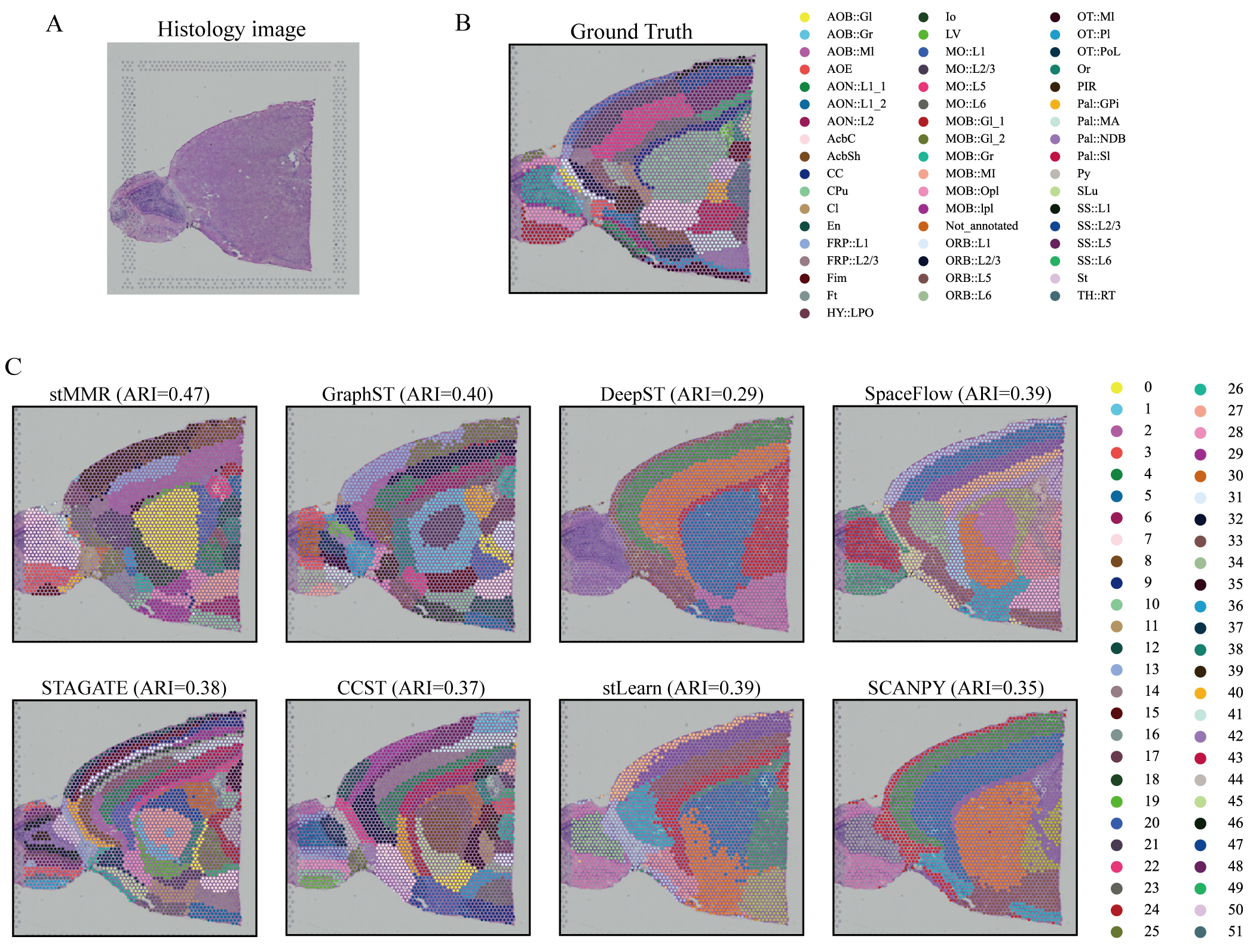

Supplement: giae089_Supplementary_Files [file giae089_supplementary_files.zip › Supplementary_Figure_S3.jpg]

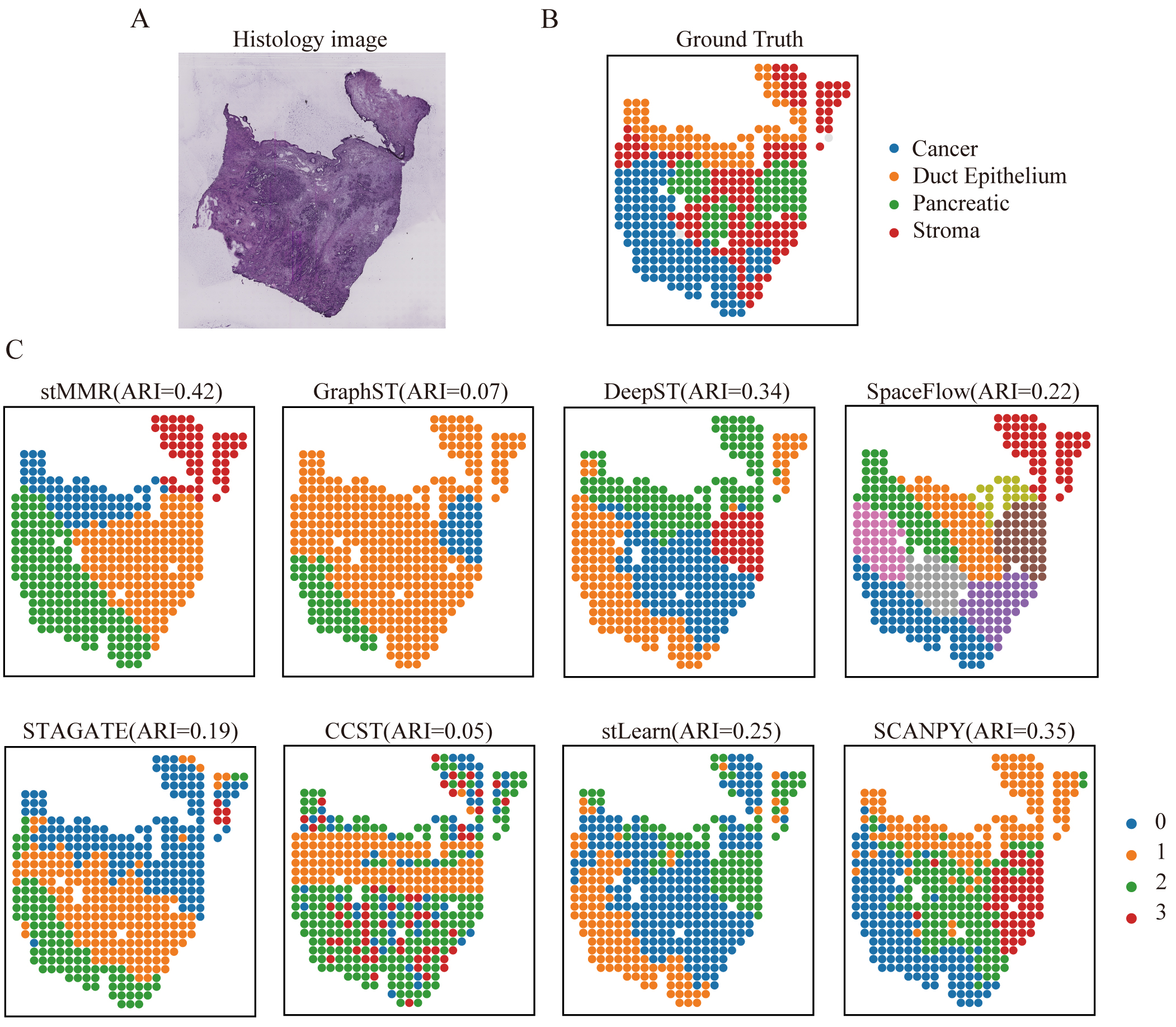

Supplement: giae089_Supplementary_Files [file giae089_supplementary_files.zip › Supplementary_Figure_S4.jpg]

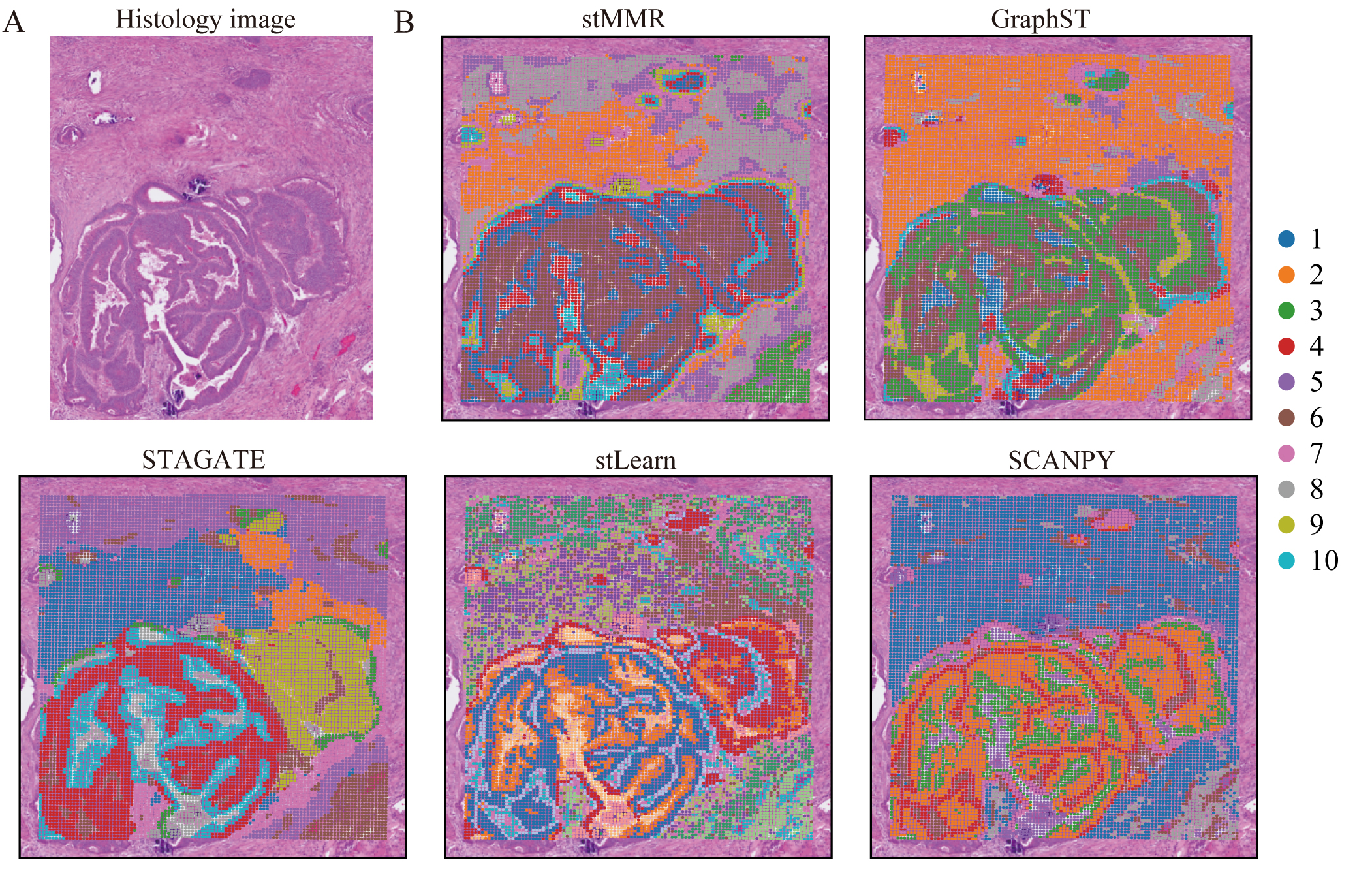

Supplement: giae089_Supplementary_Files [file giae089_supplementary_files.zip › Supplementary_Figure_S5.jpg]

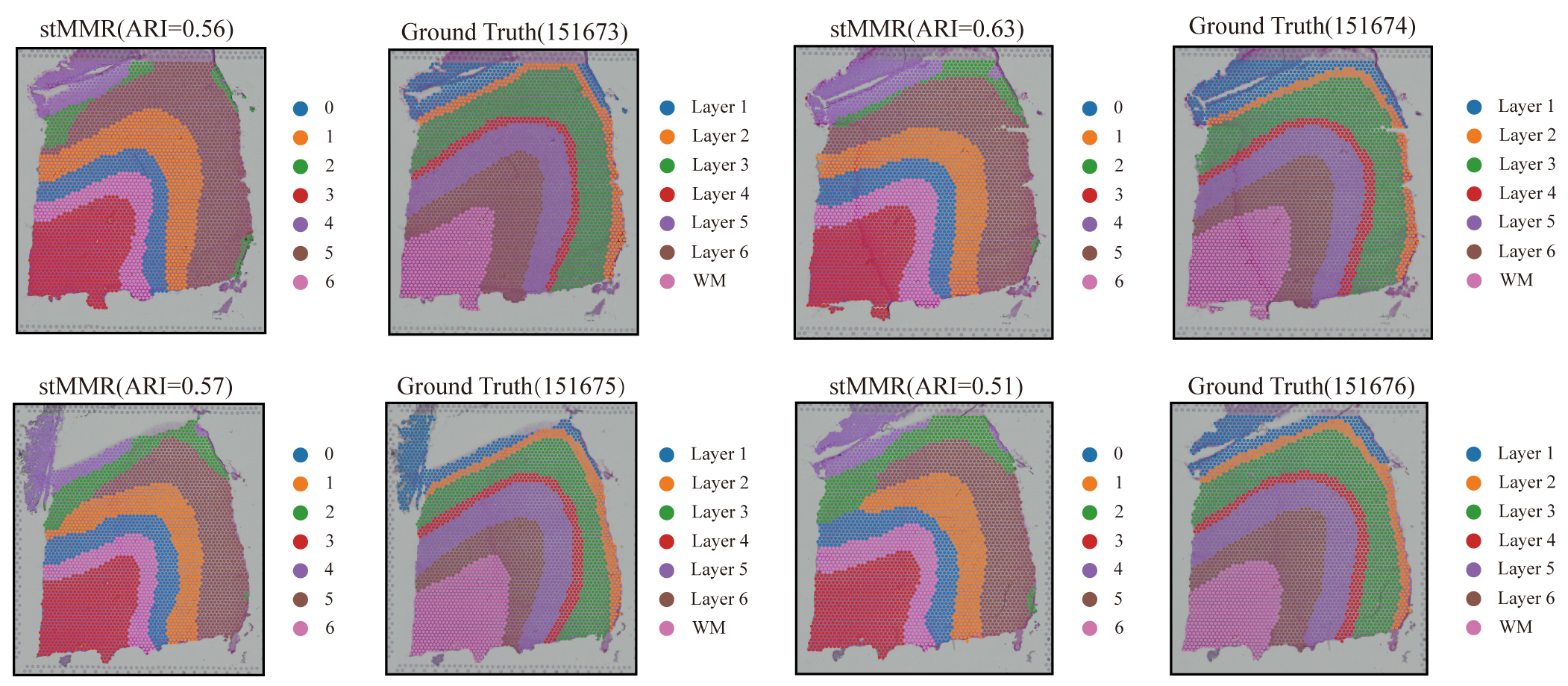

Supplement: giae089_Supplementary_Files [file giae089_supplementary_files.zip › Supplementary_Figure_S6.jpg]
