# Supplementary material for: stMMR: accurate and robust spatial domain identification from spatially resolved transcriptomics with multimodal feature representation
Source: Gigascience. 2024 Nov 28;13:giae089. doi: 10.1093/gigascience/giae089 (PMC11604062; doi:10.1093/gigascience/giae089)
Supplement: giae089_GIGA-D-24-00153_Revision_1 [file giae089_giga-d-24-00153_revision_1.pdf]

GigaScience

stMMR: accurate and robust spatial domain identification from spatially resolved transcriptomics with multi-modal feature representation

--Manuscript Draft--

**Manuscript Number:**	GIGA-D-24-00153R1
**Full Title:**	stMMR: accurate and robust spatial domain identification from spatially resolved transcriptomics with multi-modal feature representation
**Article Type:**	Technical Note
**Funding Information:**	National Natural Science Foundation of China (62303271) — Dr. Wei Zhang<br>National Natural Science Foundation of China (U1806202) — Prof Rui Gao<br>National Natural Science Foundation of China (62373216) — Prof Zhiping Liu<br>National Natural Science Foundation of China (92374107) — Prof Zhiping Liu<br>Natural Science Foundation of Shandong Province (ZR2023QF081) — Dr. Wei Zhang
**Abstract:**	Background: Deciphering spatial domains using spatially resolved transcriptomics (SRT) is of great value for the characterizing and understanding of tissue architecture. However, the inherent heterogeneity and varying spatial resolutions present challenges in the joint analysis of multi-modal SRT data.<br>Results: We introduce a multi-modal geometric deep learning method, named stMMR, to effectively integrate gene expression, spatial location and histological information for accurate identifying spatial domains from SRT data. stMMR uses graph convolutional networks (GCN) and self-attention module for deep embedding of features within unimodal and incorporates similarity contrastive learning for integrating features across modalities.<br>Conclusions: Comprehensive benchmark analysis on various types of spatial data shows superior performance of stMMR in multiple analyses, including spatial domain identification, pseudo-spatiotemporal analysis and domain-specific gene discovery. In chicken heart development, stMMR reconstruct the spatiotemporal lineage structures indicating accurate developmental sequence. In breast cancer and lung cancer, stMMR clearly delineated the tumor microenvironment and identified marker genes associated with diagnosis and prognosis. Overall, stMMR is capable of effectively utilizing the multi-modal information of various SRT data to explore and characterize tissue architectures of homeostasis, development and tumor.
**Corresponding Author:**	Wei Zhang<br>Shandong University<br>Jinan, CHINA
**Corresponding Author Secondary Information:**	
**Corresponding Author's Institution:**	Shandong University
**Corresponding Author's Secondary Institution:**	
**First Author:**	Daoliang Zhang
**First Author Secondary Information:**	
**Order of Authors:**	Daoliang Zhang<br>Na Yu

Powered by Editorial Manager® and ProduXion Manager® from Aries Systems Corporation

	Zhiyuan Yuan
	Wenrui Li
	Xue Sun
	Qi Zou
	Xiangyu Li
	Zhiping Liu
	Wei Zhang
	Rui Gao
**Order of Authors Secondary Information:**	
**Response to Reviewers:**	Manuscript ID: GIGA-D-24-00153<br>Title: stMMR: accurate and robust spatial domain identification from spatially resolved transcriptomics with multi-modal feature representation<br><br>Dear Reviewers,<br><br>We sincerely thank you for the great efforts to deal with our manuscript as well as the constructive and thoughtful feedback. These comments encouraged us to substantially improve the quality of our work. According to your comments and suggestions, we have revised our manuscript and source code thoroughly.<br><br>Please find attached a revised version of our manuscript and detailed responses to the reviewers' comments (as a separate PDF file titled “Response Letter.pdf” as Supplemental Material).<br><br>Thank you once again for the valuable feedback on our work.<br><br>Wei Zhang et al.<br>School of Control Science and Engineering<br>Shandong University<br>Jinan 250061, P.R. China
**Additional Information:**	
**Question**	**Response**
Are you submitting this manuscript to a special series or article collection?	No
**Experimental design and statistics**<br><br>Full details of the experimental design and statistical methods used should be given in the Methods section, as detailed in our Minimum Standards Reporting Checklist. Information essential to interpreting the data presented should be made available in the figure legends.<br><br>Have you included all the information requested in your manuscript?	Yes
**Resources**	No

Powered by Editorial Manager® and ProduXion Manager® from Aries Systems Corporation

A description of all resources used, including antibodies, cell lines, animals and software tools, with enough information to allow them to be uniquely identified, should be included in the Methods section. Authors are strongly encouraged to cite Research Resource Identifiers (RRIDs) for antibodies, model organisms and tools, where possible.<br><br>Have you included the information requested as detailed in our Minimum Standards Reporting Checklist?	
If not, please give reasons for any omissions below.<br><br>as follow-up to "**Resources**<br><br>A description of all resources used, including antibodies, cell lines, animals and software tools, with enough information to allow them to be uniquely identified, should be included in the Methods section. Authors are strongly encouraged to cite Research Resource Identifiers (RRIDs) for antibodies, model organisms and tools, where possible.<br><br>Have you included the information requested as detailed in our Minimum Standards Reporting Checklist?<br><br>"	Not applicable.
**Availability of data and materials**<br><br>All datasets and code on which the conclusions of the paper rely must be either included in your submission or deposited in publicly available repositories (where available and ethically appropriate), referencing such data using a unique identifier in the references and in the “Availability of Data and Materials”	Yes

section of your manuscript.<br><br>Have you have met the above requirement as detailed in our Minimum Standards Reporting Checklist?	

Click here to access/download;LaTeX - Figure (eps, ps, etc.);example-grid-100x100pt.png

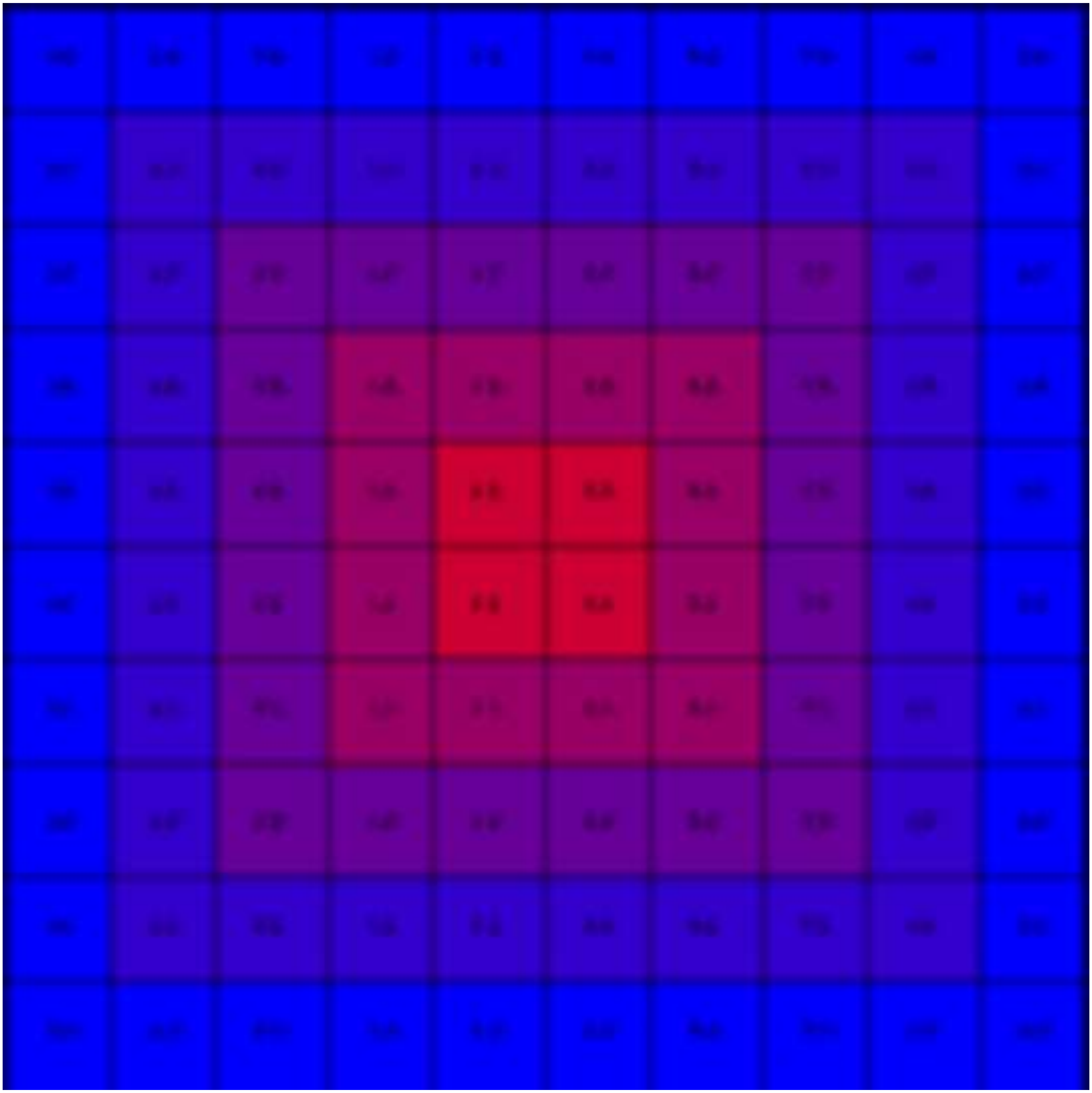

main text
Click here to access/download;Manuscript;stMMR.pdf

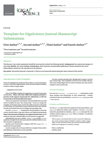

GigaScience, 2023, 1–13

doi: xx.xxxx/xxxx
Manuscript in Preparation
Technical Note

TECHNICAL NOTE

stMMR: accurate and robust spatial domain identification from spatially resolved transcriptomics with multi-modal feature representation

Daoliang Zhang[1,†], Na Yu[1,†], Zhiyuan Yuan[2,†], Wenrui Li[3], Xue Sun[1], Qi Zou[1], Xiangyu Li[4], Zhiping Liu[1], Wei Zhang[1,*] and Rui Gao[1,*]

[1]Center of Intelligent Medicine, School of Control Science and Engineering, Shandong University, Jinan 250061, China and [2]Institute of Science and Technology for Brain-Inspired Intelligence, Center for Medical Research and Innovation, Shanghai Pudong Hospital, Fudan University Pudong Medical Center, Fudan University, Shanghai 200433, China and [3]MOE Key Lab of Bioinformatics and Bioinformatics Division of BNRIST, Department of Automation, Tsinghua University, Beijing 100084, China and [4]School of Software Engineering, Beijing Jiaotong University, Beijing 100044, China

[*] Correspondence address. Rui Gao, No. 17923, Jingshi Road, Lixia District, Jinan, Shandong, China. E-mail: gaorui@sdu.edu.cn; Wei Zhang, No. 17923, Jingshi Road, Lixia District, Jinan, Shandong, China. E-mail: zw@sdu.edu.cn
[†] These authors contributed equally as the first authors.

Abstract

Background: Deciphering spatial domains using spatially resolved transcriptomics (SRT) is of great value for the characterizing and understanding of tissue architecture. However, the inherent heterogeneity and varying spatial resolutions present challenges in the joint analysis of multi-modal SRT data. **Results:** We introduce a multi-modal geometric deep learning method, named stMMR, to effectively integrate gene expression, spatial location and histological information for accurate identifying spatial domains from SRT data. stMMR uses graph convolutional networks (GCN) and self-attention module for deep embedding of features within unimodal and incorporates similarity contrastive learning for integrating features across modalities. **Conclusions:** Comprehensive benchmark analysis on various types of spatial data shows superior performance of stMMR in multiple analyses, including spatial domain identification, pseudo-spatiotemporal analysis and domain-specific gene discovery. In chicken heart development, stMMR reconstruct the spatiotemporal lineage structures indicating accurate developmental sequence. In breast cancer and lung cancer, stMMR clearly delineated the tumor microenvironment and identified marker genes associated with diagnosis and prognosis. Overall, stMMR is capable of effectively utilizing the multi-modal information of various SRT data to explore and characterize tissue architectures of homeostasis, development and tumor.

Key words: spatially resolved transcriptomics; domain identification; multi-modal integration; geometric deep learning; similarity contrastive learning

Introduction

The advancement in spatially resolved transcriptomics (SRT) technologies has opened new avenues for a deeper understanding of the spatial architecture and functionality of tissues. Currently, many SRT technologies have been developed, such as imaging-based and sequencing-based methods [1, 2, 3, 4, 5, 6, 7]. Among these, techniques such as 10x Genomics Visium not only provide the spatial

Compiled on: October 21, 2024.
Draft manuscript prepared by the author.

location and gene expression data for each spot but also acquire high-resolution hematoxylin and eosin (H&E) stained histology images of the tissue section, revealing richer information about the tissue organization. These technological advancements offer new insights into characterization of tissue architecture, enabling a more comprehensive understanding of tissue development and disease pathogenesis [7, 8, 9].

For SRT technologies capable of providing both gene expression data and histology images, the information from these different modalities reflects the structural information of tissues at various levels. Gene expression profiles reflect the difference of cell state between spots [10]. Spatial location information provides the precise location of each spot. Histological images display morphological features of cells, such as size and shape [11]. Although each of these modalities has its own strength, they complement each other, together forming a more comprehensive picture of tissue architecture. For instance, changes in gene expression are reflected not only at the molecular level but may also manifest in histological images as morphological alterations[12]. Furthermore, the issues of sparsity and dropout in SRT data can be effectively addressed through integrating histological image data [13]. By leveraging the interdependence between gene expression and morphological features, as well as the similarity in gene expression patterns among adjacent spots, we can enhance spatial signals and characterize tissue structure.

However, the joint representation of multi-modal features in SRT is challenging. Firstly, these different modalities inherently possess significant heterogeneity. For instance, transcriptomic data are typically high-dimensional, quantified gene expression information, reflecting the gene activity in different spots or cells. In contrast, histology images are two-dimensional visual data depicting the morphological and structural information of cells at different spots. This fundamental difference makes the direct fusion of these two types of modalities difficult. Secondly, the disparity in data scale and resolution is also a crucial issue. Transcriptomic data reveals unique patterns of gene expression within spots or cells from a microscopic perspective. Conversely, histology images provide more macroscopic information on organization and morphology. This difference in scale complicates the establishment of spatial correspondence. Therefore, there is an urgent need for methods that can effectively integrate multi-modal features.

Recently, a variety of cutting-edge computational methods have been developed to effectively address the challenge of joint representation of multi-modal SRT data. Specifically, BASS, BayesSpace and Giotto leverage spatial neighborhood information for enhancing the resolution of SRT data [14, 15, 16]. CellCharter and PRECAST incorporate spatial contexts to correct batch effect for a better domain identification [17, 18]. MENDER is a recently proposed multi-range cell context decipherer for ultra-fast tissue structure identification [19]. CCST, STAGATE, SpaceFlow and GraphST utilize Graph Neural Networks (GNN) to integrate gene expression data with spatial information, achieving effective clustering of spots [20, 21, 22, 23]. However, these methods do not employ histology images, failing to fully enhance the interpretability of gene expression data through these images. In contrast, recent pioneering studies like stLearn and DeepST have shown more significant progress [24, 25]. These methods effectively integrate gene expression data with spatial neighborhood information and morphological features extracted from histology images, demonstrating a stronger potential for application. Despite these methods demonstrating capability in processing multi-modal information in SRT data, they give less consideration to the complex global spot similarity across distinctive spatial multi-modal features. This limitation impedes their ability to accurately characterize spatial patterns and discover functional biological contexts in tissue.

To achieve precise identification of spatial domains, we introduce stMMR, a geometric deep learning method for effective representing multi-modal information in SRT data. stMMR utilizes spatial location information as a bridge to establish adjacency relationships between spots. It encodes gene expression data and morphological features extracted from histological images using GCN. stMMR proposed a novel strategy to achieve joint learning of intra-modal and inter-modal features. Within a certain modality, stMMR employs self-attention mechanisms to dynamically learn the complex relationships of different spots. For integrating cross-modal information, stMMR innovatively utilizes similarity contrastive learning along with the reconstruction of gene expression features and adjacency information. This enhances the ability of stMMR to recover information and provides denoising capabilities. We conducted comprehensive tests on different SRT datasets, including samples profiled by 10x Visium, NanoString technology and Spatial Transcriptomics (ST) technology. stMMR outperforms SOTA techniques in terms of domain identification, pseudo-spatiotemporal analysis and domain-specific gene discovery. The experimental results on breast cancer and lung cancer [26] demonstrated that stMMR accurately identifies tumor edges and tumor-infiltrating regions, proving its potential value in clinical research. Overall, the stMMR exhibits exceptional capability in the multi-modal feature representation of SRT, providing a powerful new tool for accurate and robust domain identification.

Methods

Overview of stMMR

The multi-modal joint representation process of stMMR primarily consists of the following three steps: multi-modal feature embedding, feature fusion and feature reconstruction. The overall workflow of stMMR is illustrated in Fig. 1 and the detailed implementation are introduced in Supplementary Section 1.

Multi-modal feature embedding

The stMMR initially performs embedding on gene expression, spatial location, and histology image information. For gene expression data, $G \in \mathbb{R}^{N \times P}$ represents the normalized gene expression matrix, where N is the number of spots and P is the number of identified high variance genes. For histological images, we use a pre-trained Vision Transformer (ViT) model [27] to extract the image features matrix $H \in \mathbb{R}^{N \times M}$, where M is the output dimension. To encode the spatial location information, we construct an undirected weighted graph to present SRT data, and the adjacency matrix A is defined as:

$$A_{ij} = exp\left(-\frac{d(i,j)^2}{2l^2}\right) \tag{1}$$

where $d(i, j)$ represents the Euclidean distance between spots i and j, and l is used to control the relationship between weight and distance. A larger value of l implies a faster decay of weight with increasing distance.

Next, we employ a two-layer GCN encoder for message passing and aggregation of image features and gene expression features [28]:

$$E^{(k)} = \tilde{D}^{-\frac{1}{2}}\tilde{A}\tilde{D}^{-\frac{1}{2}}E^{(k-1)}W^{(k-1)} \tag{2}$$

where $E^{(k)}$ and $E^{(k-1)}$ represent the input and output of GCN encoder. $E^{(0)}$ can be the image features H or gene expression features G. $\tilde{A} = A + I$ denotes the symmetrically normalized adjacency matrix, where I is the identity matrix. $\tilde{D}$ and $W^{(k-1)}$ are the weighted degree matrix and trainable parameter respectively. The visual features and gene expression features obtained after the encoder are denoted as E_H and E_G.

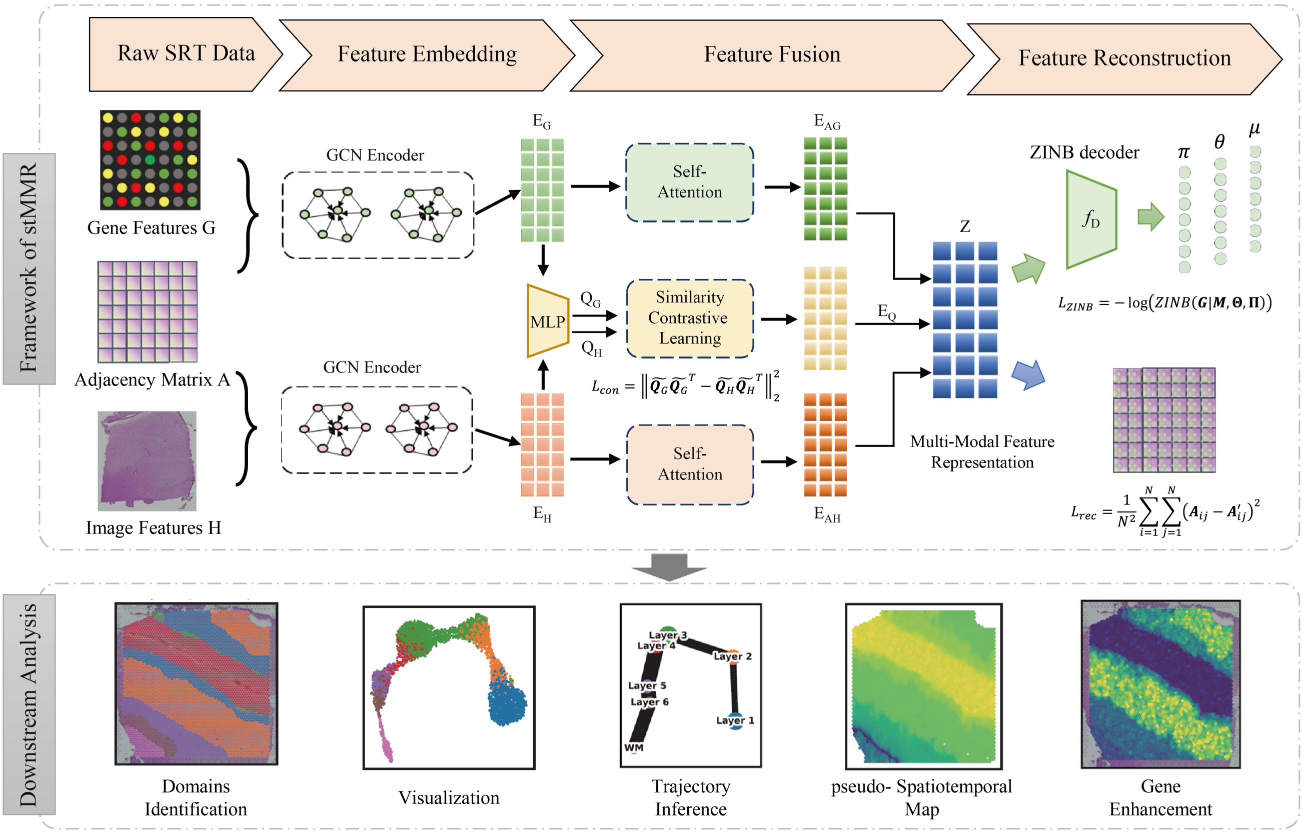

Figure 1. Schematic overview of stMMR for the joint representation of features from different modalities. Gene expression and histology image information are embedded using GCN module based on adjacent matrix. Then, the relationships between different modalities are captured through similarity contrast learning, followed by feature fusion. Finally, the original features are reconstructed from the multi-modal feature representation. This representation can be used for downstream analysis directly.

Feature fusion

We propose a novel strategy for multi-modal information aggregation. Biological structures often exhibit relationships between distant regions that cannot be captured by local spatial dependencies alone. For instance, in complex tissues such as the brain or cancerous tissues, cells in non-adjacent spatial domains can share similar gene expression patterns or morphological characteristics. Therefore, stMMR uses a normalized attention module to learn the global relationships between spots in a single modality, as shown in Eq.3:

$$E_A = softmax\left(\frac{E \cdot E^T}{\sqrt{d}}\right) \cdot E \tag{3}$$

where E represents the imaging features E_H or transcriptomics features E_G from the previous step. New features obtained through the attention module are E_{AH} and E_{AG}. Notably, a nonlinear activation function and Euclidean distance matrix d normalize the weights, preventing the local optima issue from oversized weights for certain spots [29].

stMMR uses contrastive learning for cross-modal feature fusion, highlighting the consistency between modalities like morphology and gene expression, which have both similarities and complementary relationships [30, 31, 32]. It maps the latent features E_H and E_G through two fully connected neural networks to obtain hierarchical representations Q_H and Q_G, as shown in Eq.4:

$$Q = Relu(W_Q E + b_Q) \tag{4}$$

where E represents the morphological features E_H or gene expression features E_G, and Q corresponds to Q_H or Q_G. W_Q and b_Q are the parameters of the fully connected network.

After obtaining low-dimensional features Q_H and Q_G for the two modalities, a fully connected neural network is employed to fuse them, as shown in Eq.5:

$$E_Q = W_E \cdot concat(Q_G, Q_H) + b_E \tag{5}$$

where W_E and b_E are the parameters of the fully connected network.

To enhance the consistency between Q_H and Q_G, we use a constraint as shown in Eq.6, replacing the loss of traditional contrastive learning:

$$L_{con} = \left\| \widetilde{Q_G}\widetilde{Q_G}^T - \widetilde{Q_H}\widetilde{Q_H}^T \right\|_2^2 \tag{6}$$

where $\widetilde{Q_G}$ and $\widetilde{Q_G}$ are the normalization matrices of Q_G and Q_H, respectively.

Finally, we further integrate modality specific features E_{AH} and E_{AG} obtained from Eq.3 with the cross-modality features E_Q obtained from Eq.5 to get the multi-modal feature representation Z, as shown in the following equation:

$$Z = \alpha E_Q + \beta E_{AH} + \gamma E_{AG} \tag{7}$$

where α, β, and γ are hyperparameters for adjusting the importance of features.

Feature reconstruction

stMMR adopts the zero-inflated negative binomial (ZINB) decoder [33, 34] to reconstruct gene expression information and the adjacency matrix is estimated directly using the concept of a graph

auto-encoder [35, 36]:

$$L_{ZINB} = -\log\left(ZINB(G|M, \Theta, \Pi)\right) \quad (8)$$

$$A' = Sigmoid\left(\frac{Z \cdot Z^T}{\|Z\|_2 \cdot \|Z^T\|_2}\right) \quad (9)$$

where M, Θ, and Π are the mean, dispersion, and dropout probability of the output from network respectively.

Subsequently, the regularization loss between the reconstructed matrix and the adjacency matrix can be computed:

$$L_{rec} = \frac{1}{N^2}\sum_{i=1}^{N}\sum_{j=1}^{N}\left(A_{ij} - A'_{ij}\right)^2 \quad (10)$$

Objective function

Finally, we integrated Eq.6, Eq.8, and Eq.10 to formulate the final objective function:

$$L = a * L_{con} + b * L_{ZINB} + c * L_{rec} \quad (11)$$

In this equation, a, b, and c are weight for the different loss terms.

For detailed information on the training process and parameter settings, please refer to the Supplementary Section 1.

Benchmark Methods

To demonstrate the effectiveness of the multi-modal feature representation in SRT data, we selected 7 SOTA methods for benchmarking comparison. These methods include SCANPY [37], which utilizes only gene expression data; CCST, STAGATE, GraphST, and SpaceFlow, which employ both gene expression and spatial location information [20, 21, 22, 23]; stLearn and DeepST, which incorporate all three modalities [24, 25]. Methods that have already been compared in previous works are not included in our analysis [38, 39, 40].

Results

stMMR enhances detection of stratified architectural patterns in human dorsolateral prefrontal cortex (DLPFC) tissue

The spatial structure of the brain is closely related to its function, particularly evident in the layered organization of the human brain cortex [41]. To explore the spatial structure arrangement of brain , we collected a 10x Visium dataset containing 12 dorsolateral prefrontal cortex (DLPFC) sections [42]. The histology image and manually annotated layers are illustrated in Fig. 2A.

We initially compared the Adjusted Rand Index (ARI) levels of various methods across 12 slices of the DLPFC dataset (Fig. 2B). The result reveals that stMMR outperformed other methods, achieving the highest ARI and the smallest variance compared to manual annotations. Notably, the results from STAGATE, CCST, and SpaceFlow show differences in the ARI across different slices, indicating that these methods are more sensitive to the domain patterns. Scanpy uses only gene expression information and shows the poorest performance. Methods like stlearn and DeepST, which integrate histological information, are outperformed by GraphST and STAGATE. This underperformance might stem from insufficient integration of transcriptomic and imaging data.

Next, we conducted a detailed analysis for each slice (Fig. 2C and Supplementary Fig. S1). To demonstrate the results, we used slice 151509 as an example (Fig. 2C-E). The results shows that DeepST struggle with rough segmentation between layers. CCST, SpaceFlow, and stLearn have issues with erroneous region identification. Although GraphST and STAGATE accurately discern the arrangement of different regions, these methods exhibit biases in identifying the boundaries between distinct domains. In this specific case, stMMR demonstrates exceptional domain identification results. We further utilize UMAP for low-dimensional visualization analysis of the results obtained from different methods (Fig. 2D), to verify whether the embeddings can accurately encompass information on regional arrangement and boundaries. The analysis reveals that techniques such as stMMR, CCST, STAGATE, and stLearn effectively separate different domains. In contrast, GraphST, SpaceFlow and DeepST exhibit noticeable issues in layer boundaries. For instance, the boundaries between layers 2, 3, and 4 are confused.

Further, we conducted a detailed trajectory inference using the PAGA algorithm [43] for these methods (Fig. 2E). The PAGA graphs indicates that stMMR, STAGATE, CCST, and stLearn performs well in predicting trajectory between adjacent layers. The other methods display confused results in this analysis.

Combining the insights from these various analyses, it is evident that stMMR remarkably effective in domain identification and trajectory inference. These results adequately demonstrate effective capability of stMMR in integrating transcriptomic and histological data.

stMMR enhances spatial gene expression profiling and structural characterization

In SRT, the analysis of domain-specific genes holds significant importance. However, identifying domain-specific genes which have relationships with histological structures is challenging. This is primarily due to the presence of substantial noise in the gene expression profiles generated by SRT techniques, such as the dropout event [44, 45, 46]. To validate whether stMMR can enhance gene expression data through histological information, we analyzed domain-specific genes identified using the original gene expression profile and the profile reconstructed through the ZINB decoder [47].

Using both original and reconstructed gene expression data, genes such as AQP4 and HPCAL1 are recognized as layer-specific genes. These genes are enriched in multiple layers and have been confirmed through multiplex single-molecule fluorescent in situ hybridization [42]. However, employing reconstructed gene expression data facilitates the identification of new domain-specific genes. For instance, with the enhancement of stMMR, CACNA2D2 and ADCYAP1 can be identified as domain-specific genes in layer 3. Previous research has found that in layer 3 of primates, the CACNA2D2 gene exhibits differential expression and is closely associated with several biological pathways, including calcium signaling and synaptic long-term depression [48]. ADCYAP1 has also been proved to be a domain-specific gene in former study [49]. This suggests that the expression patterns after stMMR enhancement are more consistent with known neurobiological functions and pathological states.

We also conducted a more detailed analysis by combining gene expression levels with their spatial locations (Fig. 3). We found that after enhancement with stMMR, more distinct expression patterns of domain-specific genes can be observed. Specifically, Fig. 3A demonstrates a clear spatial representation of domain-specific marker genes (ADAYAP1, CACNA2D2, CALB1, MARC1, MB and LPL) after data enhancement. In the original data, the expression pattern of genes such as ADCYAP1, CACNA2D2 and MB are sparse, and the boundaries in spatial regions are blurred, making it difficult to discern a clear expression pattern (Fig. 3A and B). However, after enhancement with stMMR, we can observe that CACNA2D2 and CALB1 exhibit much clearer expression patterns in layers 2 and 3. Additionally, the enrichment of LPL in the layer 1 become more pro-

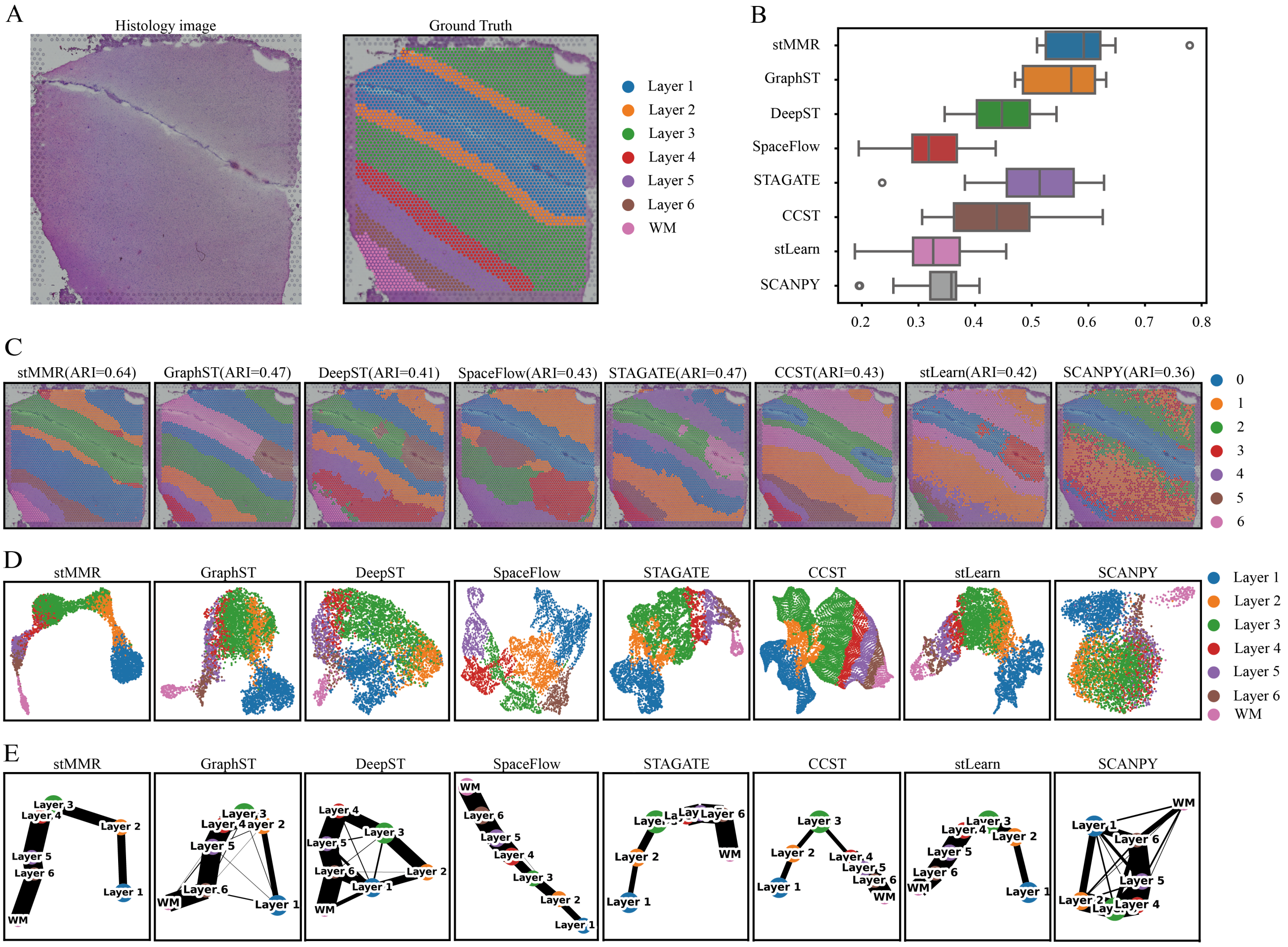

Figure 2. Performance comparisons of different methods on DLPFC datasets. (A) The histology image and manually annotated brain regions of slice 151509. (B) The overall performance of 8 different methods across 12 slices. (C) The domain recognition results on slice 151509. (D) The UMAP visualization results of the embeddings from 8 different methods on slice 151509. (E) The inferenced trajectories on slice 151509.

nounced (Fig. 3A and C). These results reflect that stMMR not only improves the spatial resolution of gene expression patterns using histological information but also enhances our understanding of the subtle differences in gene expression across different regions of the brain.

stMMR deciphers evolving cell lineage structures in chicken heart ST dataset

Analyzing temporal SRT data can reveal the dynamic domain changes during the development of tissue organs. We collected the chicken heart SRT dataset to further investigate the effectiveness of stMMR in the integrated representation of multi-modal features [50]. This dataset includes 12 tissue slices, collected on day 4 (5 slices), day 7 (4 slices), day 10 (2 slices), and day 14 (1 slices), documenting four key stages of the Hamburger-Hamilton ventricular developmental stages [50]. We selected SpaceFlow as the baseline because it was specifically designed to capture spatiotemporal dynamics.

We annotated the slices of different developmental stages using labels provided by the original research (Fig. 4A) [50]. Subsequently, we employed the embeddings from stMMR and SpaceFlow to identify domains of chicken heart across these four distinct stages. Fig. 4B indicate that the regions detected by stMMR largely coincide with manual annotation. For instance, major regions of the chicken heart, such as atrial cells and the inter-ventricular septum, are accurately identified. Notably, stMMR also detects domains that are hard to identified (Fig. 4B). For example, in the data from days 7, 10, and 14, the epicardium, a thin layer surrounding the outer side of the chicken heart, is clearly identified by stMMR. Although there are some instances of misclassification in the characterization of spot features using stMMR in a few regions, the identification of the epicardium is quite clear (Fig. 4B).

Next, we adopted a method similar to previous study to analyze the pseudo-spatiotemporal map (pSM) [22]. In brief, we mapped the spot features obtained through stMMR and SpaceFlow on the pseudo-temporal axis [22, 37, 51]. These points reflect the relative positions of cells in their developmental trajectory or functional state. As clearly visible in Fig. 4C, within the D7 to D14, the valve structures can be distinctly identified through the pSM values. Moreover, the representation of the myocardium in ventricles, as indicated by the pSM values, appears more uniform (yellow area) compared to the regional segmentation results in Fig. 4B. According to related research [52], the endocardium, the inner layer of the heart, is one of the early events in cardiac formation. The endocardial tubes are fundamental to cardiac development, eventually merging to form the primitive heart tube. As the heart tube forms, myocardial development commences, followed closely by the development of the atria. In our analysis, we observe that the myocardium in ventricles (yellow area in Fig. 4C) consistently shows higher pSM values compared to other areas in the same stage, indicating a later pseudo-temporal ordering of the ventricular myocardium [22]. Additionally, the pseudo-temporal ordering of the

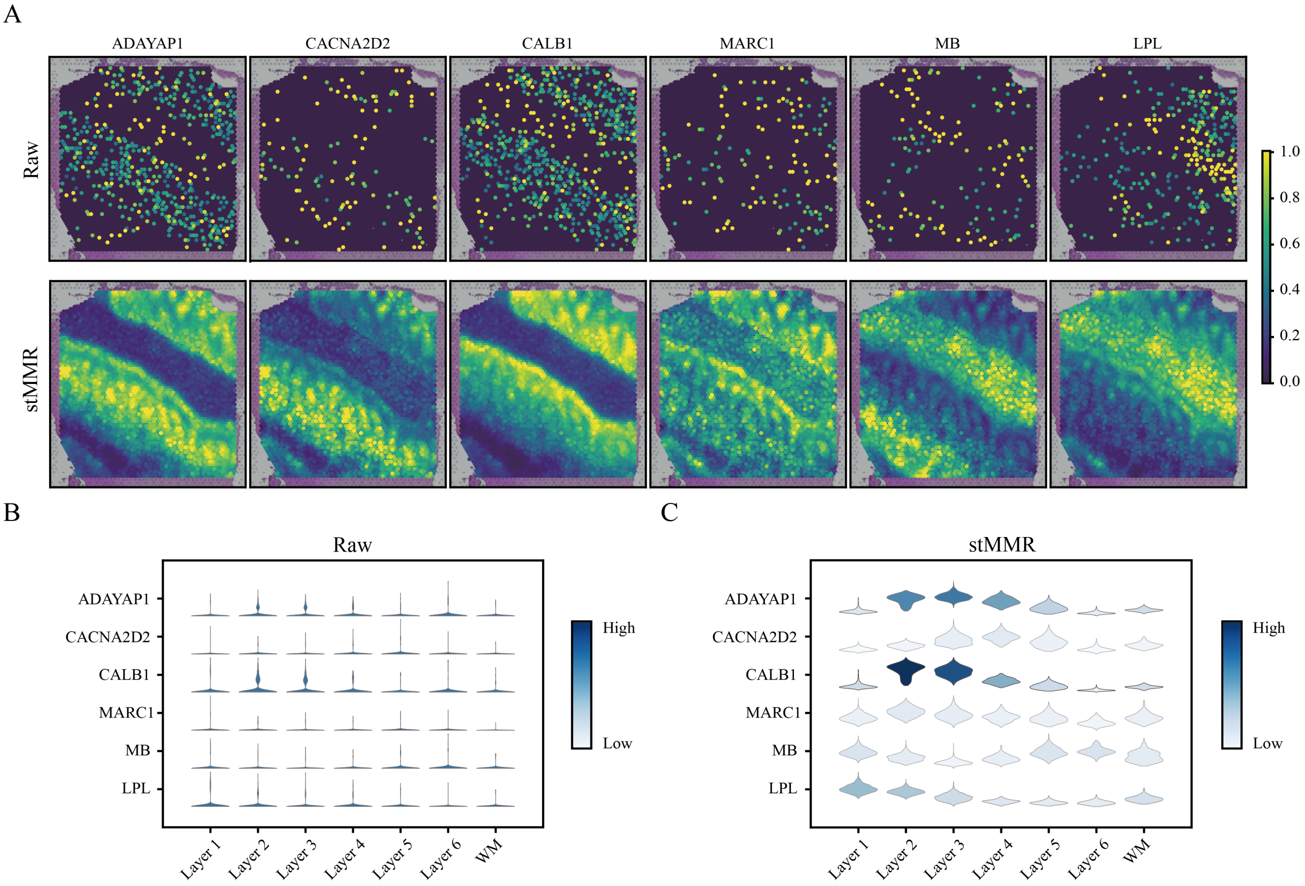

Figure 3. stMMR enhances spatial gene expression profiles and spatial structural characterization. (A) Spatial representation of layer-specific marker genes before and after data enhancement. (B) Gene expression level before and after data enhancement.

atria (marked in teal) follows that of the valves, suggesting that the development of the atria occurs after the valves. Therefore, the pSM derived from stMMR accurately displays the developmental sequence of the chicken heart. We further identified domain-specific genes through differential expression analysis across regions. For instance, we observed that MYH7 is highly specifically expressed in the Atria. This finding aligns with previous reports on the analysis of Atria and Ventricles specific proteins [53].

stMMR accurate identifies tumor region in human breast cancer

Breast cancer is a major type of cancer worldwide [54]. We collected a human breast cancer dataset from 10x Visium platform to conduct an in-depth analysis of the microenvironment in breast cancer (Fig. 5A).

First, we applied different methods for domain identification. From the results presented in Fig. 5B, it is observed that stMMR shows the most outstanding performance in category labeling. In terms of regional continuity, stMMR also demonstrates superior performance among different methods. Taking the IDC_5 area in the upper left corner as an example, this area occupies a significant portion in invasive ductal carcinoma, with a notable increase in cancer cells compared to normal tissue or non-tumorous areas [23]. However, only stMMR accurately identified the entire IDC_5 area, demonstrating higher precision compared to other methods. Additionally, stMMR also exhibits higher continuity in predicting the Tumor_edge area, whereas the results of other methods appear more dispersed in this aspect.

Next, we conducted a comprehensive analysis of domain-specific genes between merged tumor and normal regions (Supplementary Section 2.3). We utilized the DisGeNET to delve into the domain-specific genes of tumor regions [55]. Our analysis revealed that these domain-specific genes are enriched in several breast cancer related terms such as non-Hodgkin lymphoma and inflammation (Fig. 5C). Studies have shown that the development of breast cancer significantly increases the risk of non-Hodgkin lymphoma, particularly follicular lymphoma and mature T/NK cell lymphomas [56]. Numerous studies also have indicated that inflammation plays a regulatory role in the development of cancer and its response to treatment [57, 58, 59]. To further validate our research findings, we conducted an analysis of the transcriptional regulatory network using TRRUST [60]. The results indicated that multiple top-ranked terms are closely associated with breast cancer (Fig. 5C). For instance, the key regulatory factors (SP1, NFKB1, RELA and TP53) from the top four terms have been confirmed to play pivotal roles in the development and progression of breast cancer [61, 62, 63, 64, 65, 66].

stMMR dissects cell type differences in a lung cancer SRT dataset based on NanoString technology

To further validate the generalization ability and applicability of stMMR, we applied stMMR to the single-cell SRT dataset generated by NanoString CosMx SMI. This dataset comprises lung cancer tissue samples from 20 fields of view (FOVs) [26], and covering eight major cell types (Fig. 6A and E)

We employed the benchmarking methods to identify spatial domains within 20 FOVs, as shown in Fig. 6B, D-F. Fig. 6B revealed that stMMR closely aligns with the original study in detecting the spatial distribution of cell types, particularly in identifying tumor cells. In the overall analysis of the 20 sections, the performance

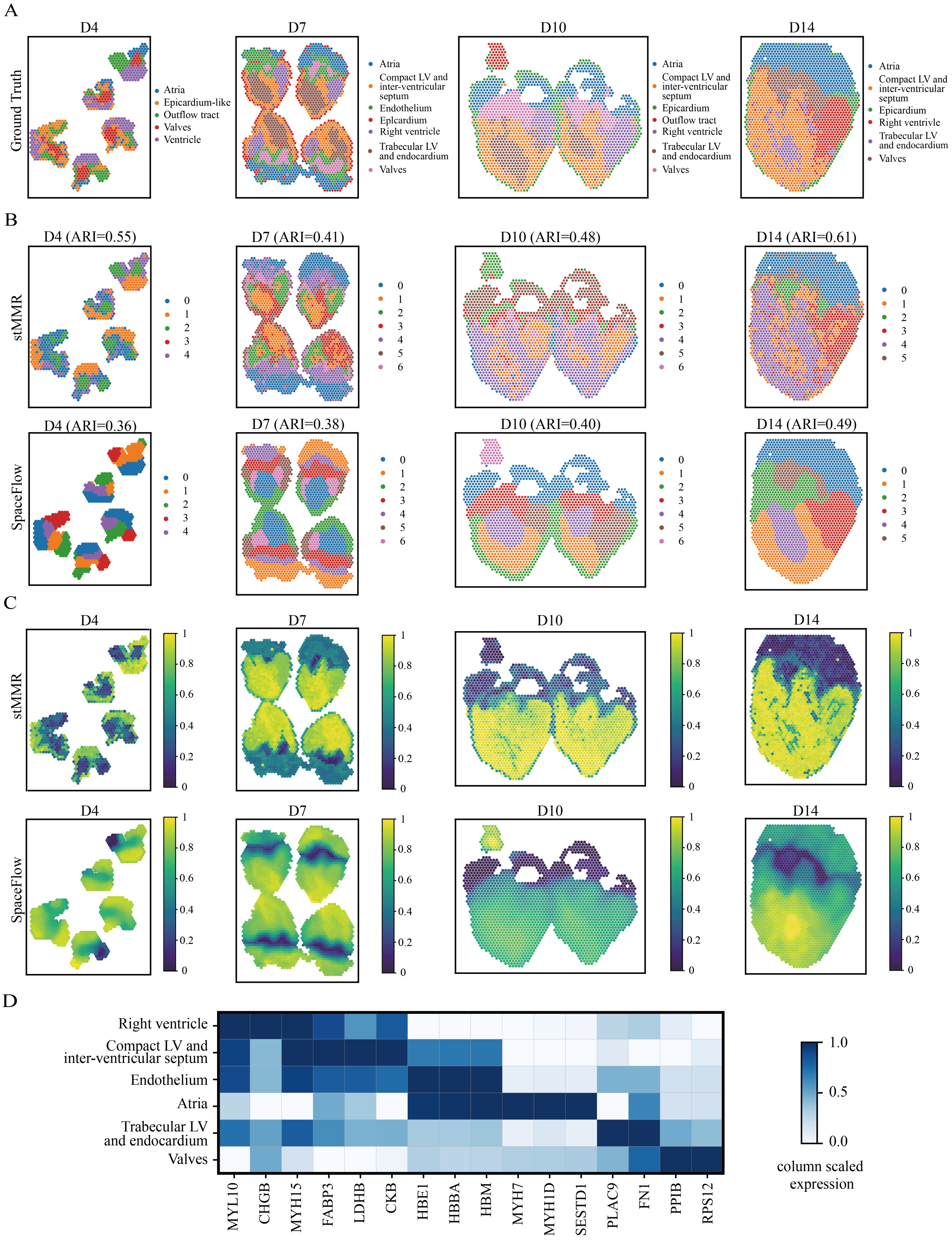

Figure 4. stMMR reveals cell lineage structures during chicken heart development. (A) The ground truth label provided by the original data. (B) The domains recognized by stMMR and SpaceFlow. (C) The plots of pSM value from stMMR and SpaceFlow for illustrating pseudo-temporal developmental trajectory. (D) The differentially expressed marker genes discovered by stMMR.

of stMMR is superior to other methods (Fig. 6D). Furthermore, we conducted a cell type-specific gene analysis based on the cell annotations in one slice. We observe that different cells exhibit unique expression patterns (Fig. 6C). For instance, Igkc transcripts,

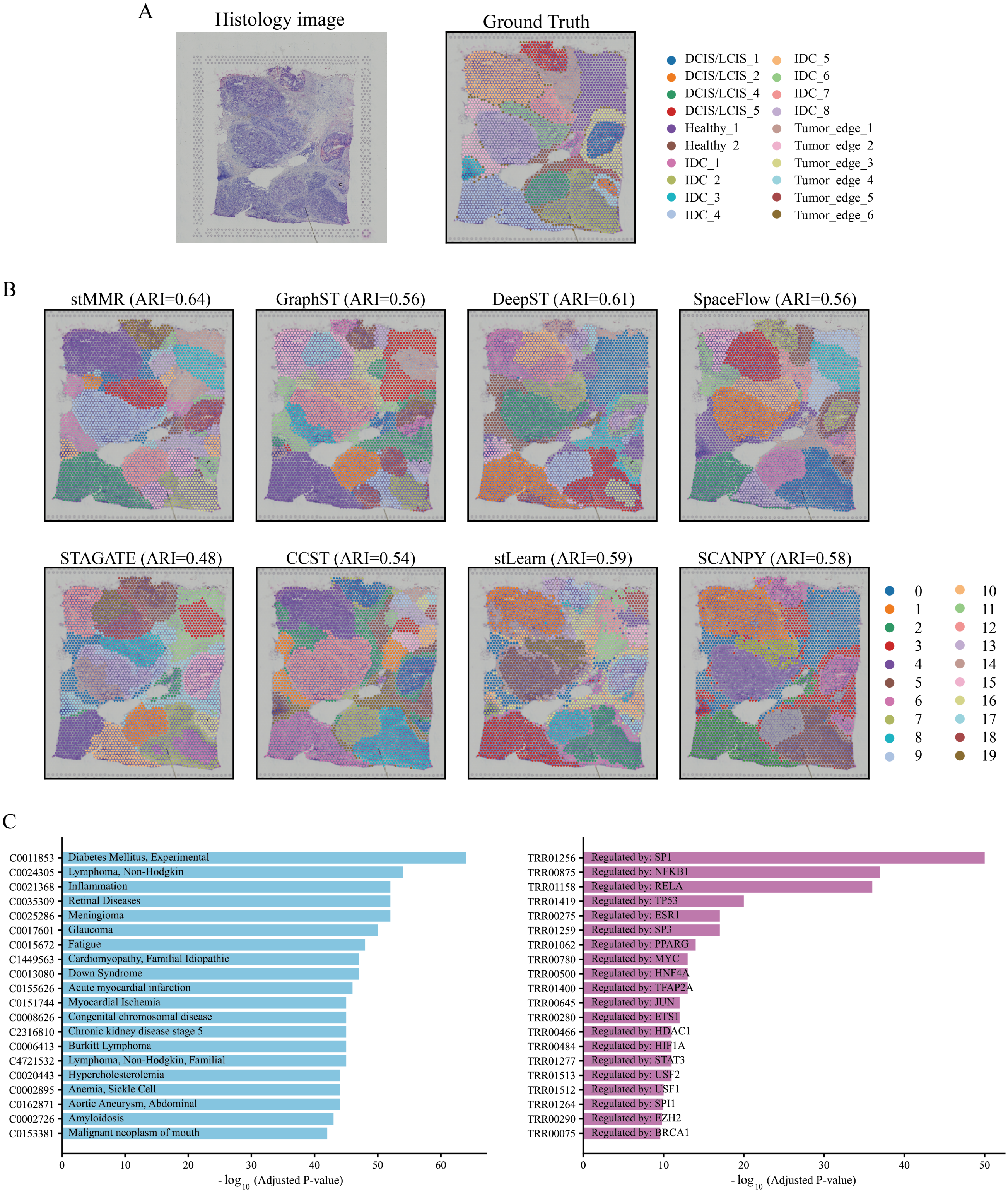

Figure 5. stMMR identifies tumor region in human breast cancer dataset. (A) The H&E images and the manually annotated regions. (B) The annotation results from different methods. (C) Top 20 differentially expressed gene enriched terms identified by DisGeNET (left panel) and TRRUST (right panel).

previously reported to be upregulated in myeloid progenitor populations, is also confirmed in our study [67]. The genes COL3A1 and COL1A1 shows significant positive correlations with neutrophils [68, 69]. Additionally, the oncogene SOX4 is prominently featured in our differential analysis of tumor cells [70]. These genes are also identified as diagnostic or prognostic biomarkers in previous studies [68, 71, 72, 73, 74]. Notably, some cell types also share similar gene expression patterns (Fig. 6C). For example, epithelial cells and tumor cells exhibit expression similarities. Multiple studies using single-cell transcriptomics analysis have revealed that lung cancer cells share characteristics similar to those of Type 1 and Type 2 alveolar epithelial cells [75, 76]. This similarity may be related to lung cancer cells maintaining epithelial cell functions, such as cell adhesion and migration [77, 78].

We also conducted a visualization analysis comparing the results of stMMR applied to 20 tissue sections with the actual division

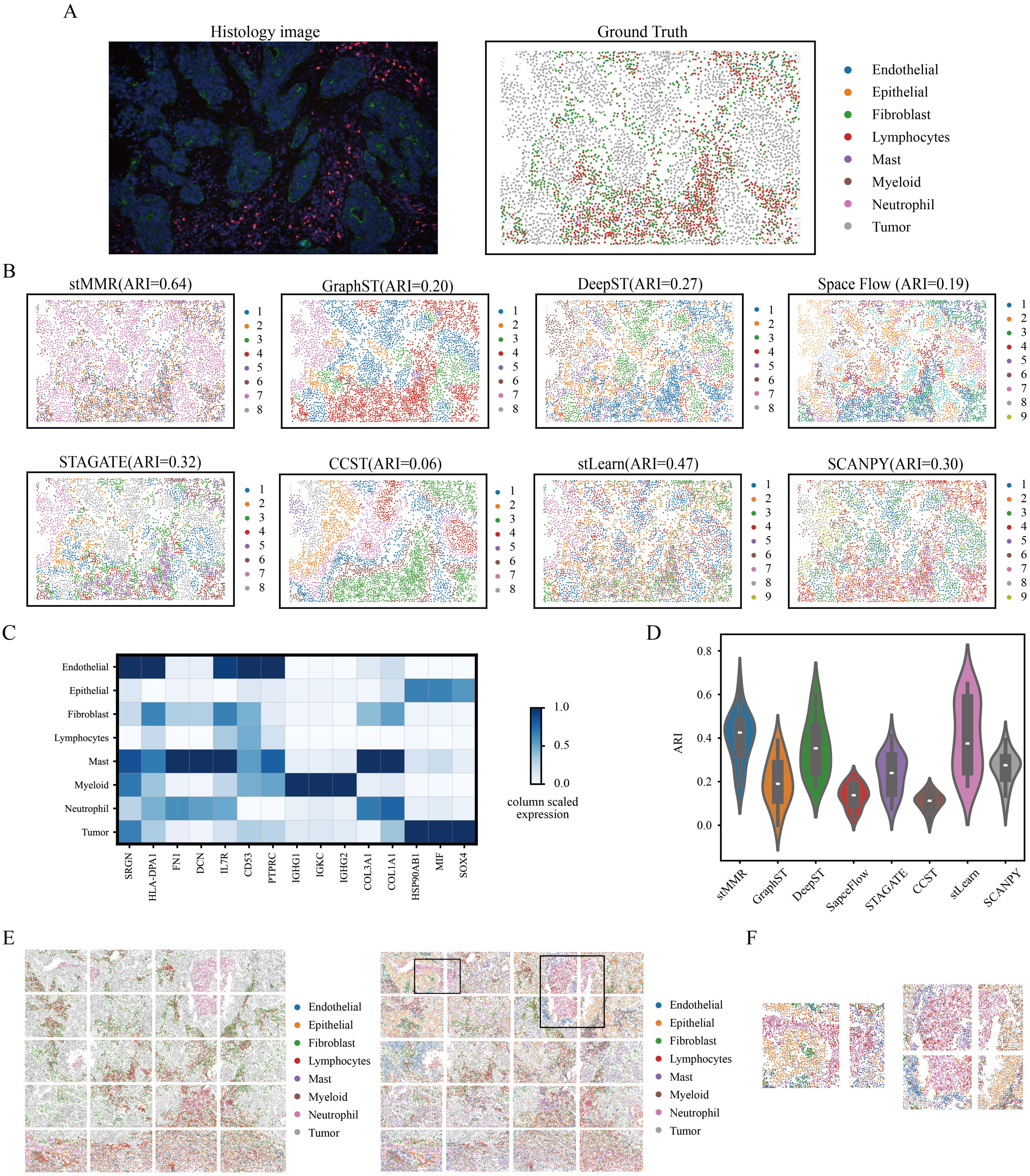

Figure 6. stMMR recognizes cell type differences in lung cancer dataset. (A) One FOV of the lung cancer SRT data. (B) Cell types identified by different methods. (C) Expression pattern of marker genes for different cell types. (D) The overall performance of different methods across 20 FOVs. (E) Cell types annotated manually in 20 FOVs. (F) Cell types annotated by stMMR in 20 FOVs. (G) The zoomed-in results of boundaries between adjacent FOVs identified by stMMR.

of tissue regions. The analysis demonstrates that stMMR effectively identifies tissue regions across multiple sections (Fig. 6E). Notably, even in regions bisected by section boundaries, stMMR maintains smooth and continuous (Fig. 6E and F). These findings indicate that the joint representation of stMMR not only effectively eliminates noise from different data types but also maintains excellent performance in the recognition of tissue regions across multiple slices.

Discussion

SRT technology enables us to deeply understand the spatial structure of tissues within biological systems from multiple dimensions, including gene expression profiles, spatial location, and histological information. However, the inherent data heterogeneity along with the varying spatial resolutions presents challenges in the integration of these modalities. To harmonize and unify multi-modal data as well as achieve effective joint representation for multi-modal SRT data, we propose a novel computational framework, stMMR.

stMMR effectively unifies gene expression profiles and histological information by utilizing spatial location as a connecting link. This method automates the construction of adjacency relationships between neighboring spots. Then, GCN is employed to extract features from both gene expression profiles and histological information. Furthermore, stMMR adopts an innovative strategy for representing intra-modal and inter-modal features. Initially, it employs an attention mechanism for an in-depth learning within a single modality. It then integrates cross-modality features through a combination of similarity contrastive learning, along with the reconstruction of gene expression and adjacency relationship. By applying stMMR to SRT data of various tissues and resolutions, we have validated its exceptional performance in multiple analyses, including domain identification, pseudo-spatiotemporal analysis, gene expression data enhancement as well as the identification of domain-specific genes.

The remarkable performance of stMMR can be attributed to several innovative designs, as confirmed by ablation studies (Supplementary Fig. S2). The crucial aspect is the integration of histological information with gene expression data through spatial location. In SRT, gene expression data suffers from issues of sparsity and zero inflation, which are key factors that interfere with downstream analysis [33, 79]. Previous research has shown that histological information can predict gene expression data [30, 31, 32]. Therefore, compared to methods that rely only on gene expression information, stMMR integrates imaging information and exhibits superior performance in spot characterization. Secondly, unlike other methods that construct spatial transcriptomic data as unweighted graphs, stMMR builds undirected weighted graphs inversely proportional to Euclidean distances between spots, better reflecting the influence of spatial distance on message passing and aggregation. Furthermore, the consideration of relationships within and between modalities is also crucial. Sole reliance on gene expression data for correlation analysis may result in information loss. In contrast, methods that incorporate imaging information, such as DeepST, focus primarily on the integration of multi-modal data, overlooking the relationships within individual modalities. To fully leverage the relationships within and between modalities, stMMR not only uses similarity contrastive learning for integrating features across modalities but also incorporates a self-attention module for deep embedding of features within a modality. Additionally, the reconstruction modules for gene expression and adjacency matrix further encourage the model to retain as much original information as possible. This encoder-decoder structure improves the ability of stMMR to recover information also endows stMMR with denoising capabilities and robustness.

Notably, stMMR possesses strong scalability. It can be easily applied to other data derived from diverse experimental techniques. Beyond the previously mentioned datasets, we applied stMMR to the analysis of a mouse brain dataset derived using 10x Visium technology, a human pancreatic ductal adenocarcinoma dataset obtained through ST technology, and a human colorectal cancer (CRC) dataset generated using the newly introduced 10x Visium HD technology (Supplementary Fig. S3, S4 and S5). In these tests, the stMMR consistently achieved optimal results. Furthermore, we extended stMMR to integrate and analyze multi-slice data (Supplementary Fig. S6). Additionally, by duplicating the gene expression module, the framework of stMMR can be directly used for integration of features from proteomes or epigenomics [80, 81, 82, 83].

There is still room for the improvement of stMMR. Currently, stMMR employs Euclidean distance in the construction of spot adjacency matrices. However, in practical scenarios, it may be more rational to utilize different distance metrics for graph construction based on modal features. For instance, considering gene expression data, the use of Pearson Correlation Coefficients or K-L divergence might be more appropriate to measure expression similarity between spots. In contrast, for spatial imaging data, either Euclidean distance or staining similarity can serve as the distance metric. Under these circumstances, the constructed graph transitions from being a homogenous graph to a heterogeneous one. For such heterogeneous graphs with multiple types of edges, we can apply methods like metapath2vec or multi-view learning to achieve embedding and integration of different modalities [84, 85, 86, 87].

In this paper, we introduce a robust and accurate tool, stMMR, for the integration of gene expression data, spatial information, and histological information from SRT data. Compared to existing methods, stMMR demonstrates a significant advantage in integrating multi-modal data, particularly excelling in domain identification, pseudo-spatiotemporal analysis, and domain-specific gene analysis. Overall, as an effective and user-friendly tool, stMMR enhances the multi-modal joint analysis of SRT data, providing substantial support for research in relevant fields.

Availability of Source Code and Requirements

Project name: stMMR
Project home page: https://github.com/nayu0419/stMMR
Operating system(s): Linux
Programming language: Python
Other requirements: Python 3.9.1 or higher
License: MIT license
RRID: SCR_025601
BiotoolsID: stmmr

Additional Files

Supplementary Fig. S1. The results of the regions identified by stMMR in 12 DLPFC slices.

Supplementary Fig. S2. Ablation study results for stMMR on a breast cancer dataset. (A) The spatial clustering result of stMMR. (B) stMMR without histological information, resulting in a lower ARI of 0.57, indicating the importance of histology in improving spatial domain identification. (C) stMMR without GCN, which yields a significantly reduced ARI of 0.49, showing the critical role of GCN in capturing local spatial relationships. (D) stMMR without the attention mechanism, leading to a decrease in ARI to 0.60, demonstrating the importance of global attention in enhancing overall model performance.

Supplementary Fig. S3. The domain recognition results of 10x mouse brain dataset. (A) The histology image and (B) manually annotated tissue structures. (C) The domain recognition results by different methods.

Supplementary Fig. S4. The domain recognition results of human pancreatic ductal adenocarcinoma. (A) The histology image and (B) annotated tissue structures. (C) The domain recognition results by different methods.

Supplementary Fig. S5. Spatial clustering results on the human colorectal cancer. (A) The histology image. (B) The domain recognition results by different methods.

Supplementary Fig. S6. Spatial domain identification results from stMMR integrating four slices, and the ground truth for the four slices.

Supplementary Table S1. Summary of the datasets used in this study.

Data availability

All datasets used in this paper are publicly available and listed in Supplementary Section 2. Processed datasets are also available at SODB (https://gene.ai.tencent.com/SpatialOmics/) and can be loaded by PySODB (https://protocols-pysodb.readthedocs.io/en/latest/). The domain-specific genes found by stMMR are available on the stMMR GitHub page.

Abbreviations

SRT: spatially resolved transcriptomics; GCN: graph convolutional networks; H&E: hematoxylin and eosin; ViT: Vision Transformer; ZINB: zero-inflated negative binomial; DLPFC: dorsolateral prefrontal cortex; pSM: pseudo-spatiotemporal map; ARI: Adjusted Rand Index; CRC: colorectal cancer.

Competing Interests

The authors declare they have no competing interests.

Funding

This work was supported by the National Natural Science Foundation of China (Nos. 62303271, U1806202, 62373216, 92374107)and Natural Science Foundation of Shandong Province (ZR2023QF081).

Authors' Contributions

R.G., W.Z. and Z.Y. conceived and supervised the project. D.Z. and N.Y. designed the model and developed the stMMR software. D.Z., N.Y. and W.Z. wrote the manuscript. X.S. and Q.Z. collected and constructed the benchmark datasets. W.L. X.L. and Z.L. conducted biological interpretation. All authors approved the manuscript.

Acknowledgements

Not applicable.

References

1. Chen KH, Boettiger AN, Moffitt JR, et al. Spatially resolved, highly multiplexed RNA profiling in single cells. Science 2015;348(6233):aaa6090. https://doi.org/10.1126/science.aaa6090.
2. Asp M, Bergenstråhle J, Lundeberg J. Spatially Resolved Transcriptomes—Next Generation Tools for Tissue Exploration. BioEssays 2020;42(10):1900221. https://doi.org/10.1002/bies.201900221.
3. Eng CHL, Lawson M, Zhu Q, et al. Transcriptome-scale super-resolved imaging in tissues by RNA seqFISH+. Nature 2019;568(7751):235–239. https://doi.org/10.1038/s41586-019-1049-y.
4. Wang X, Allen WE, Wright MA, et al. Three-dimensional intact-tissue sequencing of single-cell transcriptional states. Science 2018;361(6400):eaat5691. https://doi.org/10.1126/science.aat5691.
5. Ståhl PL, Salmén F, Vickovic S, et al. Visualization and analysis of gene expression in tissue sections by spatial transcriptomics. Science 2016;353(6294):78–82. https://doi.org/10.1126/science.aaf2403.
6. Rodriques SG, Stickels RR, Goeva A, et al. Slide-seq: A scalable technology for measuring genome-wide expression at high spatial resolution. Science 2019;363(6434):1463–1467. https://doi.org/10.1126/science.aaw1219.
7. Marx V. Method of the Year: spatially resolved transcriptomics. Nat Methods 2021;18(1):9–14. https://doi.org/10.1038/s41592-020-01033-y.
8. Guo T, Yuan Z, Pan Y, et al. SPIRAL: integrating and aligning spatially resolved transcriptomics data across different experiments, conditions, and technologies. Genome Biol 2023;24(1):241. https://doi.org/10.1186/s13059-023-03078-6.
9. Huo Y, Guo Y, Wang J, et al. Integrating multi-modal information to detect spatial domains of spatial transcriptomics by graph attention network. J Genet Genomics 2023;50(9):720–733. https://doi.org/10.1016/j.jgg.2023.06.005.
10. Son CG, Bilke S, Davis S, et al. Database of mRNA gene expression profiles of multiple human organs. Genome Res 2005;15(3):443–450. https://doi.org/10.1101/gr.3124505.
11. Hannig J, Schäfer H, Ackermann J, et al. Bioinformatics analysis of whole slide images reveals significant neighborhood preferences of tumor cells in Hodgkin lymphoma. PLoS Comput Biol 2020;16(1):e1007516. https://doi.org/10.1371/journal.pcbi.1007516.
12. Haghighi M, Caicedo JC, Cimini BA, et al. High-dimensional gene expression and morphology profiles of cells across 28,000 genetic and chemical perturbations. Nat Methods 2022;19(12):1550–1557. https://doi.org/10.1038/s41592-022-01667-0.
13. Li Z, Chen X, Zhang X, et al. Latent feature extraction with a prior-based self-attention framework for spatial transcriptomics. Genome Res 2023;33(10):1757–1773. https://doi.org/10.1101/gr.277891.123.
14. Li Z, Zhou X. BASS: multi-scale and multi-sample analysis enables accurate cell type clustering and spatial domain detection in spatial transcriptomic studies. Genome Biol 2022;23(1):168. https://doi.org/10.1186/s13059-022-02734-7.
15. Zhao E, Stone MR, Ren X, et al. Spatial transcriptomics at subspot resolution with BayesSpace. Nat Biotechnol 2021;39(11):1375–1384. https://doi.org/10.1038/s41587-021-00935-2.
16. Dries R, Zhu Q, Dong R, et al. Giotto: a toolbox for integrative analysis and visualization of spatial expression data. Genome Biol 2021;22:78. https://doi.org/10.1186/s13059-021-02286-2.
17. Varrone M, Tavernari D, Santamaria-Martínez A, et al. CellCharter reveals spatial cell niches associated with tissue remodeling and cell plasticity. Nat Genet 2024;56(1):74–84. https://doi.org/10.1038/s41588-023-01588-4.
18. Liu W, Liao X, Luo Z, et al. Probabilistic embedding, clustering, and alignment for integrating spatial transcriptomics data with PRECAST. Nat Commun 2023;14(1):296. https://doi.org/10.1038/s41467-023-35947-w.
19. Yuan Z. MENDER: fast and scalable tissue structure identification in spatial omics data. Nat Commun 2024;15(1):207. https://doi.org/10.1038/s41467-023-44367-9.
20. Li J, Chen S, Pan X, et al. Cell clustering for spatial transcriptomics data with graph neural networks. Nat Comput Sci 2022;2(6):399–408. https://doi.org/10.1038/s43588-022-00266-5.
21. Dong K, Zhang S. Deciphering spatial domains from spatially resolved transcriptomics with an adaptive graph attention auto-encoder. Nat Commun 2022;13(1):1739. https://doi.org/10.1038/s41467-022-29439-6.
22. Ren H, Walker BL, Cang Z, et al. Identifying multicellular spatiotemporal organization of cells with SpaceFlow. Nat Commun 2022;13(1):4076. https://doi.org/10.1038/s41467-022-31739-w.
23. Long Y, Ang KS, Li M, et al. Spatially informed clustering, integration, and deconvolution of spatial transcriptomics with GraphST. Nat Commun 2023;14(1):1155. https://doi.org/10.1038/s41467-023-36796-3.
24. Pham D, Tan X, Balderson B, et al. Robust mapping of spatiotemporal trajectories and cell–cell interactions in healthy and diseased tissues. Nat Commun 2023;14(1):7739. https://doi.org/10.1038/s41467-023-43120-6.
25. Xu C, Jin X, Wei S, et al. DeepST: identifying spatial domains in spatial transcriptomics by deep learning. Nucleic Acids Res 2022;50(22):e131–e131. https://doi.org/10.1093/nar/gkac901.

26. He S, Bhatt R, Brown C, et al. High-plex imaging of RNA and proteins at subcellular resolution in fixed tissue by spatial molecular imaging. Nat Biotechnol 2022;40(12):1794–1806. https://doi.org/10.1038/s41587-022-01483-z.
27. Dosovitskiy A, Beyer L, Kolesnikov A, et al. An Image is Worth 16x16 Words: Transformers for Image Recognition at Scale. arXiv preprint arXiv:201011929 2020;https://doi.org/10.48550/arXiv.2010.11929.
28. Kipf TN, Welling M. Semi-Supervised Classification with Graph Convolutional Networks. arXiv preprint arXiv:160902907 2016;https://doi.org/10.48550/arXiv.1609.02907.
29. Brauwers G, Frasincar F. A General Survey on Attention Mechanisms in Deep Learning. IEEE Trans Knowl Data Eng 2023;35(4):3279–3298. https://doi.org/10.1109/TKDE.2021.3126456.
30. Markey M, Kim J, Goldstein Z, et al. Spatially-resolved prediction of gene expression signatures in H&E whole slide images using additive multiple instance learning models. Mol Cancer Ther 2023;22(12_Supplement):B010–B010. https://doi.org/10.1158/1535-7163.TARG-23-B010.
31. Bergenstråhle L, He B, Bergenstråhle J, et al. Super-resolved spatial transcriptomics by deep data fusion. Nat Biotechnol 2022;40(4):476–479. https://doi.org/10.1038/s41587-021-01075-3.
32. Zeng Y, Wei Z, Yu W, et al. Spatial transcriptomics prediction from histology jointly through Transformer and graph neural networks. Brief Bioinform 2022;23(5):bbac297. https://doi.org/10.1093/bib/bbac297.
33. Covert I, Gala R, Wang T, et al. Predictive and robust gene selection for spatial transcriptomics. Nat Commun 2023;14(1):2091. https://doi.org/10.1038/s41467-023-37392-1.
34. Yu Z, Lu Y, Wang Y, et al. ZINB-Based Graph Embedding Autoencoder for Single-Cell RNA-Seq Interpretations. In: AAAI Conf Artif Intell, vol. 36; 2022. p. 4671–4679. https://doi.org/10.1609/aaai.v36i4.20392.
35. Kipf TN, Welling M. Variational Graph Auto-Encoders. arXiv preprint arXiv:161107308 2016;https://doi.org/10.48550/arXiv.1611.07308.
36. Tang M, Yang C, Li P. Graph Auto-Encoder Via Neighborhood Wasserstein Reconstruction. arXiv preprint arXiv:220209025 2022;https://doi.org/10.48550/arXiv.2202.09025.
37. Wolf FA, Angerer P, Theis FJ. SCANPY: large-scale single-cell gene expression data analysis. Genome Biol 2018;19:15. https://doi.org/10.1186/s13059-017-1382-0.
38. Li B, Zhang W, Guo C, et al. Benchmarking spatial and single-cell transcriptomics integration methods for transcript distribution prediction and cell type deconvolution. Nat Methods 2022;19(6):662–670. https://doi.org/10.1038/s41592-022-01480-9.
39. Cheng A, Hu G, Li WV. Benchmarking cell-type clustering methods for spatially resolved transcriptomics data. Brief Bioinform 2023;24(1):bbac475. https://doi.org/10.1093/bib/bbac475.
40. Zhu J, Shang L, Zhou X. SRTsim: spatial pattern preserving simulations for spatially resolved transcriptomics. Genome Biol 2023;24(1):39. https://doi.org/10.1186/s13059-023-02879-z.
41. Shang L, Zhou X. Spatially aware dimension reduction for spatial transcriptomics. Nat Commun 2022;13(1):7203. https://doi.org/10.1038/s41467-022-34879-1.
42. Maynard KR, Collado-Torres L, Weber LM, et al. Transcriptome-scale spatial gene expression in the human dorsolateral prefrontal cortex. Nat Neurosci 2021;24(3):425–436. https://doi.org/10.1038/s41593-020-00787-0.
43. Wolf FA, Hamey FK, Plass M, et al. PAGA: graph abstraction reconciles clustering with trajectory inference through a topology preserving map of single cells. Genome Biol 2019;20:59. https://doi.org/10.1186/s13059-019-1663-x.
44. Gao J, Zhang F, Hu K, et al. Hexagonal Convolutional Neural Network for Spatial Transcriptomics Classification. In: IEEE BIBM; 2022. p. 200–205. https://doi.org/10.1002/bies.201900221.
45. Avşar G, Pir P. A comparative performance evaluation of imputation methods in spatially resolved transcriptomics data. Mol Omics 2023;19(2):162–173. https://doi.org/10.1039/d2mo00266c.
46. Lopez R, Li B, Keren-Shaul H, et al. DestVI identifies continuums of cell types in spatial transcriptomics data. Nat Biotechnol 2022;40(9):1360–1369. https://doi.org/10.1038/s41587-022-01272-8.
47. Wang Y, Song B, Wang S, et al. Sprod for de-noising spatially resolved transcriptomics data based on position and image information. Nat Methods 2022;19(8):950–958. https://doi.org/10.1038/s41592-022-01560-w.
48. Arion D, Enwright JF, Gonzalez-Burgos G, et al. Differential gene expression between callosal and ipsilateral projection neurons in the monkey dorsolateral prefrontal and posterior parietal cortices. Cereb Cortex 2022;33(5):1581–1594. https://doi.org/10.1093/cercor/bhac157.
49. Arnsten AFT, Woo E, Yang S, et al. Unusual molecular regulation of dorsolateral prefrontal cortex layer III synapses increases vulnerability to genetic and environmental insults in schizophrenia. Biol Psychiatry 2022;92(6):480–490. https://doi.org/10.1016/j.biopsych.2022.02.003.
50. Mantri M, Scuderi GJ, Abedini-Nassab R, et al. Spatiotemporal single-cell RNA sequencing of developing chicken hearts identifies interplay between cellular differentiation and morphogenesis. Nat Commun 2021;12(1):1771. https://doi.org/10.1038/s41467-021-21892-z.
51. Haghverdi L, Büttner M, Wolf FA, et al. Diffusion pseudotime robustly reconstructs lineage branching. Nat Methods 2016;13(10):845–848. https://doi.org/10.1038/nmeth.3971.
52. Martinsen BJ. Reference guide to the stages of chick heart embryology. Dev Dyn 2005;233(4):1217–1237. https://doi.org/10.1002/dvdy.20468.
53. Lu ZQ, Sinha A, Sharma P, et al. Proteomic Analysis of Human Fetal Atria and Ventricle. J Proteome Res 2014;13(12):5869–5878. https://doi.org/10.1021/pr5007685.
54. Siegel RL, Miller KD, Fuchs HE, et al. Cancer statistics, 2022. CA Cancer J Clin 2022;72(1):7–33. https://doi.org/10.3322/caac.21708.
55. Piñero J, Queralt-Rosinach N, Bravo A, et al. DisGeNET: a discovery platform for the dynamical exploration of human diseases and their genes. Database (Oxford) 2015;2015:bav028. https://doi.org/10.1093/database/bav028.
56. Kang D, Yoon SE, Shin D, et al. Risk of non-Hodgkin lymphoma in breast cancer survivors: a nationwide cohort study. Blood Cancer J 2021;11(12):1–8. https://doi.org/10.1038/s41408-021-00595-0.
57. Berger E, Delpierre C, Hosnijeh FS, et al. Association between low-grade inflammation and Breast cancer and B-cell Myeloma and Non-Hodgkin Lymphoma: findings from two prospective cohorts. Sci Rep 2018;8(1):10805. https://doi.org/10.1038/s41598-018-29041-1.
58. Zhao H, Wu L, Yan G, et al. Inflammation and tumor progression: signaling pathways and targeted intervention. Signal Transduct Target Ther 2021;6(1):1–46. https://doi.org/10.1038/s41392-021-00658-5.
59. McAndrew NP, Bottalico L, Mesaros C, et al. Effects of systemic inflammation on relapse in early breast cancer. NPJ Breast Cancer 2021;7(1):1–10. https://doi.org/10.1038/s41523-020-00212-6.
60. Han H, Shim H, Shin D, et al. TRRUST: a reference database of human transcriptional regulatory interactions. Sci Rep 2015;5(1):11432. https://doi.org/10.1038/srep11432.
61. Gao Y, Gan K, Liu K, et al. SP1 Expression and the Clinicopathological Features of Tumors: A Meta-Analysis and Bioin-

formatics Analysis. Pathol Oncol Res 2021;27:581998. https://doi.org/10.3389/pore.2021.581998.
62. Wang W, Nag SA, Zhang R. Targeting the NFκB signaling pathways for breast cancer prevention and therapy. Curr Med Chem 2015;22(2):264–289. https://doi.org/10.2174/0929867321666141106124315.
63. Kanzaki H, Chatterjee A, Hossein Nejad Ariani H, et al. Disabling the nuclear translocalization of RelA/NF-κB by a small molecule inhibits triple-negative breast cancer growth. Breast Cancer (Dove Med Press) 2021;13:419–430. https://doi.org/10.2147/BCTT.S310231.
64. Kim GC, Kwon HK, Lee CG, et al. Upregulation of Ets1 expression by NFATc2 and NFKB1/RELA promotes breast cancer cell invasiveness. Oncogenesis 2018;7(11):1–15. https://doi.org/10.1038/s41389-018-0101-3.
65. Jeong YJ, Oh HK, Choi HR. Methylation of the RELA gene is associated with expression of NF-κB1 in response to TNF-α in breast cancer. Molecules 2019;24(15):2834. https://doi.org/10.3390/molecules24152834.
66. Wellenstein MD, Coffelt SB, Duits DEM, et al. Loss of p53 triggers WNT-dependent systemic inflammation to drive breast cancer metastasis. Nature 2019;572(7770):538–542. https://doi.org/10.1038/s41586-019-1450-6.
67. Mincarelli L, Uzun V, Wright D, et al. Single-cell gene and isoform expression analysis reveals signatures of ageing in haematopoietic stem and progenitor cells. Commun Biol 2023;6(1):1–11. https://doi.org/10.1038/s42003-023-04936-6.
68. Zhang H, Ding C, Li Y, et al. Data mining-based study of collagen type III alpha 1 (COL3A1) prognostic value and immune exploration in pan-cancer. Bioengineered 2021;12(1):3634–3646. https://doi.org/10.1080/21655979.2021.1949838.
69. Ren J, Da J, Hu N. Identification of COL1A1 associated with immune infiltration in brain lower grade glioma. PLoS One 2022;17(7):e0269533. https://doi.org/10.1371/journal.pone.0269533.
70. Moreno CS. SOX4: The Unappreciated Oncogene. Semin Cancer Biol 2020;67:57–64. https://doi.org/10.1016/j.semcancer.2019.08.027.
71. Tang M, Liu P, Wu X, et al. COL3A1 and Its Related Molecules as Potential Biomarkers in the Development of Human Ewing's Sarcoma. Biomed Res Int 2021;2021(1):7453500. https://doi.org/10.1155/2021/7453500.
72. Geng Q, Shen Z, Li L, et al. COL1A1 is a prognostic biomarker and correlated with immune infiltrates in lung cancer. Peerj 2021;9:e11145. https://doi.org/10.7717/peerj.11145.
73. Walter RFH, Mairinger FD, Werner R, et al. SOX4, SOX11 and PAX6 mRNA expression was identified as a (prognostic) marker for the aggressiveness of neuroendocrine tumors of the lung by using next-generation expression analysis (NanoString). Future Oncol 2015;11(7):1027–1036. https://doi.org/10.2217/fon.15.18.
74. Srivastava M, Khurana P, Sugadev R. Lung Cancer Signature Biomarkers: tissue specific semantic similarity based clustering of Digital Differential Display (DDD) data. Bmc Res Notes 2012;5:617. https://doi.org/10.1186/1756-0500-5-617.
75. Wang Z, Li Z, Zhou K, et al. Deciphering cell lineage specification of human lung adenocarcinoma with single-cell RNA sequencing. Nat Commun 2021;12(1):6500. https://doi.org/10.1038/s41467-021-26770-2.
76. Zuo W, Rostami MR, Shenoy SA, et al. Cell-specific expression of lung disease risk-related genes in the human small airway epithelium. Respir Res 2020;21(1):200. https://doi.org/10.1186/s12931-020-01442-9.
77. Janiszewska M, Primi MC, Izard T. Cell adhesion in cancer: Beyond the migration of single cells. J Biol Chem 2020;295(8):2495–2505. https://doi.org/10.1074/jbc.REV119.007759.
78. Millar FR, Janes SM, Giangreco A. Epithelial cell migration as a potential therapeutic target in early lung cancer. Eur Respir Rev 2017;26(143). https://doi.org/10.1183/16000617.0069-2016.
79. Liu Z, Wu D, Zhai W, et al. SONAR enables cell type deconvolution with spatially weighted Poisson-Gamma model for spatial transcriptomics. Nat Commun 2023;14(1):4727. https://doi.org/10.1038/s41467-023-40458-9.
80. Deng Y, Bartosovic M, Kukanja P, et al. Spatial-CUT&Tag: Spatially resolved chromatin modification profiling at the cellular level. Science 2022;375(6581):681–686. https://doi.org/10.1126/science.abg7216.
81. Zhang D, Deng Y, Kukanja P, et al. Spatial epigenome–transcriptome co-profiling of mammalian tissues. Nature 2023;616(7955):113–122. https://doi.org/10.1038/s41586-023-05795-1.
82. Xie Y, Ruan F, Li Y, et al. Spatial chromatin accessibility sequencing resolves high-order spatial interactions of epigenomic markers. Elife 2024;12:RP87868. https://doi.org/10.7554/eLife.87868.4.
83. Deng Y, Bartosovic M, Ma S, et al. Spatial profiling of chromatin accessibility in mouse and human tissues. Nature 2022;609(7926):375–383. https://doi.org/10.1038/s41586-022-05094-1.
84. Dong Y, Chawla NV, Swami A. metapath2vec: Scalable Representation Learning for Heterogeneous Networks. In: ACM SIGKDD International Conference on Knowledge Discovery & Data Mining; 2017. p. 135–144. https://doi.org/10.1145/3097983.3098036.
85. Li X, Chen W, Chen Y, et al. Network embedding-based representation learning for single cell RNA-seq data. Nucleic Acids Res 2017;45(19):e166–e166. https://doi.org/10.1093/nar/gkx750.
86. Guo T, Chen Y, Shi M, et al. Integration of single cell data by disentangled representation learning. Nucleic Acids Res 2022;50(2):e8–e8. https://doi.org/10.1093/nar/gkab978.
87. Wu G, Li X, Guo W, et al. JEBIN: analyzing gene co-expressions across multiple datasets by joint network embedding. Brief Bioinform 2022;23(2):bbab603. https://doi.org/10.1093/bib/bbab603.

Click here to access/download;Manuscript;main.tex

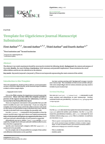

GigaScience, 2023, 1–13

doi: xx.xxxx/xxxx
Manuscript in Preparation
Technical Note

TECHNICAL NOTE

stMMR: accurate and robust spatial domain identification from spatially resolved transcriptomics with multi-modal feature representation

Daoliang Zhang[1,†], Na Yu[1,†], Zhiyuan Yuan[2,†], Wenrui Li[3], Xue Sun[1], Qi Zou[1], Xiangyu Li[4], Zhiping Liu[1], Wei Zhang[1,*] and Rui Gao[1,*]

[1]Center of Intelligent Medicine, School of Control Science and Engineering, Shandong University, Jinan 250061, China and [2]Institute of Science and Technology for Brain-Inspired Intelligence, Center for Medical Research and Innovation, Shanghai Pudong Hospital, Fudan University Pudong Medical Center, Fudan University, Shanghai 200433, China and [3]MOE Key Lab of Bioinformatics and Bioinformatics Division of BNRIST, Department of Automation, Tsinghua University, Beijing 100084, China and [4]School of Software Engineering, Beijing Jiaotong University, Beijing 100044, China

[*] Correspondence address. Rui Gao, No. 17923, Jingshi Road, Lixia District, Jinan, Shandong, China. E-mail: gaorui@sdu.edu.cn; Wei Zhang, No. 17923, Jingshi Road, Lixia District, Jinan, Shandong, China. E-mail: zw@sdu.edu.cn
[†] These authors contributed equally as the first authors.

Abstract

Background: Deciphering spatial domains using spatially resolved transcriptomics (SRT) is of great value for the characterizing and understanding of tissue architecture. However, the inherent heterogeneity and varying spatial resolutions present challenges in the joint analysis of multi-modal SRT data. **Results:** We introduce a multi-modal geometric deep learning method, named stMMR, to effectively integrate gene expression, spatial location and histological information for accurate identifying spatial domains from SRT data. stMMR uses graph convolutional networks (GCN) and self-attention module for deep embedding of features within unimodal and incorporates similarity contrastive learning for integrating features across modalities. **Conclusions:** Comprehensive benchmark analysis on various types of spatial data shows superior performance of stMMR in multiple analyses, including spatial domain identification, pseudo-spatiotemporal analysis and domain-specific gene discovery. In chicken heart development, stMMR reconstruct the spatiotemporal lineage structures indicating accurate developmental sequence. In breast cancer and lung cancer, stMMR clearly delineated the tumor microenvironment and identified marker genes associated with diagnosis and prognosis. Overall, stMMR is capable of effectively utilizing the multi-modal information of various SRT data to explore and characterize tissue architectures of homeostasis, development and tumor.

Key words: spatially resolved transcriptomics; domain identification; multi-modal integration; geometric deep learning; similarity contrastive learning

Introduction

The advancement in spatially resolved transcriptomics (SRT) technologies has opened new avenues for a deeper understanding of the spatial architecture and functionality of tissues. Currently, many SRT technologies have been developed, such as imaging-based and sequencing-based methods [1, 2, 3, 4, 5, 6, 7]. Among these, techniques such as 10x Genomics Visium not only provide the spatial

Compiled on: October 22, 2024.
Draft manuscript prepared by the author.

location and gene expression data for each spot but also acquire high-resolution hematoxylin and eosin (H&E) stained histology images of the tissue section, revealing richer information about the tissue organization. These technological advancements offer new insights into characterization of tissue architecture, enabling a more comprehensive understanding of tissue development and disease pathogenesis [7, 8, 9].

For SRT technologies capable of providing both gene expression data and histology images, the information from these different modalities reflects the structural information of tissues at various levels. Gene expression profiles reflect the difference of cell state between spots [10]. Spatial location information provides the precise location of each spot. Histological images display morphological features of cells, such as size and shape [11]. Although each of these modalities has its own strength, they complement each other, together forming a more comprehensive picture of tissue architecture. For instance, changes in gene expression are reflected not only at the molecular level but may also manifest in histological images as morphological alterations[12]. Furthermore, the issues of sparsity and dropout in SRT data can be effectively addressed through integrating histological image data [13]. By leveraging the interdependence between gene expression and morphological features, as well as the similarity in gene expression patterns among adjacent spots, we can enhance spatial signals and characterize tissue structure.

However, the joint representation of multi-modal features in SRT is challenging. Firstly, these different modalities inherently possess significant heterogeneity. For instance, transcriptomic data are typically high-dimensional, quantified gene expression information, reflecting the gene activity in different spots or cells. In contrast, histology images are two-dimensional visual data depicting the morphological and structural information of cells at different spots. This fundamental difference makes the direct fusion of these two types of modalities difficult. Secondly, the disparity in data scale and resolution is also a crucial issue. Transcriptomic data reveals unique patterns of gene expression within spots or cells from a microscopic perspective. Conversely, histology images provide more macroscopic information on organization and morphology. This difference in scale complicates the establishment of spatial correspondence. Therefore, there is an urgent need for methods that can effectively integrate multi-modal features.

Recently, a variety of cutting-edge computational methods have been developed to effectively address the challenge of joint representation of multi-modal SRT data. Specifically, BASS, BayesSpace and Giotto leverage spatial neighborhood information for enhancing the resolution of SRT data [14, 15, 16]. CellCharter and PRECAST incorporate spatial contexts to correct batch effect for a better domain identification [17, 18]. MENDER is a recently proposed multi-range cell context decipherer for ultra-fast tissue structure identification [19]. CCST, STAGATE, SpaceFlow and GraphST utilize Graph Neural Networks (GNN) to integrate gene expression data with spatial information, achieving effective clustering of spots [20, 21, 22, 23]. However, these methods do not employ histology images, failing to fully enhance the interpretability of gene expression data through these images. In contrast, recent pioneering studies like stLearn and DeepST have shown more significant progress [24, 25]. These methods effectively integrate gene expression data with spatial neighborhood information and morphological features extracted from histology images, demonstrating a stronger potential for application. Despite these methods demonstrating capability in processing multi-modal information in SRT data, they give less consideration to the complex global spot similarity across distinctive spatial multi-modal features. This limitation impedes their ability to accurately characterize spatial patterns and discover functional biological contexts in tissue.

To achieve precise identification of spatial domains, we introduce stMMR, a geometric deep learning method for effective representing multi-modal information in SRT data. stMMR utilizes spatial location information as a bridge to establish adjacency relationships between spots. It encodes gene expression data and morphological features extracted from histological images using GCN. stMMR proposed a novel strategy to achieve joint learning of intra-modal and inter-modal features. Within a certain modality, stMMR employs self-attention mechanisms to dynamically learn the complex relationships of different spots. For integrating cross-modal information, stMMR innovatively utilizes similarity contrastive learning along with the reconstruction of gene expression features and adjacency information. This enhances the ability of stMMR to recover information and provides denoising capabilities. We conducted comprehensive tests on different SRT datasets, including samples profiled by 10x Visium, NanoString technology and Spatial Transcriptomics (ST) technology. stMMR outperforms SOTA techniques in terms of domain identification, pseudo-spatiotemporal analysis and domain-specific gene discovery. The experimental results on breast cancer and lung cancer [26] demonstrated that stMMR accurately identifies tumor edges and tumor-infiltrating regions, proving its potential value in clinical research. Overall, the stMMR exhibits exceptional capability in the multi-modal feature representation of SRT, providing a powerful new tool for accurate and robust domain identification.

Methods

Overview of stMMR

The multi-modal joint representation process of stMMR primarily consists of the following three steps: multi-modal feature embedding, feature fusion and feature reconstruction. The overall workflow of stMMR is illustrated in Fig. 1 and the detailed implementation are introduced in Supplementary Section 1.

Multi-modal feature embedding

The stMMR initially performs embedding on gene expression, spatial location, and histology image information. For gene expression data, $G \in \mathbb{R}^{N\times P}$ represents the normalized gene expression matrix, where N is the number of spots and P is the number of identified high variance genes. For histological images, we use a pre-trained Vision Transformer (ViT) model [27] to extract the image features matrix $H \in \mathbb{R}^{N\times M}$, where M is the output dimension. To encode the spatial location information, we construct an undirected weighted graph to present SRT data, and the adjacency matrix A is defined as:

$$A_{ij} = exp\left(-\frac{d(i,j)^2}{2l^2}\right) \quad (1)$$

where $d(i,j)$ represents the Euclidean distance between spots i and j, and l is used to control the relationship between weight and distance. A larger value of l implies a faster decay of weight with increasing distance.

Next, we employ a two-layer GCN encoder for message passing and aggregation of image features and gene expression features [28]:

$$E^{(k)} = \tilde{D}^{-\frac{1}{2}}\tilde{A}\tilde{D}^{-\frac{1}{2}}E^{(k-1)}W^{(k-1)} \quad (2)$$

where $E^{(k)}$ and $E^{(k-1)}$ represent the input and output of GCN encoder. $E^{(0)}$ can be the image features H or gene expression features G. $\tilde{A} = A + I$ denotes the symmetrically normalized adjacency matrix, where I is the identity matrix. $\tilde{D}$ and $W^{(k-1)}$ are the weighted degree matrix and trainable parameter respectively. The visual features and gene expression features obtained after the encoder are denoted as E_H and E_G.

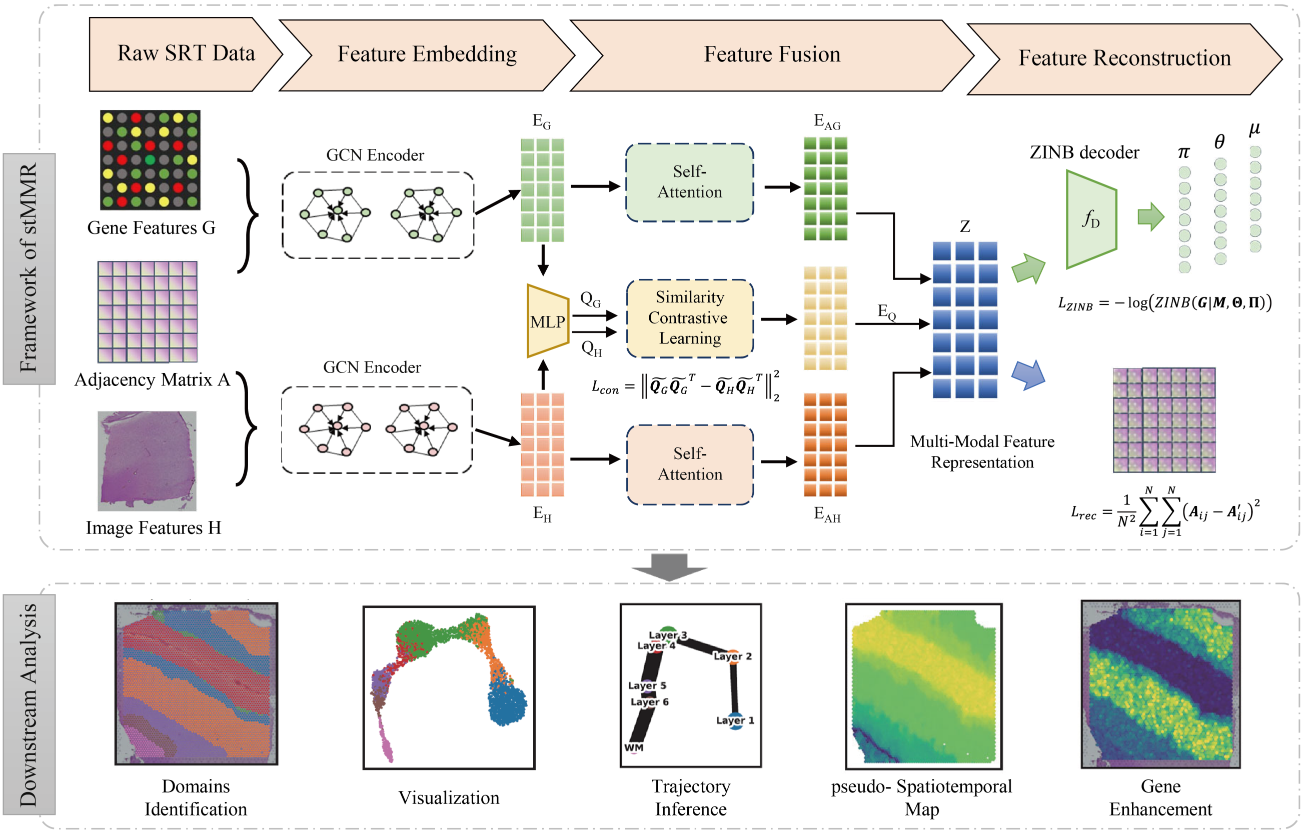

Figure 1. Schematic overview of stMMR for the joint representation of features from different modalities. Gene expression and histology image information are embedded using GCN module based on adjacent matrix. Then, the relationships between different modalities are captured through similarity contrast learning, followed by feature fusion. Finally, the original features are reconstructed from the multi-modal feature representation. This representation can be used for downstream analysis directly.

Feature fusion

We propose a novel strategy for multi-modal information aggregation. Biological structures often exhibit relationships between distant regions that cannot be captured by local spatial dependencies alone. For instance, in complex tissues such as the brain or cancerous tissues, cells in non-adjacent spatial domains can share similar gene expression patterns or morphological characteristics. Therefore, stMMR uses a normalized attention module to learn the global relationships between spots in a single modality, as shown in Eq.3:

$$E_A = softmax\left(\frac{E \cdot E^T}{\sqrt{d}}\right) \cdot E \tag{3}$$

where E represents the imaging features E_H or transcriptomics features E_G from the previous step. New features obtained through the attention module are E_{AH} and E_{AG}. Notably, a nonlinear activation function and Euclidean distance matrix d normalize the weights, preventing the local optima issue from oversized weights for certain spots [29].

stMMR uses contrastive learning for cross-modal feature fusion, highlighting the consistency between modalities like morphology and gene expression, which have both similarities and complementary relationships [30, 31, 32]. It maps the latent features E_H and E_G through two fully connected neural networks to obtain hierarchical representations Q_H and Q_G, as shown in Eq.4:

$$Q = Relu(W_Q E + b_Q) \tag{4}$$

where E represents the morphological features E_H or gene expression features E_G, and Q corresponds to Q_H or Q_G. W_Q and b_Q are the parameters of the fully connected network.

After obtaining low-dimensional features Q_H and Q_G for the two modalities, a fully connected neural network is employed to fuse them, as shown in Eq.5:

$$E_Q = W_E \cdot concat(Q_G, Q_H) + b_E \tag{5}$$

where W_E and b_E are the parameters of the fully connected network.

To enhance the consistency between Q_H and Q_G, we use a constraint as shown in Eq.6, replacing the loss of traditional contrastive learning:

$$L_{con} = \left\| \widetilde{Q_G}\widetilde{Q_G}^T - \widetilde{Q_H}\widetilde{Q_H}^T \right\|_2^2 \tag{6}$$

where $\widetilde{Q_G}$ and $\widetilde{Q_G}$ are the normalization matrices of Q_G and Q_H, respectively.

Finally, we further integrate modality specific features E_{AH} and E_{AG} obtained from Eq.3 with the cross-modality features E_Q obtained from Eq.5 to get the multi-modal feature representation Z, as shown in the following equation:

$$Z = \alpha E_Q + \beta E_{AH} + \gamma E_{AG} \tag{7}$$

where α, β, and γ are hyperparameters for adjusting the importance of features.

Feature reconstruction

stMMR adopts the zero-inflated negative binomial (ZINB) decoder [33, 34] to reconstruct gene expression information and the adjacency matrix is estimated directly using the concept of a graph

auto-encoder [35, 36]:

$$L_{ZINB} = -\log\left(ZINB(G|M,\Theta,\Pi)\right) \tag{8}$$

$$A^{'} = Sigmoid\left(\frac{Z \cdot Z^T}{\|Z\|_2 \cdot \|Z^T\|_2}\right) \tag{9}$$

where M, Θ, and Π are the mean, dispersion, and dropout probability of the output from network respectively.

Subsequently, the regularization loss between the reconstructed matrix and the adjacency matrix can be computed:

$$L_{rec} = \frac{1}{N^2}\sum_{i=1}^{N}\sum_{j=1}^{N}\left(A_{ij} - A_{ij}^{'}\right)^2 \tag{10}$$

Objective function

Finally, we integrated Eq.6, Eq.8, and Eq.10 to formulate the final objective function:

$$L = a * L_{con} + b * L_{ZINB} + c * L_{rec} \tag{11}$$

In this equation, a, b, and c are weight for the different loss terms.

For detailed information on the training process and parameter settings, please refer to the Supplementary Section 1.

Benchmark Methods

To demonstrate the effectiveness of the multi-modal feature representation in SRT data, we selected 7 SOTA methods for benchmarking comparison. These methods include SCANPY [37], which utilizes only gene expression data; CCST, STAGATE, GraphST, and SpaceFlow, which employ both gene expression and spatial location information [20, 21, 22, 23]; stLearn and DeepST, which incorporate all three modalities [24, 25]. Methods that have already been compared in previous works are not included in our analysis [38, 39, 40].

Results

stMMR enhances detection of stratified architectural patterns in human dorsolateral prefrontal cortex (DLPFC) tissue

The spatial structure of the brain is closely related to its function, particularly evident in the layered organization of the human brain cortex [41]. To explore the spatial structure arrangement of brain , we collected a 10x Visium dataset containing 12 dorsolateral prefrontal cortex (DLPFC) sections [42]. The histology image and manually annotated layers are illustrated in Fig. 2A.

We initially compared the Adjusted Rand Index (ARI) levels of various methods across 12 slices of the DLPFC dataset (Fig. 2B). The result reveals that stMMR outperformed other methods, achieving the highest ARI and the smallest variance compared to manual annotations. Notably, the results from STAGATE, CCST, and SpaceFlow show differences in the ARI across different slices, indicating that these methods are more sensitive to the domain patterns. Scanpy uses only gene expression information and shows the poorest performance. Methods like stlearn and DeepST, which integrate histological information, are outperformed by GraphST and STAGATE. This underperformance might stem from insufficient integration of transcriptomic and imaging data.

Next, we conducted a detailed analysis for each slice (Fig. 2C and Supplementary Fig. S1). To demonstrate the results, we used slice 151509 as an example (Fig. 2C-E). The results shows that DeepST struggle with rough segmentation between layers. CCST, SpaceFlow, and stLearn have issues with erroneous region identification. Although GraphST and STAGATE accurately discern the arrangement of different regions, these methods exhibit biases in identifying the boundaries between distinct domains. In this specific case, stMMR demonstrates exceptional domain identification results. We further utilize UMAP for low-dimensional visualization analysis of the results obtained from different methods (Fig. 2D), to verify whether the embeddings can accurately encompass information on regional arrangement and boundaries. The analysis reveals that techniques such as stMMR, CCST, STAGATE, and stLearn effectively separate different domains. In contrast, GraphST, SpaceFlow and DeepST exhibit noticeable issues in layer boundaries. For instance, the boundaries between layers 2, 3, and 4 are confused.

Further, we conducted a detailed trajectory inference using the PAGA algorithm [43] for these methods (Fig. 2E). The PAGA graphs indicates that stMMR, STAGATE, CCST, and stLearn performs well in predicting trajectory between adjacent layers. The other methods display confused results in this analysis.

Combining the insights from these various analyses, it is evident that stMMR remarkably effective in domain identification and trajectory inference. These results adequately demonstrate effective capability of stMMR in integrating transcriptomic and histological data.

stMMR enhances spatial gene expression profiling and structural characterization

In SRT, the analysis of domain-specific genes holds significant importance. However, identifying domain-specific genes which have relationships with histological structures is challenging. This is primarily due to the presence of substantial noise in the gene expression profiles generated by SRT techniques, such as the dropout event [44, 45, 46]. To validate whether stMMR can enhance gene expression data through histological information, we analyzed domain-specific genes identified using the original gene expression profile and the profile reconstructed through the ZINB decoder [47].

Using both original and reconstructed gene expression data, genes such as AQP4 and HPCAL1 are recognized as layer-specific genes. These genes are enriched in multiple layers and have been confirmed through multiplex single-molecule fluorescent in situ hybridization [42]. However, employing reconstructed gene expression data facilitates the identification of new domain-specific genes. For instance, with the enhancement of stMMR, CACNA2D2 and ADCYAP1 can be identified as domain-specific genes in layer 3. Previous research has found that in layer 3 of primates, the CACNA2D2 gene exhibits differential expression and is closely associated with several biological pathways, including calcium signaling and synaptic long-term depression [48]. ADCYAP1 has also been proved to be a domain-specific gene in former study [49]. This suggests that the expression patterns after stMMR enhancement are more consistent with known neurobiological functions and pathological states.

We also conducted a more detailed analysis by combining gene expression levels with their spatial locations (Fig. 3). We found that after enhancement with stMMR, more distinct expression patterns of domain-specific genes can be observed. Specifically, Fig. 3A demonstrates a clear spatial representation of domain-specific marker genes (ADAYAP1, CACNA2D2, CALB1, MARC1, MB and LPL) after data enhancement. In the original data, the expression pattern of genes such as ADCYAP1, CACNA2D2 and MB are sparse, and the boundaries in spatial regions are blurred, making it difficult to discern a clear expression pattern (Fig. 3A and B). However, after enhancement with stMMR, we can observe that CACNA2D2 and CALB1 exhibit much clearer expression patterns in layers 2 and 3. Additionally, the enrichment of LPL in the layer 1 become more pro-

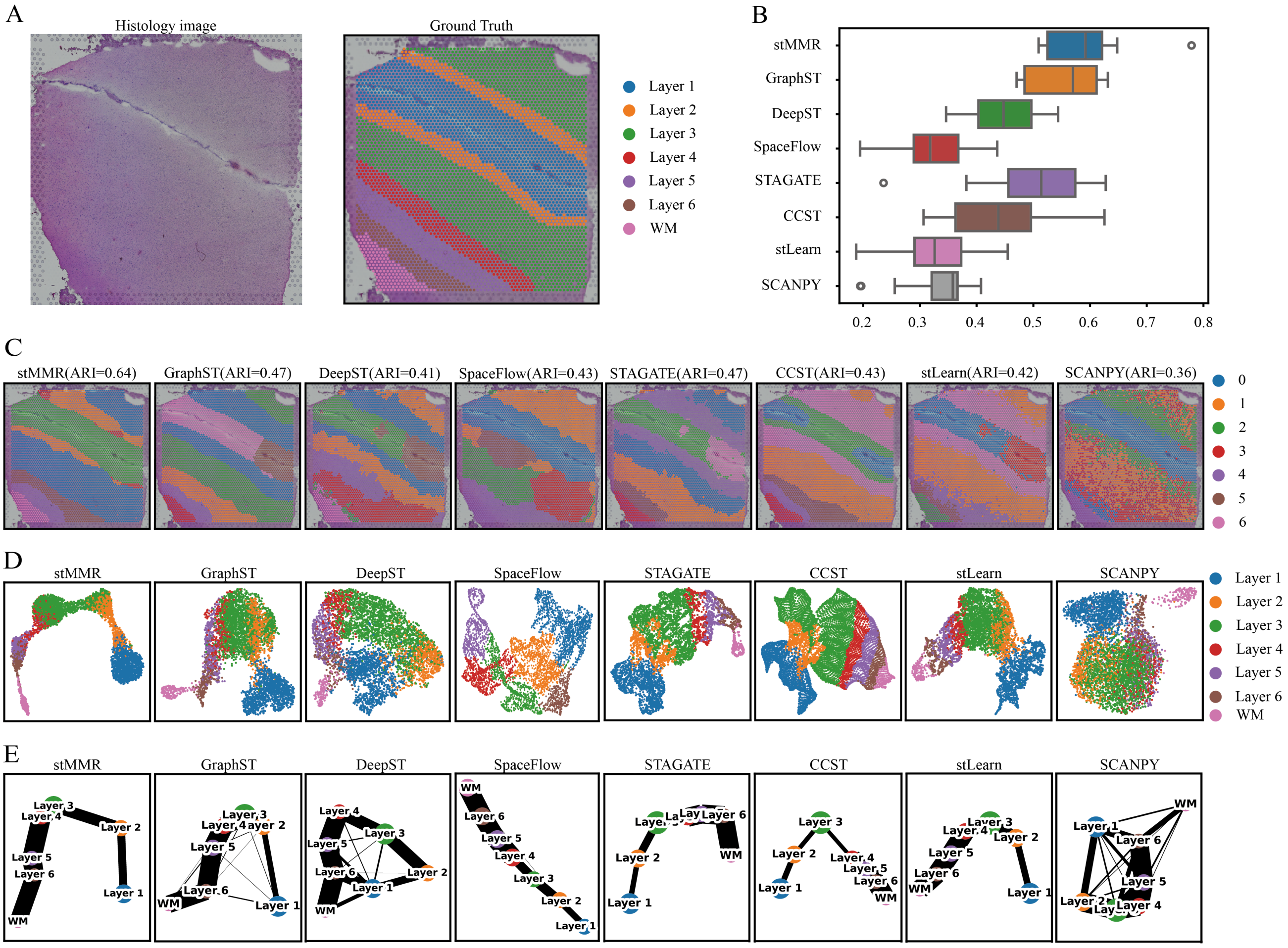

Figure 2. Performance comparisons of different methods on DLPFC datasets. (A) The histology image and manually annotated brain regions of slice 151509. (B) The overall performance of 8 different methods across 12 slices. (C) The domain recognition results on slice 151509. (D) The UMAP visualization results of the embeddings from 8 different methods on slice 151509. (E) The inferenced trajectories on slice 151509.

nounced (Fig. 3A and C). These results reflect that stMMR not only improves the spatial resolution of gene expression patterns using histological information but also enhances our understanding of the subtle differences in gene expression across different regions of the brain.

stMMR deciphers evolving cell lineage structures in chicken heart ST dataset

Analyzing temporal SRT data can reveal the dynamic domain changes during the development of tissue organs. We collected the chicken heart SRT dataset to further investigate the effectiveness of stMMR in the integrated representation of multi-modal features [50]. This dataset includes 12 tissue slices, collected on day 4 (5 slices), day 7 (4 slices), day 10 (2 slices), and day 14 (1 slices), documenting four key stages of the Hamburger-Hamilton ventricular developmental stages [50]. We selected SpaceFlow as the baseline because it was specifically designed to capture spatiotemporal dynamics.

We annotated the slices of different developmental stages using labels provided by the original research (Fig. 4A) [50]. Subsequently, we employed the embeddings from stMMR and SpaceFlow to identify domains of chicken heart across these four distinct stages. Fig. 4B indicate that the regions detected by stMMR largely coincide with manual annotation. For instance, major regions of the chicken heart, such as atrial cells and the inter-ventricular septum, are accurately identified. Notably, stMMR also detects domains that are hard to identified (Fig. 4B). For example, in the data from days 7, 10, and 14, the epicardium, a thin layer surrounding the outer side of the chicken heart, is clearly identified by stMMR. Although there are some instances of misclassification in the characterization of spot features using stMMR in a few regions, the identification of the epicardium is quite clear (Fig. 4B).

Next, we adopted a method similar to previous study to analyze the pseudo-spatiotemporal map (pSM) [22]. In brief, we mapped the spot features obtained through stMMR and SpaceFlow on the pseudo-temporal axis [22, 37, 51]. These points reflect the relative positions of cells in their developmental trajectory or functional state. As clearly visible in Fig. 4C, within the D7 to D14, the valve structures can be distinctly identified through the pSM values. Moreover, the representation of the myocardium in ventricles, as indicated by the pSM values, appears more uniform (yellow area) compared to the regional segmentation results in Fig. 4B. According to related research [52], the endocardium, the inner layer of the heart, is one of the early events in cardiac formation. The endocardial tubes are fundamental to cardiac development, eventually merging to form the primitive heart tube. As the heart tube forms, myocardial development commences, followed closely by the development of the atria. In our analysis, we observe that the myocardium in ventricles (yellow area in Fig. 4C) consistently shows higher pSM values compared to other areas in the same stage, indicating a later pseudo-temporal ordering of the ventricular myocardium [22]. Additionally, the pseudo-temporal ordering of the

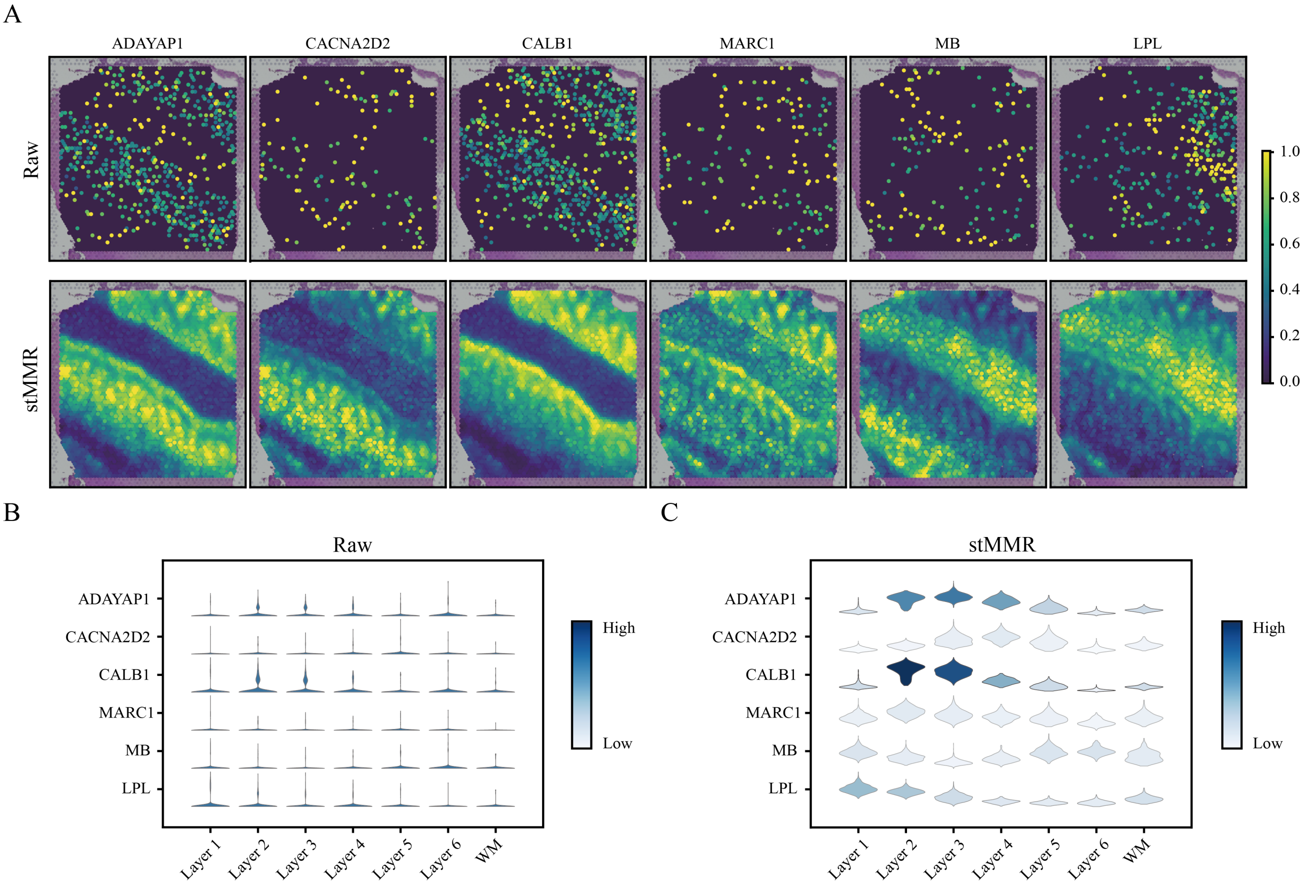

Figure 3. stMMR enhances spatial gene expression profiles and spatial structural characterization. (A) Spatial representation of layer-specific marker genes before and after data enhancement. (B) Gene expression level before and after data enhancement.

atria (marked in teal) follows that of the valves, suggesting that the development of the atria occurs after the valves. Therefore, the pSM derived from stMMR accurately displays the developmental sequence of the chicken heart. We further identified domain-specific genes through differential expression analysis across regions. For instance, we observed that MYH7 is highly specifically expressed in the Atria. This finding aligns with previous reports on the analysis of Atria and Ventricles specific proteins [53].

stMMR accurate identifies tumor region in human breast cancer

Breast cancer is a major type of cancer worldwide [54]. We collected a human breast cancer dataset from 10x Visium platform to conduct an in-depth analysis of the microenvironment in breast cancer (Fig. 5A).

First, we applied different methods for domain identification. From the results presented in Fig. 5B, it is observed that stMMR shows the most outstanding performance in category labeling. In terms of regional continuity, stMMR also demonstrates superior performance among different methods. Taking the IDC_5 area in the upper left corner as an example, this area occupies a significant portion in invasive ductal carcinoma, with a notable increase in cancer cells compared to normal tissue or non-tumorous areas [23]. However, only stMMR accurately identified the entire IDC_5 area, demonstrating higher precision compared to other methods. Additionally, stMMR also exhibits higher continuity in predicting the Tumor_edge area, whereas the results of other methods appear more dispersed in this aspect.

Next, we conducted a comprehensive analysis of domain-specific genes between merged tumor and normal regions (Supplementary Section 2.3). We utilized the DisGeNET to delve into the domain-specific genes of tumor regions [55]. Our analysis revealed that these domain-specific genes are enriched in several breast cancer related terms such as non-Hodgkin lymphoma and inflammation (Fig. 5C). Studies have shown that the development of breast cancer significantly increases the risk of non-Hodgkin lymphoma, particularly follicular lymphoma and mature T/NK cell lymphomas [56]. Numerous studies also have indicated that inflammation plays a regulatory role in the development of cancer and its response to treatment [57, 58, 59]. To further validate our research findings, we conducted an analysis of the transcriptional regulatory network using TRRUST [60]. The results indicated that multiple top-ranked terms are closely associated with breast cancer (Fig. 5C). For instance, the key regulatory factors (SP1, NFKB1, RELA and TP53) from the top four terms have been confirmed to play pivotal roles in the development and progression of breast cancer [61, 62, 63, 64, 65, 66].

stMMR dissects cell type differences in a lung cancer SRT dataset based on NanoString technology

To further validate the generalization ability and applicability of stMMR, we applied stMMR to the single-cell SRT dataset generated by NanoString CosMx SMI. This dataset comprises lung cancer tissue samples from 20 fields of view (FOVs) [26], and covering eight major cell types (Fig. 6A and E)

We employed the benchmarking methods to identify spatial domains within 20 FOVs, as shown in Fig. 6B, D-F. Fig. 6B revealed that stMMR closely aligns with the original study in detecting the spatial distribution of cell types, particularly in identifying tumor cells. In the overall analysis of the 20 sections, the performance

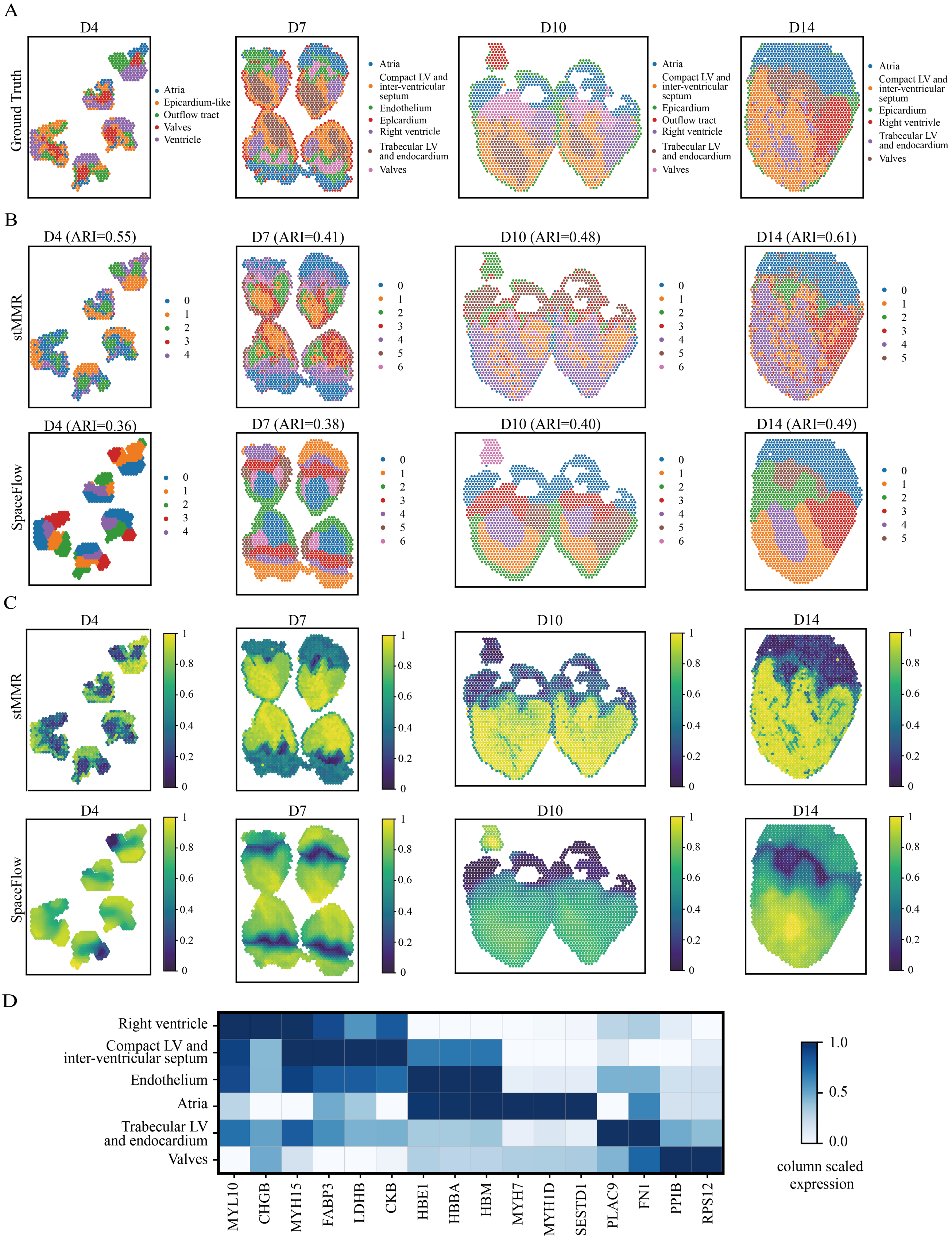

Figure 4. stMMR reveals cell lineage structures during chicken heart development. (A) The ground truth label provided by the original data. (B) The domains recognized by stMMR and SpaceFlow. (C) The plots of pSM value from stMMR and SpaceFlow for illustrating pseudo-temporal developmental trajectory. (D) The differentially expressed marker genes discovered by stMMR.

of stMMR is superior to other methods (Fig. 6D). Furthermore, we conducted a cell type-specific gene analysis based on the cell annotations in one slice. We observe that different cells exhibit unique expression patterns (Fig. 6C). For instance, Igkc transcripts,

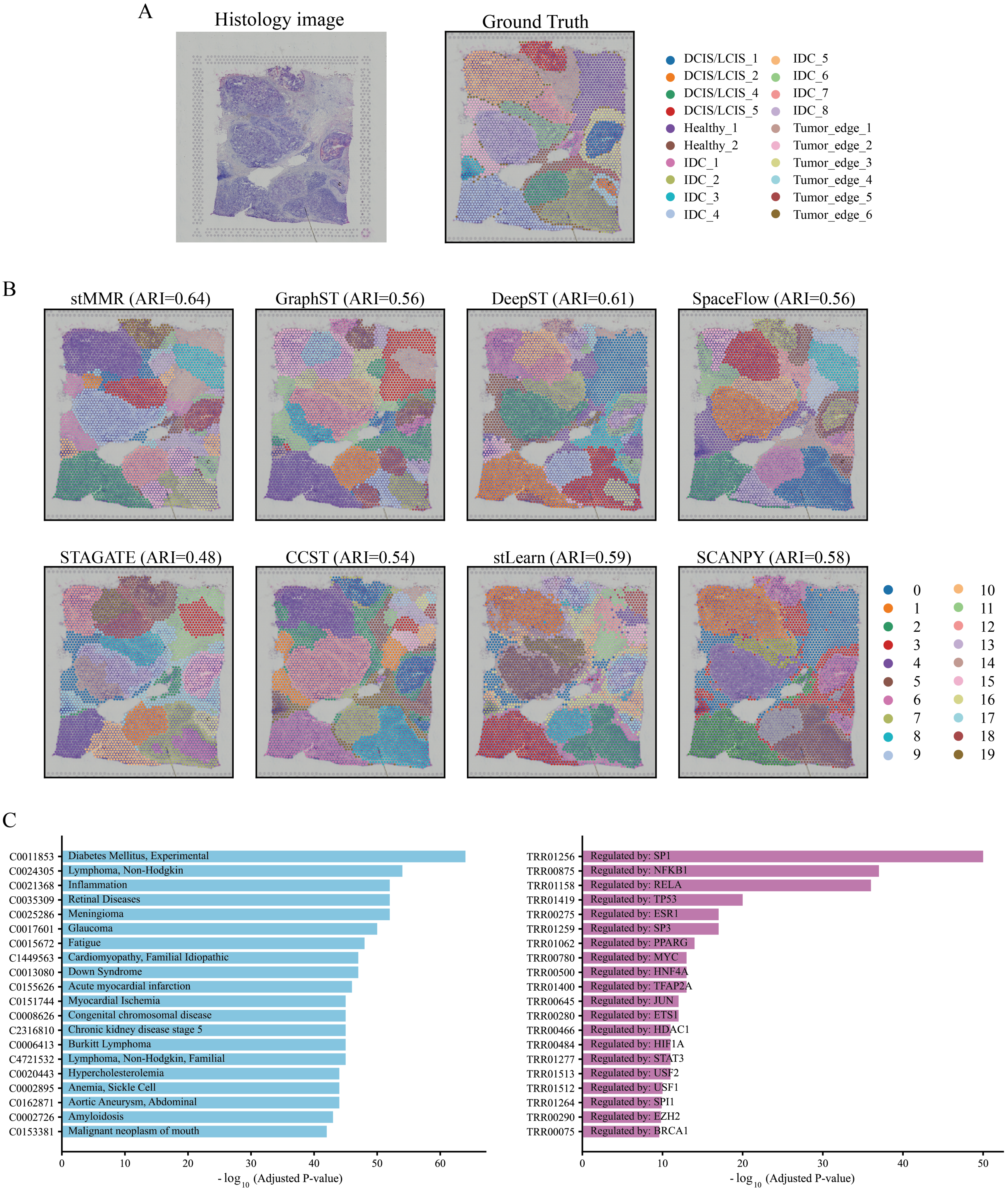

Figure 5. stMMR identifies tumor region in human breast cancer dataset. (A) The H&E images and the manually annotated regions. (B) The annotation results from different methods. (C) Top 20 differentially expressed gene enriched terms identified by DisGeNET (left panel) and TRRUST (right panel).

previously reported to be upregulated in myeloid progenitor populations, is also confirmed in our study [67]. The genes COL3A1 and COL1A1 shows significant positive correlations with neutrophils [68, 69]. Additionally, the oncogene SOX4 is prominently featured in our differential analysis of tumor cells [70]. These genes are also identified as diagnostic or prognostic biomarkers in previous studies [68, 71, 72, 73, 74]. Notably, some cell types also share similar gene expression patterns (Fig. 6C). For example, epithelial cells and tumor cells exhibit expression similarities. Multiple studies using single-cell transcriptomics analysis have revealed that lung cancer cells share characteristics similar to those of Type 1 and Type 2 alveolar epithelial cells [75, 76]. This similarity may be related to lung cancer cells maintaining epithelial cell functions, such as cell adhesion and migration [77, 78].

We also conducted a visualization analysis comparing the results of stMMR applied to 20 tissue sections with the actual division

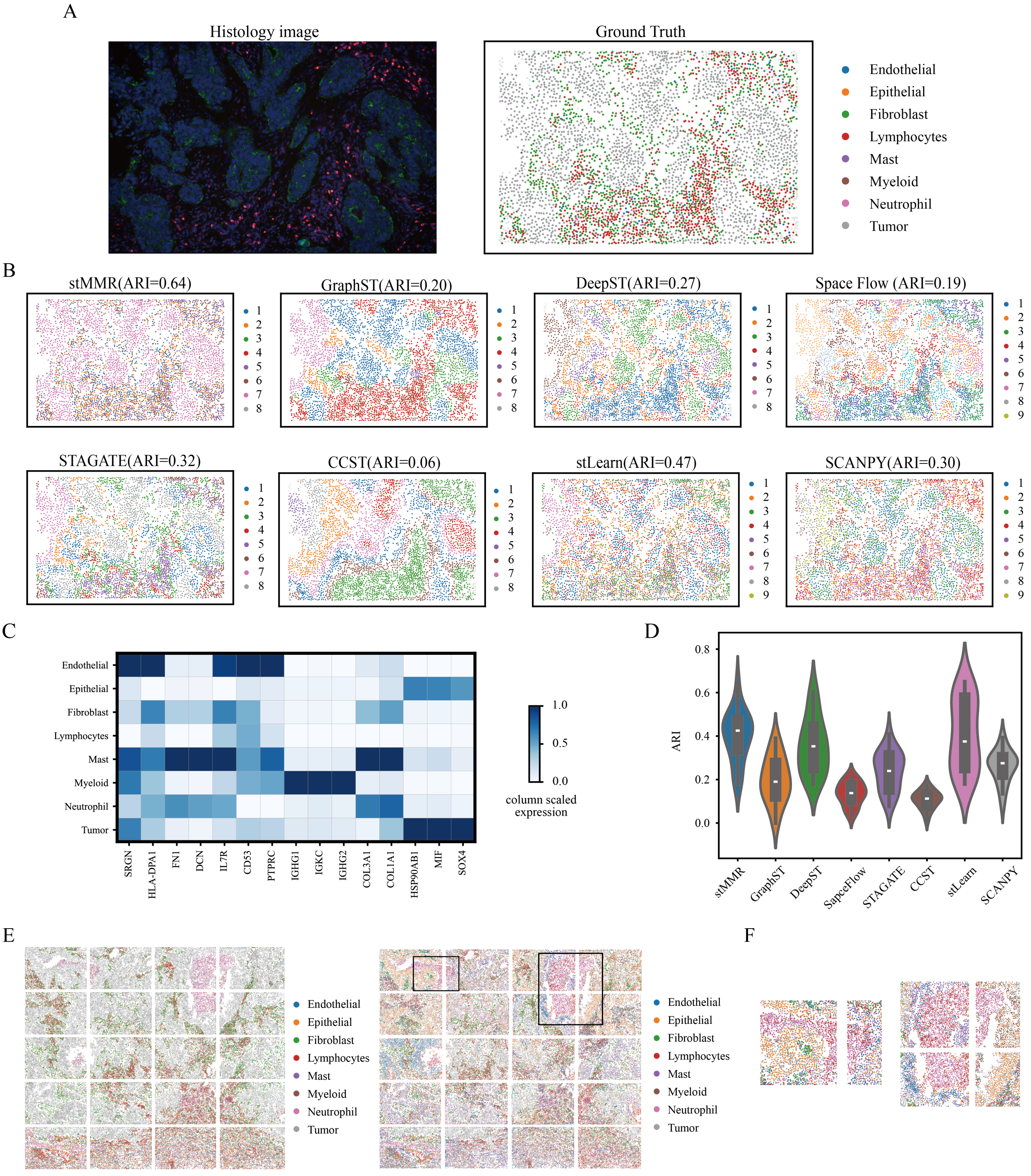

Figure 6. stMMR recognizes cell type differences in lung cancer dataset. (A) One FOV of the lung cancer SRT data. (B) Cell types identified by different methods. (C) Expression pattern of marker genes for different cell types. (D) The overall performance of different methods across 20 FOVs. (E) Cell types annotated manually in 20 FOVs. (F) Cell types annotated by stMMR in 20 FOVs. (G) The zoomed-in results of boundaries between adjacent FOVs identified by stMMR.

of tissue regions. The analysis demonstrates that stMMR effectively identifies tissue regions across multiple sections (Fig. 6E). Notably, even in regions bisected by section boundaries, stMMR maintains smooth and continuous (Fig. 6E and F). These findings indicate that the joint representation of stMMR not only effectively eliminates noise from different data types but also maintains excellent performance in the recognition of tissue regions across multiple slices.

Discussion

SRT technology enables us to deeply understand the spatial structure of tissues within biological systems from multiple dimensions, including gene expression profiles, spatial location, and histological information. However, the inherent data heterogeneity along with the varying spatial resolutions presents challenges in the integration of these modalities. To harmonize and unify multi-modal data as well as achieve effective joint representation for multi-modal SRT data, we propose a novel computational framework, stMMR.

stMMR effectively unifies gene expression profiles and histological information by utilizing spatial location as a connecting link. This method automates the construction of adjacency relationships between neighboring spots. Then, GCN is employed to extract features from both gene expression profiles and histological information. Furthermore, stMMR adopts an innovative strategy for representing intra-modal and inter-modal features. Initially, it employs an attention mechanism for an in-depth learning within a single modality. It then integrates cross-modality features through a combination of similarity contrastive learning, along with the reconstruction of gene expression and adjacency relationship. By applying stMMR to SRT data of various tissues and resolutions, we have validated its exceptional performance in multiple analyses, including domain identification, pseudo-spatiotemporal analysis, gene expression data enhancement as well as the identification of domain-specific genes.

The remarkable performance of stMMR can be attributed to several innovative designs, as confirmed by ablation studies (Supplementary Fig. S2). The crucial aspect is the integration of histological information with gene expression data through spatial location. In SRT, gene expression data suffers from issues of sparsity and zero inflation, which are key factors that interfere with downstream analysis [33, 79]. Previous research has shown that histological information can predict gene expression data [30, 31, 32]. Therefore, compared to methods that rely only on gene expression information, stMMR integrates imaging information and exhibits superior performance in spot characterization. Secondly, unlike other methods that construct spatial transcriptomic data as unweighted graphs, stMMR builds undirected weighted graphs inversely proportional to Euclidean distances between spots, better reflecting the influence of spatial distance on message passing and aggregation. Furthermore, the consideration of relationships within and between modalities is also crucial. Sole reliance on gene expression data for correlation analysis may result in information loss. In contrast, methods that incorporate imaging information, such as DeepST, focus primarily on the integration of multi-modal data, overlooking the relationships within individual modalities. To fully leverage the relationships within and between modalities, stMMR not only uses similarity contrastive learning for integrating features across modalities but also incorporates a self-attention module for deep embedding of features within a modality. Additionally, the reconstruction modules for gene expression and adjacency matrix further encourage the model to retain as much original information as possible. This encoder-decoder structure improves the ability of stMMR to recover information also endows stMMR with denoising capabilities and robustness.

Notably, stMMR possesses strong scalability. It can be easily applied to other data derived from diverse experimental techniques. Beyond the previously mentioned datasets, we applied stMMR to the analysis of a mouse brain dataset derived using 10x Visium technology, a human pancreatic ductal adenocarcinoma dataset obtained through ST technology, and a human colorectal cancer (CRC) dataset generated using the newly introduced 10x Visium HD technology (Supplementary Fig. S3, S4 and S5). In these tests, the stMMR consistently achieved optimal results. Furthermore, we extended stMMR to integrate and analyze multi-slice data (Supplementary Fig. S6). Additionally, by duplicating the gene expression module, the framework of stMMR can be directly used for integration of features from proteomes or epigenomics [80, 81, 82, 83].

There is still room for the improvement of stMMR. Currently, stMMR employs Euclidean distance in the construction of spot adjacency matrices. However, in practical scenarios, it may be more rational to utilize different distance metrics for graph construction based on modal features. For instance, considering gene expression data, the use of Pearson Correlation Coefficients or K-L divergence might be more appropriate to measure expression similarity between spots. In contrast, for spatial imaging data, either Euclidean distance or staining similarity can serve as the distance metric. Under these circumstances, the constructed graph transitions from being a homogenous graph to a heterogeneous one. For such heterogeneous graphs with multiple types of edges, we can apply methods like metapath2vec or multi-view learning to achieve embedding and integration of different modalities [84, 85, 86, 87].

In this paper, we introduce a robust and accurate tool, stMMR, for the integration of gene expression data, spatial information, and histological information from SRT data. Compared to existing methods, stMMR demonstrates a significant advantage in integrating multi-modal data, particularly excelling in domain identification, pseudo-spatiotemporal analysis, and domain-specific gene analysis. Overall, as an effective and user-friendly tool, stMMR enhances the multi-modal joint analysis of SRT data, providing substantial support for research in relevant fields.

Availability of Source Code and Requirements

Project name: stMMR
Project home page: `https://github.com/nayu0419/stMMR`
Operating system(s): Linux
Programming language: Python
Other requirements: Python 3.9.1 or higher
License: MIT license
RRID: SCR_025601
BiotoolsID: stmmr

Additional Files

Supplementary Fig. S1. The results of the regions identified by stMMR in 12 DLPFC slices.

Supplementary Fig. S2. Ablation study results for stMMR on a breast cancer dataset. (A) The spatial clustering result of stMMR. (B) stMMR without histological information, resulting in a lower ARI of 0.57, indicating the importance of histology in improving spatial domain identification. (C) stMMR without GCN, which yields a significantly reduced ARI of 0.49, showing the critical role of GCN in capturing local spatial relationships. (D) stMMR without the attention mechanism, leading to a decrease in ARI to 0.60, demonstrating the importance of global attention in enhancing overall model performance.

Supplementary Fig. S3. The domain recognition results of 10x mouse brain dataset. (A) The histology image and (B) manually annotated tissue structures. (C) The domain recognition results by different methods.

Supplementary Fig. S4. The domain recognition results of human pancreatic ductal adenocarcinoma. (A) The histology image and (B) annotated tissue structures. (C) The domain recognition results by different methods.

Supplementary Fig. S5. Spatial clustering results on the human colorectal cancer. (A) The histology image. (B) The domain recognition results by different methods.

Supplementary Fig. S6. Spatial domain identification results from stMMR integrating four slices, and the ground truth for the four slices.

Supplementary Table S1. Summary of the datasets used in this study.

Data availability

All datasets used in this paper are publicly available and listed in Supplementary Section 2. Processed datasets are also available at SODB (`https://gene.ai.tencent.com/SpatialOmics/`) and can be loaded by PySODB (`https://protocols-pysodb.readthedocs.io/en/latest/`). The domain-specific genes found by stMMR are available on the stMMR GitHub page.

Abbreviations

SRT: spatially resolved transcriptomics; GCN: graph convolutional networks; H&E: hematoxylin and eosin; ViT: Vision Transformer; ZINB: zero-inflated negative binomial; DLPFC: dorsolateral prefrontal cortex; pSM: pseudo-spatiotemporal map; ARI: Adjusted Rand Index; CRC: colorectal cancer.

Competing Interests

The authors declare they have no competing interests.

Funding

This work was supported by the National Natural Science Foundation of China (Nos. 62303271, U1806202, 62373216, 92374107)and Natural Science Foundation of Shandong Province (ZR2023QF081).

Authors' Contributions

R.G., W.Z. and Z.Y. conceived and supervised the project. D.Z. and N.Y. designed the model and developed the stMMR software. D.Z., N.Y. and W.Z. wrote the manuscript. X.S. and Q.Z. collected and constructed the benchmark datasets. W.L. X.L. and Z.L. conducted biological interpretation. All authors approved the manuscript.

Acknowledgements

Not applicable.

References

1. Chen KH, Boettiger AN, Moffitt JR, et al. Spatially resolved, highly multiplexed RNA profiling in single cells. Science 2015;348(6233):aaa6090. https://doi.org/10.1126/science.aaa6090.
2. Asp M, Bergenstråhle J, Lundeberg J. Spatially Resolved Transcriptomes—Next Generation Tools for Tissue Exploration. BioEssays 2020;42(10):1900221. https://doi.org/10.1002/bies.201900221.
3. Eng CHL, Lawson M, Zhu Q, et al. Transcriptome-scale super-resolved imaging in tissues by RNA seqFISH+. Nature 2019;568(7751):235–239. https://doi.org/10.1038/s41586-019-1049-y.
4. Wang X, Allen WE, Wright MA, et al. Three-dimensional intact-tissue sequencing of single-cell transcriptional states. Science 2018;361(6400):eaat5691. https://doi.org/10.1126/science.aat5691.
5. Ståhl PL, Salmén F, Vickovic S, et al. Visualization and analysis of gene expression in tissue sections by spatial transcriptomics. Science 2016;353(6294):78–82. https://doi.org/10.1126/science.aaf2403.
6. Rodriques SG, Stickels RR, Goeva A, et al. Slide-seq: A scalable technology for measuring genome-wide expression at high spatial resolution. Science 2019;363(6434):1463–1467. https://doi.org/10.1126/science.aaw1219.
7. Marx V. Method of the Year: spatially resolved transcriptomics. Nat Methods 2021;18(1):9–14. https://doi.org/10.1038/s41592-020-01033-y.
8. Guo T, Yuan Z, Pan Y, et al. SPIRAL: integrating and aligning spatially resolved transcriptomics data across different experiments, conditions, and technologies. Genome Biol 2023;24(1):241. https://doi.org/10.1186/s13059-023-03078-6.
9. Huo Y, Guo Y, Wang J, et al. Integrating multi-modal information to detect spatial domains of spatial transcriptomics by graph attention network. J Genet Genomics 2023;50(9):720–733. https://doi.org/10.1016/j.jgg.2023.06.005.
10. Son CG, Bilke S, Davis S, et al. Database of mRNA gene expression profiles of multiple human organs. Genome Res 2005;15(3):443–450. https://doi.org/10.1101/gr.3124505.
11. Hannig J, Schäfer H, Ackermann J, et al. Bioinformatics analysis of whole slide images reveals significant neighborhood preferences of tumor cells in Hodgkin lymphoma. PLoS Comput Biol 2020;16(1):e1007516. https://doi.org/10.1371/journal.pcbi.1007516.
12. Haghighi M, Caicedo JC, Cimini BA, et al. High-dimensional gene expression and morphology profiles of cells across 28,000 genetic and chemical perturbations. Nat Methods 2022;19(12):1550–1557. https://doi.org/10.1038/s41592-022-01667-0.
13. Li Z, Chen X, Zhang X, et al. Latent feature extraction with a prior-based self-attention framework for spatial transcriptomics. Genome Res 2023;33(10):1757–1773. https://doi.org/10.1101/gr.277891.123.
14. Li Z, Zhou X. BASS: multi-scale and multi-sample analysis enables accurate cell type clustering and spatial domain detection in spatial transcriptomic studies. Genome Biol 2022;23(1):168. https://doi.org/10.1186/s13059-022-02734-7.
15. Zhao E, Stone MR, Ren X, et al. Spatial transcriptomics at subspot resolution with BayesSpace. Nat Biotechnol 2021;39(11):1375–1384. https://doi.org/10.1038/s41587-021-00935-2.
16. Dries R, Zhu Q, Dong R, et al. Giotto: a toolbox for integrative analysis and visualization of spatial expression data. Genome Biol 2021;22:78. https://doi.org/10.1186/s13059-021-02286-2.
17. Varrone M, Tavernari D, Santamaria-Martínez A, et al. CellCharter reveals spatial cell niches associated with tissue remodeling and cell plasticity. Nat Genet 2024;56(1):74–84. https://doi.org/10.1038/s41588-023-01588-4.
18. Liu W, Liao X, Luo Z, et al. Probabilistic embedding, clustering, and alignment for integrating spatial transcriptomics data with PRECAST. Nat Commun 2023;14(1):296. https://doi.org/10.1038/s41467-023-35947-w.
19. Yuan Z. MENDER: fast and scalable tissue structure identification in spatial omics data. Nat Commun 2024;15(1):207. https://doi.org/10.1038/s41467-023-44367-9.
20. Li J, Chen S, Pan X, et al. Cell clustering for spatial transcriptomics data with graph neural networks. Nat Comput Sci 2022;2(6):399–408. https://doi.org/10.1038/s43588-022-00266-5.
21. Dong K, Zhang S. Deciphering spatial domains from spatially resolved transcriptomics with an adaptive graph attention auto-encoder. Nat Commun 2022;13(1):1739. https://doi.org/10.1038/s41467-022-29439-6.
22. Ren H, Walker BL, Cang Z, et al. Identifying multicellular spatiotemporal organization of cells with SpaceFlow. Nat Commun 2022;13(1):4076. https://doi.org/10.1038/s41467-022-31739-w.
23. Long Y, Ang KS, Li M, et al. Spatially informed clustering, integration, and deconvolution of spatial transcriptomics with GraphST. Nat Commun 2023;14(1):1155. https://doi.org/10.1038/s41467-023-36796-3.
24. Pham D, Tan X, Balderson B, et al. Robust mapping of spatiotemporal trajectories and cell–cell interactions in healthy and diseased tissues. Nat Commun 2023;14(1):7739. https://doi.org/10.1038/s41467-023-43120-6.
25. Xu C, Jin X, Wei S, et al. DeepST: identifying spatial domains in spatial transcriptomics by deep learning. Nucleic Acids Res 2022;50(22):e131–e131. https://doi.org/10.1093/nar/gkac901.

26. He S, Bhatt R, Brown C, et al. High-plex imaging of RNA and proteins at subcellular resolution in fixed tissue by spatial molecular imaging. Nat Biotechnol 2022;40(12):1794–1806. https://doi.org/10.1038/s41587-022-01483-z.
27. Dosovitskiy A, Beyer L, Kolesnikov A, et al. An Image is Worth 16x16 Words: Transformers for Image Recognition at Scale. arXiv preprint arXiv:201011929 2020;https://doi.org/10.48550/arXiv.2010.11929.
28. Kipf TN, Welling M. Semi-Supervised Classification with Graph Convolutional Networks. arXiv preprint arXiv:160902907 2016;https://doi.org/10.48550/arXiv.1609.02907.
29. Brauwers G, Frasincar F. A General Survey on Attention Mechanisms in Deep Learning. IEEE Trans Knowl Data Eng 2023;35(4):3279–3298. https://doi.org/10.1109/TKDE.2021.3126456.
30. Markey M, Kim J, Goldstein Z, et al. Spatially-resolved prediction of gene expression signatures in H&E whole slide images using additive multiple instance learning models. Mol Cancer Ther 2023;22(12_Supplement):B010–B010. https://doi.org/10.1158/1535-7163.TARG-23-B010.
31. Bergenstråhle L, He B, Bergenstråhle J, et al. Super-resolved spatial transcriptomics by deep data fusion. Nat Biotechnol 2022;40(4):476–479. https://doi.org/10.1038/s41587-021-01075-3.
32. Zeng Y, Wei Z, Yu W, et al. Spatial transcriptomics prediction from histology jointly through Transformer and graph neural networks. Brief Bioinform 2022;23(5):bbac297. https://doi.org/10.1093/bib/bbac297.
33. Covert I, Gala R, Wang T, et al. Predictive and robust gene selection for spatial transcriptomics. Nat Commun 2023;14(1):2091. https://doi.org/10.1038/s41467-023-37392-1.
34. Yu Z, Lu Y, Wang Y, et al. ZINB-Based Graph Embedding Autoencoder for Single-Cell RNA-Seq Interpretations. In: AAAI Conf Artif Intell, vol. 36; 2022. p. 4671–4679. https://doi.org/10.1609/aaai.v36i4.20392.
35. Kipf TN, Welling M. Variational Graph Auto-Encoders. arXiv preprint arXiv:161107308 2016;https://doi.org/10.48550/arXiv.1611.07308.
36. Tang M, Yang C, Li P. Graph Auto-Encoder Via Neighborhood Wasserstein Reconstruction. arXiv preprint arXiv:220209025 2022;https://doi.org/10.48550/arXiv.2202.09025.
37. Wolf FA, Angerer P, Theis FJ. SCANPY: large-scale single-cell gene expression data analysis. Genome Biol 2018;19:15. https://doi.org/10.1186/s13059-017-1382-0.
38. Li B, Zhang W, Guo C, et al. Benchmarking spatial and single-cell transcriptomics integration methods for transcript distribution prediction and cell type deconvolution. Nat Methods 2022;19(6):662–670. https://doi.org/10.1038/s41592-022-01480-9.
39. Cheng A, Hu G, Li WV. Benchmarking cell-type clustering methods for spatially resolved transcriptomics data. Brief Bioinform 2023;24(1):bbac475. https://doi.org/10.1093/bib/bbac475.
40. Zhu J, Shang L, Zhou X. SRTsim: spatial pattern preserving simulations for spatially resolved transcriptomics. Genome Biol 2023;24(1):39. https://doi.org/10.1186/s13059-023-02879-z.
41. Shang L, Zhou X. Spatially aware dimension reduction for spatial transcriptomics. Nat Commun 2022;13(1):7203. https://doi.org/10.1038/s41467-022-34879-1.
42. Maynard KR, Collado-Torres L, Weber LM, et al. Transcriptome-scale spatial gene expression in the human dorsolateral prefrontal cortex. Nat Neurosci 2021;24(3):425–436. https://doi.org/10.1038/s41593-020-00787-0.
43. Wolf FA, Hamey FK, Plass M, et al. PAGA: graph abstraction reconciles clustering with trajectory inference through a topology preserving map of single cells. Genome Biol 2019;20:59. https://doi.org/10.1186/s13059-019-1663-x.
44. Gao J, Zhang F, Hu K, et al. Hexagonal Convolutional Neural Network for Spatial Transcriptomics Classification. In: IEEE BIBM; 2022. p. 200–205. https://doi.org/10.1002/bies.201900221.
45. Avşar G, Pir P. A comparative performance evaluation of imputation methods in spatially resolved transcriptomics data. Mol Omics 2023;19(2):162–173. https://doi.org/10.1039/d2mo00266c.
46. Lopez R, Li B, Keren-Shaul H, et al. DestVI identifies continuums of cell types in spatial transcriptomics data. Nat Biotechnol 2022;40(9):1360–1369. https://doi.org/10.1038/s41587-022-01272-8.
47. Wang Y, Song B, Wang S, et al. Sprod for de-noising spatially resolved transcriptomics data based on position and image information. Nat Methods 2022;19(8):950–958. https://doi.org/10.1038/s41592-022-01560-w.
48. Arion D, Enwright JF, Gonzalez-Burgos G, et al. Differential gene expression between callosal and ipsilateral projection neurons in the monkey dorsolateral prefrontal and posterior parietal cortices. Cereb Cortex 2022;33(5):1581–1594. https://doi.org/10.1093/cercor/bhac157.
49. Arnsten AFT, Woo E, Yang S, et al. Unusual molecular regulation of dorsolateral prefrontal cortex layer III synapses increases vulnerability to genetic and environmental insults in schizophrenia. Biol Psychiatry 2022;92(6):480–490. https://doi.org/10.1016/j.biopsych.2022.02.003.
50. Mantri M, Scuderi GJ, Abedini-Nassab R, et al. Spatiotemporal single-cell RNA sequencing of developing chicken hearts identifies interplay between cellular differentiation and morphogenesis. Nat Commun 2021;12(1):1771. https://doi.org/10.1038/s41467-021-21892-z.
51. Haghverdi L, Büttner M, Wolf FA, et al. Diffusion pseudotime robustly reconstructs lineage branching. Nat Methods 2016;13(10):845–848. https://doi.org/10.1038/nmeth.3971.
52. Martinsen BJ. Reference guide to the stages of chick heart embryology. Dev Dyn 2005;233(4):1217–1237. https://doi.org/10.1002/dvdy.20468.
53. Lu ZQ, Sinha A, Sharma P, et al. Proteomic Analysis of Human Fetal Atria and Ventricle. J Proteome Res 2014;13(12):5869–5878. https://doi.org/10.1021/pr5007685.
54. Siegel RL, Miller KD, Fuchs HE, et al. Cancer statistics, 2022. CA Cancer J Clin 2022;72(1):7–33. https://doi.org/10.3322/caac.21708.
55. Piñero J, Queralt-Rosinach N, Bravo A, et al. DisGeNET: a discovery platform for the dynamical exploration of human diseases and their genes. Database (Oxford) 2015;2015:bav028. https://doi.org/10.1093/database/bav028.
56. Kang D, Yoon SE, Shin D, et al. Risk of non-Hodgkin lymphoma in breast cancer survivors: a nationwide cohort study. Blood Cancer J 2021;11(12):1–8. https://doi.org/10.1038/s41408-021-00595-0.
57. Berger E, Delpierre C, Hosnijeh FS, et al. Association between low-grade inflammation and Breast cancer and B-cell Myeloma and Non-Hodgkin Lymphoma: findings from two prospective cohorts. Sci Rep 2018;8(1):10805. https://doi.org/10.1038/s41598-018-29041-1.
58. Zhao H, Wu L, Yan G, et al. Inflammation and tumor progression: signaling pathways and targeted intervention. Signal Transduct Target Ther 2021;6(1):1–46. https://doi.org/10.1038/s41392-021-00658-5.
59. McAndrew NP, Bottalico L, Mesaros C, et al. Effects of systemic inflammation on relapse in early breast cancer. NPJ Breast Cancer 2021;7(1):1–10. https://doi.org/10.1038/s41523-020-00212-6.
60. Han H, Shim H, Shin D, et al. TRRUST: a reference database of human transcriptional regulatory interactions. Sci Rep 2015;5(1):11432. https://doi.org/10.1038/srep11432.
61. Gao Y, Gan K, Liu K, et al. SP1 Expression and the Clinicopathological Features of Tumors: A Meta-Analysis and Bioin-

formatics Analysis. Pathol Oncol Res 2021;27:581998. https://doi.org/10.3389/pore.2021.581998.
62. Wang W, Nag SA, Zhang R. Targeting the NFκB signaling pathways for breast cancer prevention and therapy. Curr Med Chem 2015;22(2):264–289. https://doi.org/10.2174/0929867321666141106124315.
63. Kanzaki H, Chatterjee A, Hossein Nejad Ariani H, et al. Disabling the nuclear translocalization of RelA/NF-κB by a small molecule inhibits triple-negative breast cancer growth. Breast Cancer (Dove Med Press) 2021;13:419–430. https://doi.org/10.2147/BCTT.S310231.
64. Kim GC, Kwon HK, Lee CG, et al. Upregulation of Ets1 expression by NFATc2 and NFKB1/RELA promotes breast cancer cell invasiveness. Oncogenesis 2018;7(11):1–15. https://doi.org/10.1038/s41389-018-0101-3.
65. Jeong YJ, Oh HK, Choi HR. Methylation of the RELA gene is associated with expression of NF-κB1 in response to TNF-α in breast cancer. Molecules 2019;24(15):2834. https://doi.org/10.3390/molecules24152834.
66. Wellenstein MD, Coffelt SB, Duits DEM, et al. Loss of p53 triggers WNT-dependent systemic inflammation to drive breast cancer metastasis. Nature 2019;572(7770):538–542. https://doi.org/10.1038/s41586-019-1450-6.
67. Mincarelli L, Uzun V, Wright D, et al. Single-cell gene and isoform expression analysis reveals signatures of ageing in haematopoietic stem and progenitor cells. Commun Biol 2023;6(1):1–11. https://doi.org/10.1038/s42003-023-04936-6.
68. Zhang H, Ding C, Li Y, et al. Data mining-based study of collagen type III alpha 1 (COL3A1) prognostic value and immune exploration in pan-cancer. Bioengineered 2021;12(1):3634–3646. https://doi.org/10.1080/21655979.2021.1949838.
69. Ren J, Da J, Hu N. Identification of COL1A1 associated with immune infiltration in brain lower grade glioma. PLoS One 2022;17(7):e0269533. https://doi.org/10.1371/journal.pone.0269533.
70. Moreno CS. SOX4: The Unappreciated Oncogene. Semin Cancer Biol 2020;67:57–64. https://doi.org/10.1016/j.semcancer.2019.08.027.
71. Tang M, Liu P, Wu X, et al. COL3A1 and Its Related Molecules as Potential Biomarkers in the Development of Human Ewing's Sarcoma. Biomed Res Int 2021;2021(1):7453500. https://doi.org/10.1155/2021/7453500.
72. Geng Q, Shen Z, Li L, et al. COL1A1 is a prognostic biomarker and correlated with immune infiltrates in lung cancer. Peerj 2021;9:e11145. https://doi.org/10.7717/peerj.11145.
73. Walter RFH, Mairinger FD, Werner R, et al. SOX4, SOX11 and PAX6 mRNA expression was identified as a (prognostic) marker for the aggressiveness of neuroendocrine tumors of the lung by using next-generation expression analysis (NanoString). Future Oncol 2015;11(7):1027–1036. https://doi.org/10.2217/fon.15.18.
74. Srivastava M, Khurana P, Sugadev R. Lung Cancer Signature Biomarkers: tissue specific semantic similarity based clustering of Digital Differential Display (DDD) data. Bmc Res Notes 2012;5:617. https://doi.org/10.1186/1756-0500-5-617.
75. Wang Z, Li Z, Zhou K, et al. Deciphering cell lineage specification of human lung adenocarcinoma with single-cell RNA sequencing. Nat Commun 2021;12(1):6500. https://doi.org/10.1038/s41467-021-26770-2.
76. Zuo W, Rostami MR, Shenoy SA, et al. Cell-specific expression of lung disease risk-related genes in the human small airway epithelium. Respir Res 2020;21(1):200. https://doi.org/10.1186/s12931-020-01442-9.
77. Janiszewska M, Primi MC, Izard T. Cell adhesion in cancer: Beyond the migration of single cells. J Biol Chem 2020;295(8):2495–2505. https://doi.org/10.1074/jbc.REV119.007759.
78. Millar FR, Janes SM, Giangreco A. Epithelial cell migration as a potential therapeutic target in early lung cancer. Eur Respir Rev 2017;26(143). https://doi.org/10.1183/16000617.0069-2016.
79. Liu Z, Wu D, Zhai W, et al. SONAR enables cell type deconvolution with spatially weighted Poisson-Gamma model for spatial transcriptomics. Nat Commun 2023;14(1):4727. https://doi.org/10.1038/s41467-023-40458-9.
80. Deng Y, Bartosovic M, Kukanja P, et al. Spatial-CUT&Tag: Spatially resolved chromatin modification profiling at the cellular level. Science 2022;375(6581):681–686. https://doi.org/10.1126/science.abg7216.
81. Zhang D, Deng Y, Kukanja P, et al. Spatial epigenome–transcriptome co-profiling of mammalian tissues. Nature 2023;616(7955):113–122. https://doi.org/10.1038/s41586-023-05795-1.
82. Xie Y, Ruan F, Li Y, et al. Spatial chromatin accessibility sequencing resolves high-order spatial interactions of epigenomic markers. Elife 2024;12:RP87868. https://doi.org/10.7554/eLife.87868.4.
83. Deng Y, Bartosovic M, Ma S, et al. Spatial profiling of chromatin accessibility in mouse and human tissues. Nature 2022;609(7926):375–383. https://doi.org/10.1038/s41586-022-05094-1.
84. Dong Y, Chawla NV, Swami A. metapath2vec: Scalable Representation Learning for Heterogeneous Networks. In: ACM SIGKDD International Conference on Knowledge Discovery & Data Mining; 2017. p. 135–144. https://doi.org/10.1145/3097983.3098036.
85. Li X, Chen W, Chen Y, et al. Network embedding-based representation learning for single cell RNA-seq data. Nucleic Acids Res 2017;45(19):e166–e166. https://doi.org/10.1093/nar/gkx750.
86. Guo T, Chen Y, Shi M, et al. Integration of single cell data by disentangled representation learning. Nucleic Acids Res 2022;50(2):e8–e8. https://doi.org/10.1093/nar/gkab978.
87. Wu G, Li X, Guo W, et al. JEBIN: analyzing gene co-expressions across multiple datasets by joint network embedding. Brief Bioinform 2022;23(2):bbab603. https://doi.org/10.1093/bib/bbab603.

Response to the reviewers

Click here to access/download
Supplementary Material
Response Letter.pdf

Click here to access/download
Supplementary Material
Supplementary Materials.docx

Click here to access/download
Supplementary Material
Supplementary_Figure_S1.jpg

Click here to access/download
Supplementary Material
Supplementary_Figure_S2.jpg

Click here to access/download
Supplementary Material
Supplementary_Figure_S3.jpg

Click here to access/download
Supplementary Material
Supplementary_Figure_S4.jpg

Click here to access/download
Supplementary Material
Supplementary_Figure_S5.jpg

Click here to access/download
Supplementary Material
Supplementary_Figure_S6.jpg

Click here to access/download
Supplementary Material
Supplementary_Table_S1.docx

Cover Letter Click here to access/download;Personal Cover;Cover Letter.pdf

Manuscript ID: GIGA-D-24-00153
Title: stMMR: accurate and robust spatial domain identification from spatially resolved transcriptomics with multi-modal feature representation

Dear Editor:

Thank you and the reviewers for the great efforts to deal with our manuscript as well as careful and detailed comments. The comments pointed out some deficiencies of our work, and thereby helped us to substantially improve the quality of the manuscript. We have modified our manuscript, supplementary as well as the github document. Below is the summary of our revision:

- We have revised our manuscript to include comprehensive technical explanations that clarify the necessity and novelty of our method. Additionally, we added the analyses of colorectal cancer data from the latest 10x Visium HD technology, as well as multi-slice SRT data, which further demonstrate the scalability of stMMR.
- We performed ablation studies to measure the critical role of each stMMR component, including GCN, global attention mechanism, and histological information.
- We updated the Github repository with a detailed tutorial. We also shared all analysis code used in this study to ensure reproducibility.
- We have registered our software in the bio.tools and SciCrunch.org databases. The identifiers RRID: SCR_025601 and BiotoolsID: stmmr have been included in the revised manuscript. Furthermore, we have ensured that the revised manuscript adheres to the journal's style guidelines.
- In accordance with the journal's policy, we have designated Rui Gao and Wei Zhang as the corresponding authors. Daoliang Zhang, Na Yu (the primary reviser during the revision process), and Zhiyuan Yuan (one of the original corresponding authors) are listed as co-first authors. This change ensures that the key contributions of all individuals are properly acknowledged.

The revised manuscript has been approved by all the authors. Thank you again for your consideration.

Best regards,

Wei Zhang *et al*.
School of Control Science and Engineering
Shandong University
Jinan 250061, P.R. China
