# Supplementary material for: stMMR: accurate and robust spatial domain identification from spatially resolved transcriptomics with multimodal feature representation
Source: Gigascience. 2024 Nov 28;13:giae089. doi: 10.1093/gigascience/giae089 (PMC11604062; doi:10.1093/gigascience/giae089)
Supplement: giae089_Response_to_Reviewer_Comments_Original_Submission [file giae089_response_to_reviewer_comments_original_submission.pdf]

Author's Response To Reviewer Comments

Manuscript ID: GIGA-D-24-00153
Title: stMMR: accurate and robust spatial domain identification from spatially resolved transcriptomics with multi-modal feature representation

Dear Reviewers,

We sincerely thank you for the great efforts to deal with our manuscript as well as the constructive and thoughtful feedback. These comments encouraged us to substantially improve the quality of our work. According to your comments and suggestions, we have revised our manuscript and source code thoroughly.

Please find attached a revised version of our manuscript and detailed responses to the reviewers' comments (as a separate PDF file titled “Response Letter.pdf” as Supplemental Material).

Thank you once again for the valuable feedback on our work.

Wei Zhang et al.
School of Control Science and Engineering
Shandong University
Jinan 250061, P.R. China
