# Supplementary material for: stMMR: accurate and robust spatial domain identification from spatially resolved transcriptomics with multimodal feature representation
Source: Gigascience. 2024 Nov 28;13:giae089. doi: 10.1093/gigascience/giae089 (PMC11604062; doi:10.1093/gigascience/giae089)
Supplement: giae089_Reviewer_1_Report_Original_Submission — Shihua Zhang -- 7/3/2024 [file giae089_reviewer_1_report_original_submission.pdf]

Reviewer Report

Title: stMMR: accurate and robust spatial domain identification from spatially resolved transcriptomics with multi-modal feature representation

Version: Original Submission **Date:** 7/3/2024

Reviewer name: Shihua Zhang

Reviewer Comments to Author:

In this paper, the authors developed a multi-modal deep learning method for identifying spatial domains from ST data by integrating gene expression, spatial location and histological information. This method adopts the graphconvolutional networks and self-attention module for deep embedding of features within unimodal and incorporates similarity contrastive learning for integrating features across modalities. They did several typical analysis to valid this this method. Generally, the wiring of this paper is OK. More specific comments:1. Spatial domain has been overwhelmingly studied recently. The authors need to pay more attention to why it is needed to introduce a new method. The novelty of the current method should be carefully clarified. For example, how the histological information help to improve the performance? Does the "geometric" deep learning really help?2. This method has been applied to some stereotypical data. The authors should applied it to some recently generated data by some new ST techniques.3. Figure 3 stMMR enhances spatial gene expression profiles. It is hard to see how the method enhance the spatial gene expression (e.g., LPL).4. With the accumulation of multi-slice spatial transcriptome data, the integration and alignment of spatial transcriptome data will be essential. Can this method be extended for this situation like STAGATE (Nat Comput Sci.2023 Oct; 3(10):894-906)? This will be valuable for ST analysis.5. The scalability of this method should be carefully explored.6. The authors should provide a detailed tutorial for users.

Level of Interest

Please indicate how interesting you found the manuscript: Choose an item.

Quality of Written English

Please indicate the quality of language in the manuscript: Choose an item.

Declaration of Competing Interests

Please complete a declaration of competing interests, considering the following questions:

- Have you in the past five years received reimbursements, fees, funding, or salary from an organisation that may in any way gain or lose financially from the publication of this manuscript, either now or in the future?

- Do you hold any stocks or shares in an organisation that may in any way gain or lose financially from the publication of this manuscript, either now or in the future?
- Do you hold or are you currently applying for any patents relating to the content of the manuscript?
- Have you received reimbursements, fees, funding, or salary from an organization that holds or has applied for patents relating to the content of the manuscript?
- Do you have any other financial competing interests?
- Do you have any non-financial competing interests in relation to this paper?

If you can answer no to all of the above, write 'I declare that I have no competing interests' below. If your reply is yes to any, please give details below.

I declare that I have no competing interests

I agree to the open peer review policy of the journal. I understand that my name will be included on my report to the authors and, if the manuscript is accepted for publication, my named report including any attachments I upload will be posted on the website along with the authors' responses. I agree for my report to be made available under an Open Access Creative Commons CC-BY license (http://creativecommons.org/licenses/by/4.0/). I understand that any comments which I do not wish to be included in my named report can be included as confidential comments to the editors, which will not be published.

Choose an item.

To further support our reviewers, we have joined with Publons, where you can gain additional credit to further highlight your hard work (see: https://publons.com/journal/530/gigascience). On publication of this paper, your review will be automatically added to Publons, you can then choose whether or not to claim your Publons credit. I understand this statement.

Yes Choose an item.
