# Supplementary material for: stMMR: accurate and robust spatial domain identification from spatially resolved transcriptomics with multimodal feature representation
Source: Gigascience. 2024 Nov 28;13:giae089. doi: 10.1093/gigascience/giae089 (PMC11604062; doi:10.1093/gigascience/giae089)
Supplement: giae089_Reviewer_2_Report_Original_Submission — Hongzhi Wen -- 7/18/2024 [file giae089_reviewer_2_report_original_submission.pdf]

Reviewer Report

Title: stMMR: accurate and robust spatial domain identification from spatially resolved transcriptomics with multi-modal feature representation

Version: Original Submission **Date:** 7/18/2024

Reviewer name: Hongzhi Wen

Reviewer Comments to Author:

The paper introduces stMMR, a multi-modal graph learning method designed to integrate gene expression, spatial location, and histological information for accurate spatial domain identification from spatially resolved transcriptomics (SRT) data. The method employs graph convolutional networks (GCN) and self-attention modules, along with cross-modal contrastive learning, to enhance feature integration and representation.Strengths:1. Using GCN to capture local spatial dependency is natural and effective. Introducing attention mechanism for capturing global relations intuitively make senses, however, need more justification. Contrastive learning for cross-modal feature fusion is also a natural choice in multi-modal learning. Overall, the methodology is novel and solid.2. Extensive benchmark analysis across various types of spatial data and tissues demonstrates superior performance of the method in spatial domain identification, pseudo-spatiotemporal analysis, and domain-specific gene discovery. The empirical evidence is very convincing.3. The method's application to chicken heart development, breast cancer, and lung cancer showcases its potential in reconstructing spatiotemporal lineage structures and delineating tumor microenvironments, highlighting its value in clinical research.Weaknesses:1. In Figure 4, SpaceFlow is the only baseline for the case study. However, the performance of SpaceFlow is not top-ranked in other experiments. There should be a justification for why SpaceFlow is highlighted here.2. The contribution of the global attention mechanism to the whole framework is not very clear. The authors may provide more intuition and empirical justification (e.g., ablation study) if they would like to highlight this design.3. By introducing the hyperparameters α, β and γ in Eq. (11), the method has a significantly larger search space than other methods. It is important to note how these hyperparameters are chosen in practice, more importantly, whether the test performance is referred when adjusting these hyperparameters. This might result in an unfair evaluation.

Level of Interest

Please indicate how interesting you found the manuscript: Choose an item.

Quality of Written English

Please indicate the quality of language in the manuscript: Choose an item.

Declaration of Competing Interests

Please complete a declaration of competing interests, considering the following questions:

- Have you in the past five years received reimbursements, fees, funding, or salary from an organisation that may in any way gain or lose financially from the publication of this manuscript, either now or in the future?
- Do you hold any stocks or shares in an organisation that may in any way gain or lose financially from the publication of this manuscript, either now or in the future?
- Do you hold or are you currently applying for any patents relating to the content of the manuscript?
- Have you received reimbursements, fees, funding, or salary from an organization that holds or has applied for patents relating to the content of the manuscript?
- Do you have any other financial competing interests?
- Do you have any non-financial competing interests in relation to this paper?

If you can answer no to all of the above, write 'I declare that I have no competing interests' below. If your reply is yes to any, please give details below.

I declare that I have no competing interests

I agree to the open peer review policy of the journal. I understand that my name will be included on my report to the authors and, if the manuscript is accepted for publication, my named report including any attachments I upload will be posted on the website along with the authors' responses. I agree for my report to be made available under an Open Access Creative Commons CC-BY license (http://creativecommons.org/licenses/by/4.0/). I understand that any comments which I do not wish to be included in my named report can be included as confidential comments to the editors, which will not be published.

Choose an item.

To further support our reviewers, we have joined with Publons, where you can gain additional credit to further highlight your hard work (see: https://publons.com/journal/530/gigascience). On publication of this paper, your review will be automatically added to Publons, you can then choose whether or not to claim your Publons credit. I understand this statement.

Yes Choose an item.
